# IrMn-Cluster-Based Artificial Metalloenzymes with Radiosensitized Systemic Antitumor Responses to Prevent Malignant Tumor Metastasis and Recurrence

**DOI:** 10.1007/s40820-026-02309-2

**Published:** 2026-07-27

**Authors:** Ruidan Li, Qinlong Wen, Zhenyu Xing, Ting Wang, Jing Yang, Yunfeng Tao, Shengdong Mu, Shuang Li, Zhigong Wei, Chong Cheng, Xingchen Peng

**Affiliations:** 1 Department of Biotherapy, Cancer Center, West China Hospital, Sichuan University, Chengdu, 610041 People’s Republic of China; 2 West China School of Public Health and Department of Oncology, West China Fourth Hospital, Sichuan University, Chengdu, 610041 People’s Republic of China; 3 State Key Laboratory of Advanced Polymer Materials, College of Polymer Science and Engineering, Sichuan University, Chengdu, 610065 People’s Republic of China; 4 College of Biological Engineering, Sichuan University of Science and Engineering, 188 University Town, Yibin, People’s Republic of China; 5 State Key Laboratory of Oral Diseases, Department of Endodontics, National Clinical Research Center for Oral Diseases, West China Hospital of Stomatology, Sichuan University, Chengdu, 610041 People’s Republic of China; 6 Nuclear Physics and Medical Research Key Laboratory of Sichuan Province, Sichuan University, Chengdu, 610041 People’s Republic of China

**Keywords:** Bioinspired nanomedicines, Artificial metalloenzymes, Reactive oxygen species, Antitumor therapy, Malignant tumor

## Abstract

Developing radiosensitizing agents to amplify tumor-eradicating effects on primary, regional recurrence, and distant metastases plays a transformative role in modern cancer care. Here, we report the de novo design of biocatalytic artificial metalloenzymes with an IrMn-cluster-based redox center (IMM) to achieve radiosensitized systemic antitumor responses for preventing malignant tumor metastasis and recurrence. Notably, our findings indicate that Mn-organic ligands substantially enrich the electron density of Ir clusters, thereby optimizing their interaction with oxygen species and markedly enhancing the production of both reactive oxygen species and molecular oxygen. When combined with radiotherapy, the IMM effectively amplifies DNA damage and induces pronounced apoptosis by alleviating intratumoral hypoxia. This shift reprograms the tumor microenvironment, enhancing radiosensitivity and facilitating the infiltration and activation of intratumoral CD8⁺ T cells and dendritic cells. Moreover, when integrated with anti-PD-1 therapy, this coordinated therapeutic regimen elicits potent systemic immune responses and durable antitumor memory, effectively suppressing tumor recurrence and metastasis while markedly improving therapeutic efficacy and long-term survival. We anticipate that this conceptual design could offer a promising and translationally relevant nanomedicine platform for radiotherapies.

**Supplementary Information:**

The online version contains supplementary material available at 10.1007/s40820-026-02309-2.

## Highlights

- The bioinspired design of IrMn-based redox centers (IMM) with rapid electron transfer can promote the formation of electron-rich IMM, thereby guaranteeing superior radio-enhanced reactive oxygen species biocatalysis and O_2_ generation.
- The tumor-adaptive and biocompatible IMM can induce mitochondrial dysfunction and hinder DNA repair, resulting in robust immunogenic cell death.
- An IMM-based biocatalyst can activate systemic antitumor responses and reverse a suppressive tumor microenvironment, thereby boosting tumoricidal effects and preventing metastasis and recurrence by synergizing with checkpoint blockade therapy.

**Supplementary Information:**

The online version contains supplementary material available at 10.1007/s40820-026-02309-2.

## Graphical abstract

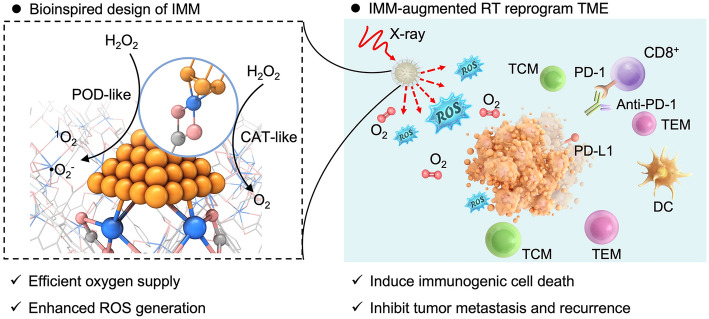

## Introduction

Cancer continues to pose a substantial global health burden, causing millions of deaths each year and remaining a major obstacle to public health worldwide [1, 2]. As a cornerstone of cancer treatment, radiotherapy (RT) exerts its antitumor effects by generating reactive oxygen species (ROS) and inducing DNA damage. Notably, its integration with immune checkpoint blockade has redefined treatment outcomes in patients with refractory, relapsed, or metastatic disease, underscoring its transformative role in modern cancer care [3–5]. While driven by factors such as tumor heterogeneity, cancer stem cells, and metabolic reprogramming, intrinsic and acquired radiation resistance substantially limit the therapeutic effectiveness of RT in certain solid tumors [6–8]. Tumor hypoxia represents a major contributor to radioresistance [9, 10]. Under hypoxic conditions, radiation-induced DNA radical intermediates remain reversible and are readily quenched, preventing oxygen fixation and markedly reducing the formation of lethal DNA double-strand breaks. Conversely, alleviating hypoxia restores the oxygen-fixation effect, stabilizing these radicals and thereby preventing irreversible damage and enhancing tumor radiosensitivity [11–13]. One potential approach to mitigating these challenges is to develop radiosensitizing agents that amplify tumor-eradicating effects against primary tumors, regional recurrences, and distant metastases. Despite extensive development, currently available radiosensitizers, including lanthanide-, hafnium-, and gold-based nanomaterials, still suffer from insufficient tumor ablation capability, limited activation of systemic antitumor immunity, inadequate biodegradability, and potential biosafety concerns [14–17].

The development of next-generation radiosensitizing materials for clinical RT faces two principal hurdles: achieving high biocompatibility and biodegradability while enhancing X-ray energy deposition and ROS generation; and amplifying ROS and O_2_ production to alleviate tumor hypoxia, overcome radioresistance, minimize material dosage, and trigger systemic antitumor responses, collectively contributing to improved therapeutic outcomes and suppression of metastasis and recurrence [18–21]. Recent advances in artificial peroxidase design have focused on exploiting the acidic, hydrogen peroxide (H_2_O_2_)-rich tumor microenvironment (TME) to amplify ROS and O_2_ supply, with particular emphasis on biologically active forms including superoxide anions (•O_2_^−^) and singlet oxygen (^1^O_2_) [22–25]. Nonetheless, engineering artificial peroxidases capable of efficiently producing ROS and O_2_ remains a formidable challenge, owing to the complexity of multielectron redox processes involving H_2_O_2_ and reactive intermediates, the transient evolution of reactive intermediates, and the high energy barrier associated with the desorption of oxygen species [26–28]. In addition, the widespread reliance on metal-based or inorganic frameworks in current artificial peroxidase designs raises pressing concerns over their biocompatibility and long-term safety, particularly for use as next-generation radio-responsive therapeutic agents [29–32].

Manganese (Mn)-related enzymes, including peroxidase (POD) and catalase (CAT), play an essential role in the biocatalytic process [24, 33–35], which can produce or degrade the ROS and O_2_ levels to modulate the inflammatory signaling and hypoxic stress [36–38]. Iridium (Ir), a high atomic number metal, exhibits favorable biocompatibility and distinctive physicochemical characteristics, including abundant vacant orbitals and excellent redox robustness, thereby facilitating effective ROS and O_2_ production through accelerated electron transfer with H_2_O_2_ as the substrate [39–41]. To address the limitations of unbalanced multielectron reactions involving H_2_O_2_ and oxygen radicals, we propose integrating Mn-oxygen-organic ligand linker units with Ir metals through organic ligand coordination, mimicking the architecture and catalytic centers of natural enzymes. This approach is expected to facilitate reversible catalytic cycles, thereby promoting the generation of ROS and O_2_, which hold significant potential as radiosensitizing agents to advance tumor RT and immunotherapy. Moreover, compared with natural enzymes, artificial metalloenzymes possess several inherent advantages, including tunable composition and structure, programmable catalytic activity, favorable environmental stability, and relatively low production costs [42, 43]. Therefore, the development of an iridium–manganese cluster-based artificial metalloenzyme may overcome the intrinsic limitations of natural enzymes in stability, large-scale production, and multifunctional integration. To the best of our knowledge, studies exploring this strategy remain scarce.

**Fig. 1:**
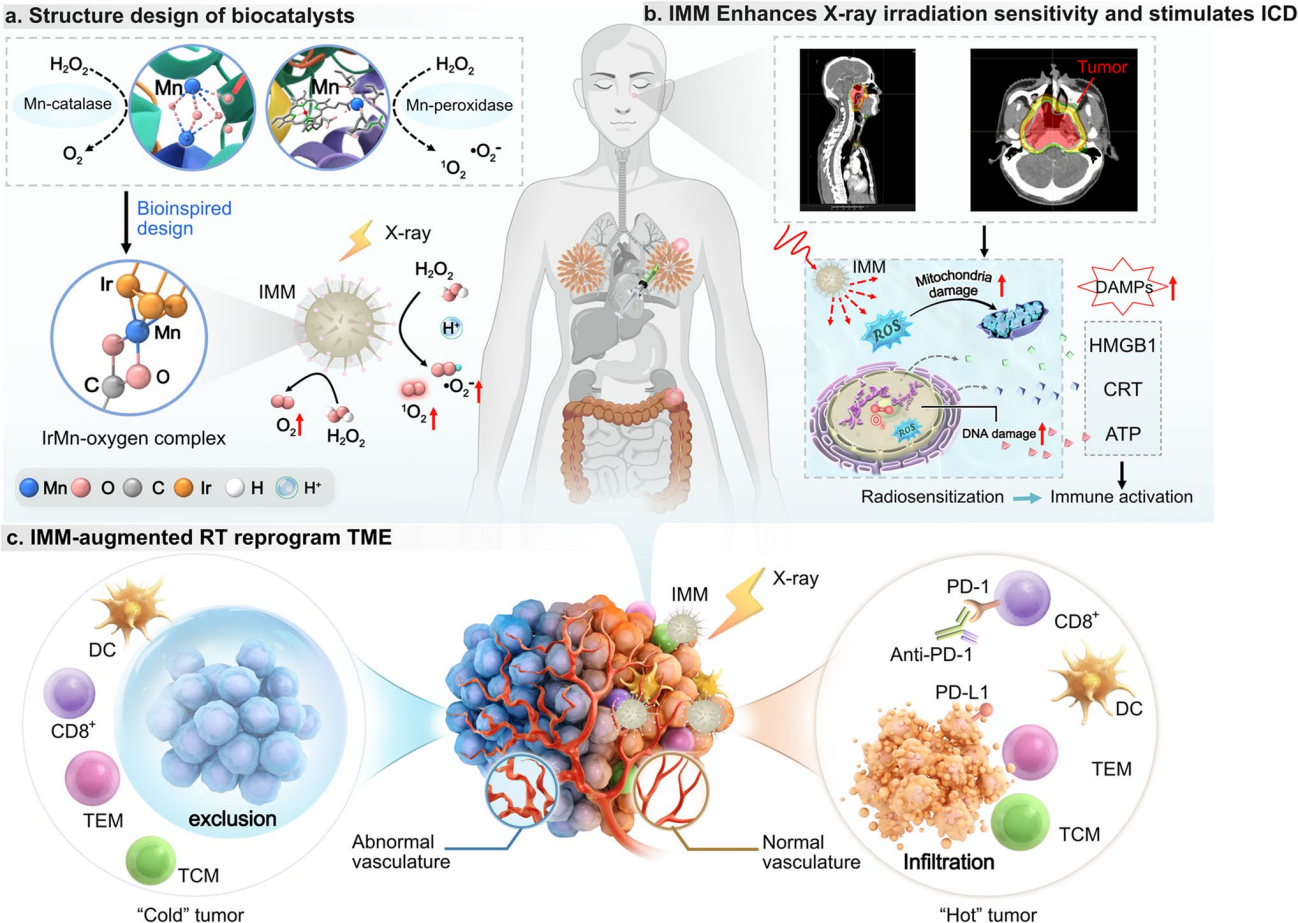
Schematic illustration of IMM as an effective radiosensitive agent to achieve radiosensitized systemic antitumor responses for preventing malignant tumor metastasis and recurrence. **a** Bioinspired design of IMM with efficient ROS- and O_2_-generation centers. **b** Mechanism of IMM-mediated radiosensitization: inducing mitochondrial dysfunction, hindering DNA damage repair, and triggering robust ICD. **c** IMM-induced cancer radiodynamic therapy and metastatic recurrence prevention: systemic antitumor responses activation, tumor vascular normalization, and immune cell infiltration

**Fig. 2:**
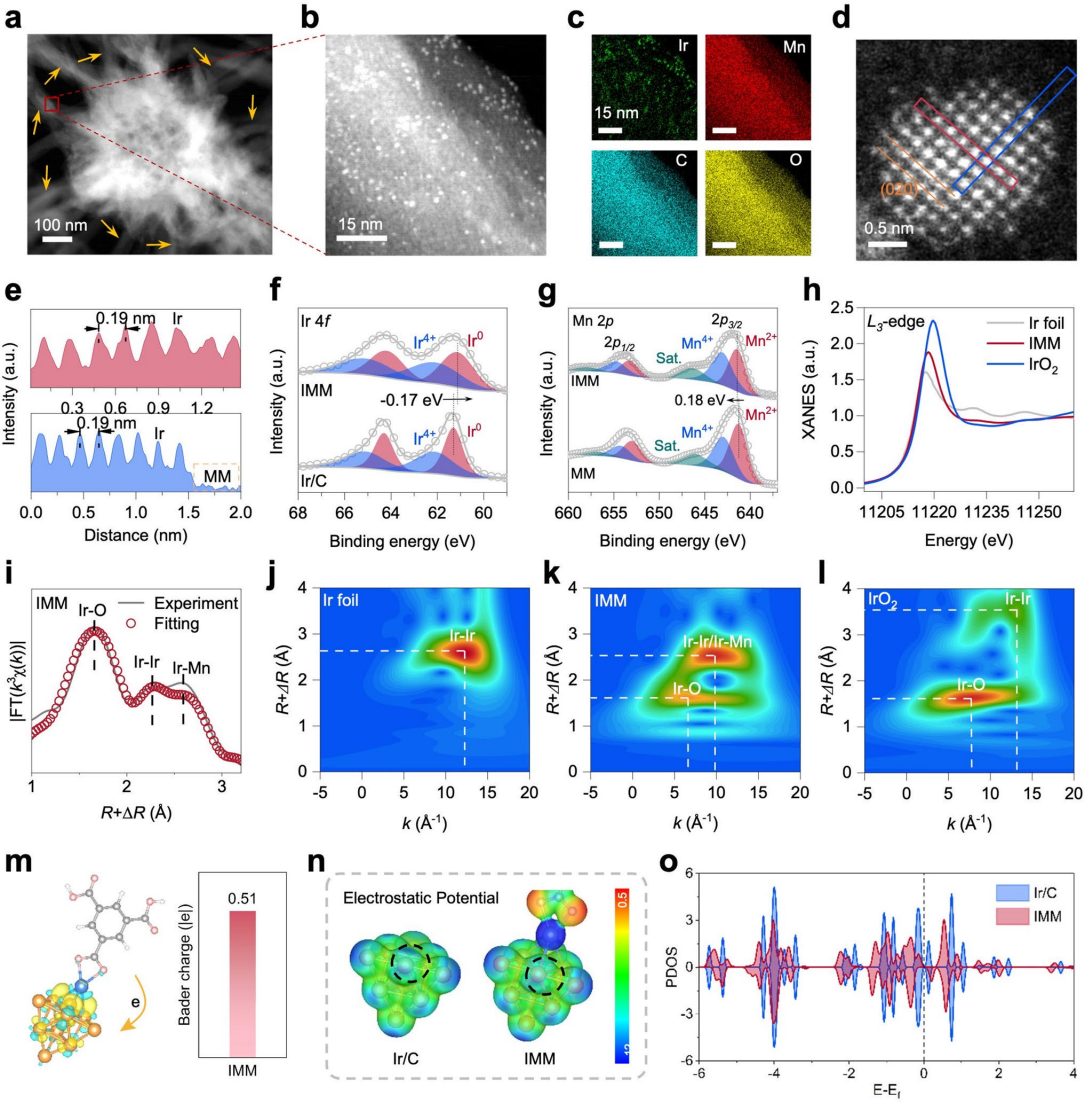
Synthesis and characterization of IMM. **a** HAADF-STEM image of IMM, showing the spiky morphology indicated by yellow arrows. **b** Enlarged HAADF-STEM image and **c** corresponding EDS elemental mapping of IMM. **d** Atomic-resolution HAADF-STEM image of IMM. **e** Intensity profiles of IMM labeled with red and green boxes in **d**. The XPS survey scan spectra of MM, IMM in **f** Ir 4*f*, **g** Mn 2*p* regions. **h** Normalized XANES spectra at Ir *L*_*3*_-edge. **i** Experimental and fitted EXAFS spectra of IMM. WT-EXAFS mappings at the Ir *L*_*3*_-edge for the** j** Ir foil, **k** IMM, and **l** IrO_2_. **m** Differential charge-density distribution and Bader charge evaluation of IMM (cyan and yellow denote electron depletion and enrichment regions, respectively; the isosurface threshold for charge-density difference was set to 0.003 e Bohr^−3^). **n** Electrostatic potential diagrams of Ir/C and IMM. **o** PDOS analysis of Ir-*d* orbital of Ir/C and IMM. a.u. indicates the arbitrary units. Experiments were repeated independently (**a**–**d**) three times with similar results. (Color figure online)

Here, drawing inspiration from the catalytic features of natural Mn-POD and Mn-CAT, we report the de novo design of highly active, robust, and electron-rich IrMn-cluster-based artificial metalloenzymes (IMM) to achieve radiosensitized systemic antitumor responses for preventing malignant tumor metastasis and recurrence. As depicted in Fig. 1, the primary objective of this work originates from three aspects: 1) The bioinspired design of IMM with rapid electron transfer can promote the formation of electron-rich Ir redox centers to guarantee the superior radio-enhanced ROS biocatalysis and O_2_ generation (Fig. 1a); 2) the biocompatible and tumor-adaptive IMM can induce mitochondria dysfunction and hinder DNA damage repair to result in robust immunogenic cell death (ICD; Fig. 1b); and 3) the IMM-based biocatalyst can activate the systemic antitumor responses and reverse the suppressive TME, thus boosting tumoricidal effects and preventing metastasis and recurrence via synergizing checkpoint blockade therapy (Fig. 1c). Our results demonstrate that IMM disrupts DNA repair, alleviates intratumoral hypoxia, and promotes pronounced tumor cell apoptosis, thereby markedly enhancing cellular responsiveness to X-ray irradiation. In parallel, the combination of IMM and RT reshapes the immunosuppressive TME, enhancing radiosensitization and converting “cold tumors” into “hot tumors.” Critically, integration with anti-PD-1 transforms this approach into a systemic therapeutic paradigm, eliciting robust abscopal responses and durable antitumor memory that collectively suppresses metastatic recurrence while markedly improving long-term survival outcomes. Correspondingly, the therapeutic superiority of IMM has been validated in the spontaneous lung metastasis breast cancer animal model and the RT-resistant patient-derived xenograft (PDX) model. Remarkably, our innovative design exhibits exceptional biocatalytic activity and effective radiosensitizing capability, demonstrating substantial therapeutic potential for suppressing both primary and metastatic malignancies, particularly highly aggressive tumors.

## Experimental Section

### Materials

The Mn(NO_3_)_2_·4H_2_O, 3,3,5,5-tetramethylbenzidine (TMB), trimesic acid, hydroethidine (HE), 9,10-diphenanthraquinone (DPA), 5,5-dimethyl-1-pyrroline N-oxide (DMPO), and 2,2,6,6-tetramethylpiperidine (TEMP) were obtained from Aladdin reagents (Shanghai, China), iridium (III) chloride hydrate (IrCl_3_ •xH_2_O) was acquired from Energy Chemical (Shanghai, China), and Ir/C (20% wt%) was obtained from Premetek Co. (NJ, USA). All other chemicals were utilized directly without additional purification, and deionized pure water (18.2 MΩ·cm) used in the experiments was produced from a Milli-Q Academic system (Millipore Corp., Billerica, MA, USA). The in vivo anti-mouse PD-1 antibody (Ch15mt) was sourced from BeiGene.

### Preparation of Mn-Organic Precursor and IMM

#### Synthesis of Mn-Organic Precursor

First, 192 mg Mn(NO_3_)_2_·4H_2_O was dissolved in 25 mL of ethanol to obtain solution A. To prepare solution B, 87.5 mg trimesic acid was introduced into 25 mL of ethanol. Then, solution B is added to solution A to form a homogeneous mixture. After stirring for 30 min, the resulting mixture was transferred to a 100-mL Teflon-lined stainless steel autoclave and heated to 160 °C for 12 h. The resulting powder was collected by filtration, rinsed three times with ethanol, and subsequently dried under vacuum at 60 °C for 12 h.

#### Synthesis of IMM

MM (50 mg) was initially redispersed in 40 mL of ethanol under stirring for 30 min, followed by the addition of 0.5 mL of an aqueous solution of IrCl_3_·xH_2_O (10 mg mL^−1^) to the resulting suspension. Subsequently, the mixture was transferred to a 100-mL Teflon-lined stainless steel autoclave and heated to 160 °C for 12 h. After cooling to room temperature, the collected solid was isolated by filtration, rinsed three times with ethanol, and dried under vacuum at 60 °C for 12 h.

## Results and Discussion

### Design and Structure Characterizations of Biocatalytic IMM

The IMM material, featuring catalytically active IrMn clusters coordinated to a Mn-organic complex, was synthesized via a sequential reaction protocol. Initial formation of Mn-organic precursors (MM) was achieved through a one-step hydrothermal reaction between Mn salts and 1,3,5-benzenetricarboxylic acid. Subsequent incorporation of Ir species through adsorption–exchange–nucleation pathways yielded the final IMM complex. Inspired by the catalytic mechanisms of Mn-organic ligands in natural Mn-POD, we hypothesize that this bioinspired IMM complex exhibits multiple advantages. These features include (i) electron-donating MM ligands that participate in redox processes, (ii) electron-rich Ir clusters that effectively promote *OOH intermediate desorption, (iii) spatially organized organic ligands in the IMM complex enabling efficient enzymatic-like activation of both ROS and O_2_, and (iv) high X-ray irradiation sensitivity. X-ray diffraction characterization reveals that both MM and IMM exhibit amorphous structural features (Fig. S1). And, no diffraction peaks corresponding to metallic Ir are observed in the IMM, indicating that large-sized Ir particles were not formed.

High-angle annular dark-field scanning transmission electron microscopy (HAADF-STEM) and scanning electron microscopy (SEM) images reveal that IMM adopts a spiky morphology and dispersibility (Figs. 2a, S2, and S3). In contrast, the pristine MM exhibits a spherical structure (Fig. S3). IrCl_3_·xH_2_O hydrothermal treatment induces Mn-MOF surface reconstruction through partial etching, dissolution–reprecipitation, and Ir^3+^-driven lattice strain, promoting anisotropic facet-selective growth and transforming spherical particles into spiky nanostructures [44–46]. Dynamic light scattering data show that the average hydrodynamic diameters of MM and IMM were 531 and 615 nm, respectively. After 10 days, they still had good dispersibility and stability in water (Fig. S4). Energy-dispersive spectroscopy (EDS) elemental mapping analysis reveals the presence of Ir, Mn, C, and O, with Ir clusters homogeneously distributed across the IMM surface (Fig. 2b, c). Following atomic-resolution HAADF-STEM imaging, the proposed MM-coordinated Ir sites were validated. High-density, ultrasmall Ir nanoclusters, with an average size of approximately 1.5 nm, are homogeneously dispersed on the surface of MM (Fig. S5). Atomic-resolution HAADF-STEM characterization provides visual evidence of the well-ordered periodic distribution of Ir sites (Fig. 2d). Clear lattice fringes with a spacing of 0.19 nm (Fig. 2e) are observed, corresponding to the (020) facets of Ir nanoclusters on IMM.

Afterward, X-ray photoelectron spectroscopy (XPS) was performed to characterize the valence distribution and electronic configuration of the biocatalytic IMM (Fig. S6). The XPS characterization reveals the presence of Ir, Mn, C, and O elements, with an Ir atomic percentage of 1.27 at% in IMM. The high-resolution O 1*s* spectra reveal characteristic metal-O signals in both IMM and MM, further confirming the successful construction of MM (Figs. S7 and S8). The high-resolution Ir 4*f* spectra of IMM are principally deconvoluted into two doublets centered at 61.13/64.18 and 62.09/65.08 eV, assigned to Ir^0^ and Ir^4+^, respectively, indicating that the Ir precursor is partially reduced to metallic Ir [47, 48]. Notably, the Ir 4*f* spectra of IMM exhibit a core level shift of − 0.17 eV in the 4*f* binding energy compared to carbon-supported Ir sites (Fig. 2f). Concurrently, the Mn species in IMM display a positive core level shift of 0.18 eV in the binding energy of Mn 2*p* compared to MM, and the Mn^4+^/Mn^2+^ intensity ratio in IMM (1.03) is much higher than that of the pristine MM (0.74) (Fig. 2g and Table S1). These results indicate electron migration from the electron-rich MM framework to the Ir cluster centers. Such charge redistribution is expected to promote multielectron conversion processes of oxygen-related intermediates on Ir sites, thereby accelerating the overall redox kinetics.

The X-ray absorption near-edge structure (XANES) and extended X-ray absorption fine structure (EXAFS) analyses were conducted to characterize the local coordination configuration of Ir centers in the biocatalytic IMM. The Ir *L*_*3*_-edge XANES spectra reveal that the white-line intensity of IMM lies between those of Ir foil and IrO_2_, corresponding to an average Ir oxidation state of + 1.58 and suggesting the existence of partially oxidized Ir nanoclusters within the biocatalytic system (Figs. 2h and S9). EXAFS fitting and wavelet transform (WT) further revealed average coordination numbers of 2.93, 6.33, and 4.00 for Ir–O, Ir–Ir, and Ir–Mn in IMM, respectively, with corresponding bond lengths of 2.00, 2.68, and 3.70 Å (Figs. 2i–l, S10, S11, and Table S3). The close agreement between these experimental parameters and the theoretical model supports the proposed atomic configuration of IMM. Then, density functional theory (DFT) calculations were carried out to probe the electronic interactions between Ir and the ligands. As shown in Fig. 2m, a significant accumulation of electron density is observed at the interface between Ir and the ligand, with approximately 0.51 |e| transferred from the ligand to Ir, indicating a strong electron interaction. Electrostatic potential (ESP) and projected density of states (PDOS) plots show that Ir centers in IMM exhibit a higher electron density than those in Ir/C due to the incorporation of the ligand (Fig. 2n, o). The elevated electron density at Ir centers facilitates multielectron redox conversion processes involving oxygen-related species.

### Enzyme-Mimetic ROS-Biocatalytic Activities

**Fig. 3:**
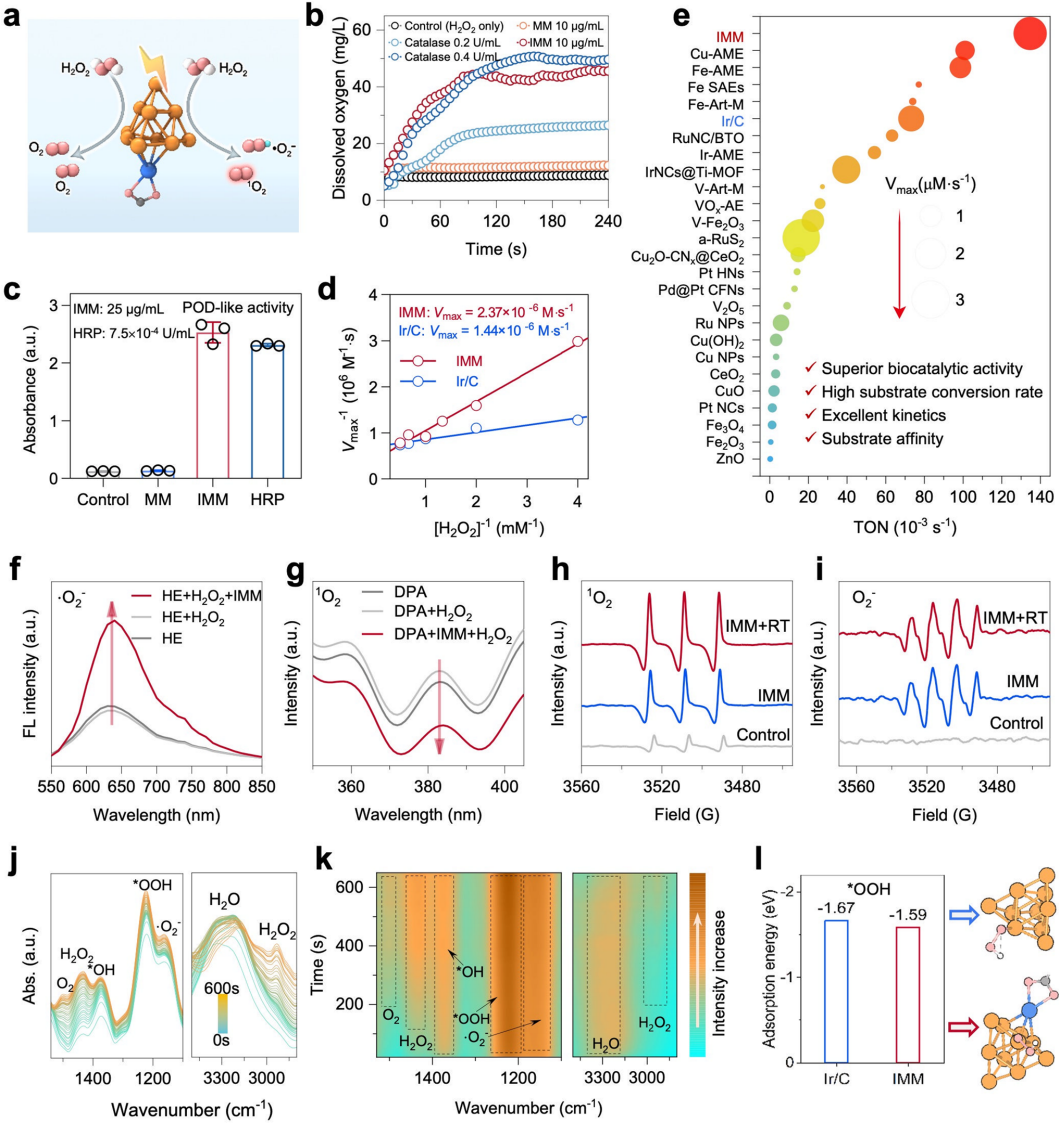
Detection of the ROS- and O_2_-related biocatalytic performances. **a** Schematic illustration of the dual functions of IMM in O_2_ and ROS generation. **b** The dissolved O_2_ concentration was quantified using a dissolved oxygen meter in the presence of biocatalysts and H_2_O_2_. **c** POD-mimicking activities of various catalysts (n = 3 independent experiments, data are presented as mean ± S.D.), POD: peroxidase. **d** Double-reciprocal plots for determining the kinetic constants of IMM and Ir/C for different concentrations of H_2_O_2_. **e** Comparison of the TON and *V*_max_ values with reported enzyme-mimetic catalysts. **f** •O_2_^−^ detected by HE. **g** ^1^O_2_ detected by DPA. EPR spectra for recording the **h** ^1^O_2_ signal and the **i** •O_2_^−^ signal. **j** In situ FTIR spectrum and **k** the corresponding contour plot of IMM for the POD-like process. **l** Calculated adsorption energy of *OOH on Ir/C and IMM. Ir, yellow; Mn, blue; C, khaki; H, white; O, pink. *K*_m_ is the Michaelis constant, and TON is the turnover number. a.u. indicates the arbitrary units. (Color figure online)

Guided by structural and electronic analyses of the synthesized IMM, particularly the coordination interactions between the Ir clusters and the MM ligands, we systematically investigated its biomimetic catalytic performance. We further hypothesized that IMM possesses dual catalytic functionality by concurrently facilitating oxygen evolution and efficient ROS generation. Notably, under X-ray irradiation, it markedly amplifies ROS generation, thereby enhancing radio-responsive oxidative stress, alleviating radioresistance, and improving overall antitumor response (Fig. 3a).

Under the mimicking TME (pH 6.5 with H_2_O_2_) conditions, the IMM catalyst (10 μg mL^−1^) exhibits exceptional O_2_-generation activity, achieving 44.54 mg L^−1^ O_2_ within 100 s (Fig. 3b), which is equivalent to 0.4 U mL^−1^ of natural CAT activity. Evaluation of POD-like activity through 3,3′,5,5′-tetramethylbenzidine (TMB) assays demonstrates the ROS generation of the IMM biocatalyst (25 μg mL^−1^) (Figs. 3c and S12), which is about equivalent to 7.5 × 10^–4^ U mL^−1^ of natural horseradish peroxidase (HRP) activity. Subsequent kinetic analysis of the POD-mimetic process was conducted using Michaelis–Menten analysis, enabling the quantification of key catalytic parameters, including the Michaelis constant (*K*_m_), maximum reaction velocity (*V*_max_), and turnover number (TON), which reflects the highest substrate turnover per catalytic site (Fig. 3d). Compared with Ir/C, the IMM presents substantially higher *V*_max_ (2.37 μΜ s^−1^) and TON (134.7 × 10^–3^ s^−1^), indicating enhanced catalytic kinetics (Table S2). Significantly, the IMM exhibits superior catalytic efficiency, achieving the highest TON values that surpass those of recently reported biocatalysts (Fig. 3e and Table S4).

The types of ROS, ^1^O_2_ and •O_2_^−^, generated by IMM in the presence of H_2_O_2_ were confirmed through the 9,10-diphenanthraquinone (DPA) and dihydroethidium (HE) probes, respectively (Fig. 3f, g). Electron paramagnetic resonance (EPR) spectroscopy and the o-phenylenediamine (OPD) colorimetric assay further demonstrate a marked increase in ROS production under X-ray irradiation (Figs. 3h, i and S13). The enhanced ROS production is primarily attributed to the synergistic Ir–Mn catalytic site and its cooperative activation under X-ray irradiation. In situ Fourier-transform infrared (FTIR) spectroscopy captures key structural transformations during the POD-like process, revealing the formation of reactive intermediates, including •O_2_^−^, *OOH, and *OH species (Fig. 3j, k).

The *OOH intermediate serves as a pivotal species in both ROS (•O_2_^−^ and ^1^O_2_) and O_2_ generation pathways [49–51], making it an ideal probe for elucidating the structure–activity relationship between Ir cluster coordination and catalytic performance. The generation of ^1^O_2_ depends on the further transformation of *OOH. According to the Russell mechanism, two adsorbed superoxide radicals (*OOH or *•O_2_^−^) can generate ^1^O_2_ via a recombination reaction [52–54]. Density functional theory (DFT) calculations reveal that ligand-induced electronic modulation of Ir clusters in IMM yields a *OOH adsorption energy of − 1.59 eV, weaker than the − 1.67 eV observed for conventional Ir/C (Fig. 3l). This strategically reduced binding strength, attributable to enhanced electron density at the Ir active sites, promotes intermediate desorption and, consequently, enhances catalytic turnover. The DFT-validated electronic structure optimization thus establishes a link between the Mn-organic ligand and the dual-functional catalytic performance in ROS/O_2_ generation. In addition to *OOH, the appearance of *OH in the in situ FTIR spectra. Under acidic TME-mimicking conditions, H_2_O_2_ can adsorb onto the Ir–Mn catalytic sites and form surface *OOH. Subsequent proton-coupled electron transfer and O–O bond polarization may promote cleavage of the adsorbed peroxide species, generating surface-bound *OH intermediates. This process is consistent with the Fenton-like O–O cleavage pathway commonly proposed for POD-like nanozymes [55–57]. Therefore, the observed *OH signal is mechanistically meaningful, as it indicates peroxide bond activation and represents a key oxidative intermediate responsible for substrate oxidation and ROS amplification. These results suggest that OOH functions as a central bifurcating intermediate: Its further transformation can contribute either to •O_2_⁻/^1^O_2_ and O_2_ generation or to O–O bond cleavage toward OH/•OH formation, collectively accounting for the dual ROS/O_2_-generating activity of IMM.

### Antitumor Activity of IMM In Vitro

**Fig. 4:**
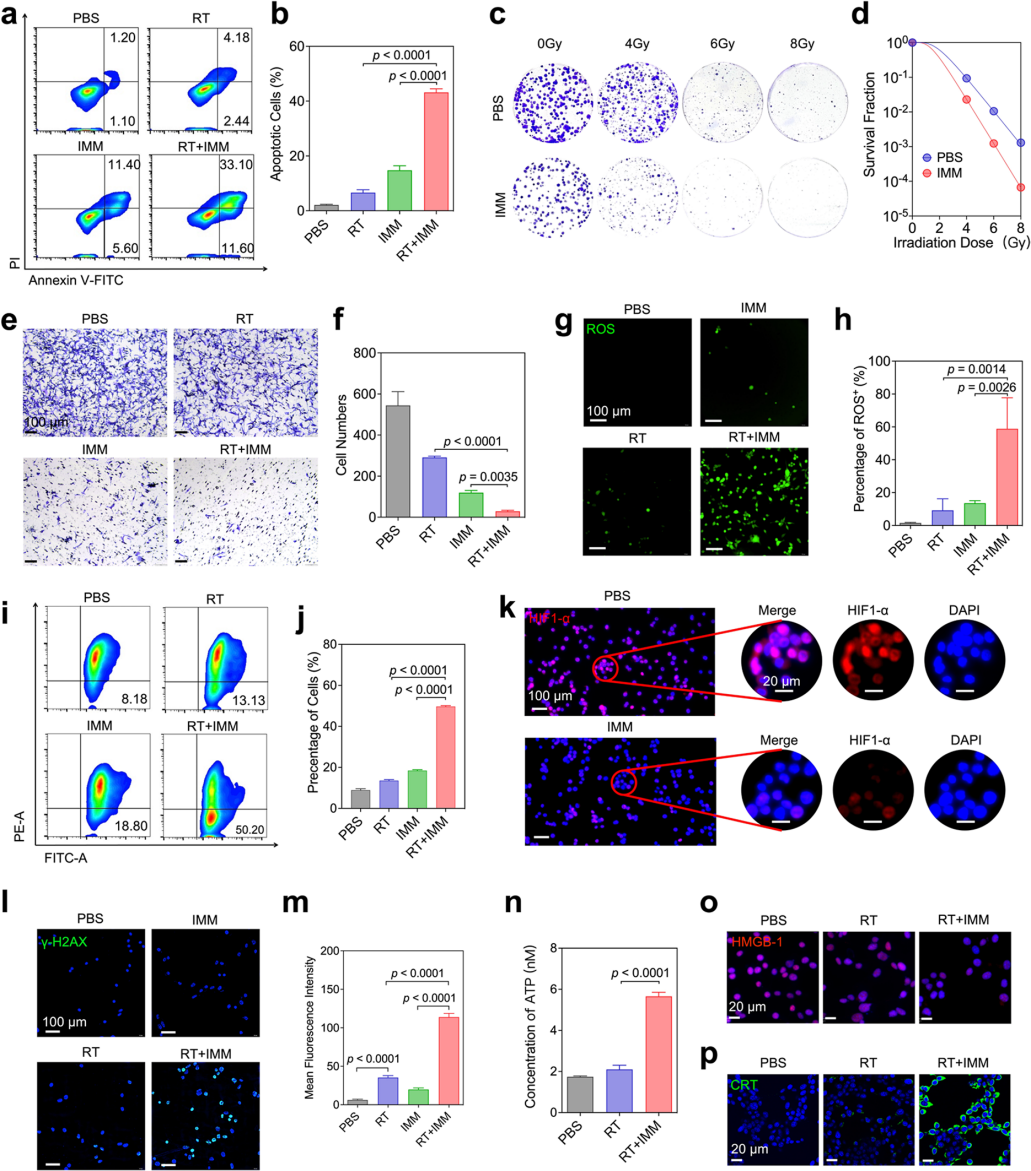
Antitumor activity of IMM in vitro under hypoxic conditions. **a** and **b** Annexin V/PI staining of CT26 cells and quantification of apoptotic cell percentages in each group (n = 3 independent replicates). **c** and** d** Representative images from the clonogenic assay evaluating the radio-enhancement effect of IMM on CT26 cells (n = 3 independent replicates). Experiments were independently repeated three times. **e** and **f** Representative images from the transwell migration assay evaluating the cell migration and the numbers of migrated cells in different groups (n = 3 independent replicates; scale bar = 100 μm). Experiments were independently repeated three times. **g** Representative fluorescence images and **h** quantitative flow cytometric analysis of ROS levels in CT26 cells across different groups (n = 3 independent replicates; scale bar = 100 μm). Experiments were independently repeated three times. **i** and **j** Quantitative flow cytometry of the JC-1 signal among cells following different treatments (n = 3 independent replicates). **k** Representative fluorescence images showing HIF-1α expression in CT26 cells treated with PBS or IMM (scale bar = 100 μm, and scale bar = 20 μm in magnified images). Immunofluorescence experiments were independently repeated three times. **l** Representative fluorescence images of γ-H2AX expression (scale bar = 100 μm) and **m** quantification of mean γ-H2AX fluorescence intensity across groups. Immunofluorescence experiments were independently repeated three times. **n** ATP release from CT26 cells in different groups (n = 3 independent replicates). **o** and **p** Representative fluorescence images of HMGB1 and CRT expression in CT26 cells after different treatments (scale bar = 20 μm). Immunofluorescence experiments were independently repeated three times. Data are presented as mean ± S.D. Statistical significance was determined using one-way ANOVA for multiple-group comparisons, followed by Tukey’s two-tailed post hoc test for pairwise analysis

Radioresistance, a major clinical challenge in oncology that often leads to tumor recurrence and metastasis, is partly driven by hypoxia within the TME [58, 59]. In this context, the synthesis of IMM, with robust biocatalytic activity and unique structural advantages, has sparked our interest in its potential as a biocatalytic agent and as a radiosensitizing tumor-killing nanomedicine. The antitumor performance of IMM was evaluated using CT26 colon carcinoma cells under hypoxic conditions. CT26 cells were treated with MM or IMM, followed by 6 Gy X-ray irradiation, and apoptosis levels were determined by flow cytometry 48 h after treatment. Quantitative analysis of three independent experiments demonstrated that the RT + IMM group exhibited the highest apoptosis rate, 43.17% ± 1.33%, among all treatment groups (Figs. 4a, b and S14). Calcein-AM/PI staining further confirmed that RT + IMM induced the highest level of cell death in CT26 cells compared with all other groups (Fig. S15).

Cell proliferation and migration are key indicators for assessing tumor recurrence, metastasis, and the effectiveness of radiodynamic therapy [60, 61]. Following previous findings on IMM’s antitumor activity, we further evaluated its effects on tumor cell migration and proliferation to assess its potential to reduce the occurrence of both tumor metastasis and recurrence. The proliferation of tumor cells following various treatments was evaluated using colony-forming assays. Following 4-h incubation with MM or IMM, SCC-7 squamous carcinoma cells were irradiated with graded doses of X-rays (0–8 Gy). The cells were then reseeded and allowed to grow for 8 days to form colonies, which were subsequently quantified to determine survival fractions (Fig. 4c). At the same manganese concentration, IMM outperforms MM in radiosensitization (Figs. 4d and S16). Additionally, a transwell migration assay was used to assess the migratory potential of the tumor cells. By quantifying the ability of cells to migrate through the membrane pores, this assay provides insight into their migration behavior under various treatment conditions, thereby facilitating a deeper understanding of the inhibitory effects of IMM on tumor cell invasiveness and metastatic potential [62]. The results show that RT + IMM in CT26 cells leads to a significant decrease in cell migration (Figs. 4e, f, and S17). The results demonstrate that IMM enhances the cytotoxicity of RT on tumor cells while significantly inhibiting their migration and proliferation. IMM not only improves RT efficacy but also plays a crucial role in inhibiting tumor metastasis and promoting tumor cell elimination, thereby strengthening the potential for successful RT application.

In this context, the potent antitumor effects observed with the IMM and RT combination can be attributed to IMM’s exceptional capabilities in ROS biocatalysis and O_2_ generation. IMM enhances therapeutic outcomes through a dual mechanism: generating ROS to directly damage critical tumor cell components and organelles, and alleviating TME hypoxia by producing O_2_, effectively overcoming radioresistance [63–65]. This synergistic action significantly enhances tumor cell sensitivity to RT and amplifies its therapeutic efficacy, underscoring IMM’s potential to advance cancer treatment paradigms. Next, we tested the ROS and O_2_ generation by IMM upon X-ray irradiation in CT26 cells under hypoxic conditions. The production of ROS was determined by the ROS probe 2’,7’-dichlorodihydrofluorescein diacetate (DCFH-DA), and the RT + IMM-treated cells show the strongest ROS generation both in quantitative flow cytometric analysis and fluorescence images (Figs. 4g, h and S18). The increase in ROS generation and the decrease in cellular ATP synthesis (Fig. S19) are indicators of mitochondrial dysfunction [66, 67]. The mitochondrial membrane-permeable dye JC-1 was used to assess mitochondrial membrane potential, specifically focusing on depolarization as an indicator of mitochondrial dysfunction. The results reveal that RT + IMM combination therapy induced significant mitochondrial dysfunction, underscoring its impact on cellular energy homeostasis and viability (Fig. 4i, j) [68, 69].

The O_2_ generation by IMM in vitro was probed with [Ru(bpy)_3_]Cl_2_, whose red fluorescence is inversely related to O_2_ concentration [70]. Under hypoxic conditions, the red fluorescence of [Ru(bpy)_3_]Cl_2_ is maintained in the PBS, RT, MM, and RT + MM groups but is nearly completely quenched in CT26 cells treated with IMM (Fig. S20. Furthermore, the reversal of hypoxia in CT26 cells by IMM was demonstrated by assessing HIF-1α expression, which is upregulated in cells at low O_2_ concentrations [19]. As shown, high HIF-1α signals are shown in the PBS and IMM-treated CT26 cells under hypoxic conditions, but IMM-treated CT26 cells show negligible HIF-1α signals (Fig. 4k). These results indicate that IMM reverses the hypoxia in CT26 cells under hypoxic conditions by decomposing H_2_O_2_ to generate O_2_. The X-ray radiation induces DNA damage (i.e., double-strand breaks, DSBs) by generating a radical on the DNA (DNA⋅), which reacts with O_2_, leading to the fixation or permanentization of the damage [71]. DSBs were quantified by γ-H2AX staining. (Figs. 4l, m and S21). Upon incubation with IMM followed by X-ray irradiation, CT26 cells showed pronounced γ-H2AX nuclear fluorescence, suggesting elevated levels of DNA damage. To characterize the dynamics of DNA damage repair, we quantified DNA DSBs at multiple post-treatment time points (Fig. S22). DSB levels in the RT and RT + MM groups showed a gradual decline over time, indicating ongoing repair. In contrast, the RT + IMM group maintained consistently high DSB levels with no detectable reduction, demonstrating a pronounced suppression of DNA repair.

Immunogenic cell death (ICD) is initiated by damage-associated molecular patterns (DAMPs) such as ATP, high mobility group protein box 1 (HMGB1), and calreticulin (CRT), which serve as “eat me” signals [72]. The ATP release was measured by a luciferin-based ATP assay. In comparison with the other groups, the RT + IMM-treated group shows the highest ATP release levels (Fig. 4n). After RT + IMM treatment, there is a significant decrease in nuclear HMGB1 (red fluorescence) and a notable increase in surface CRT (green fluorescence) of the cell membrane (Fig. 4o, p). Overall, the RT + IMM combination treatment demonstrates a robust ability to induce ICD in CT26 cells, primarily by enhancing the release of DAMPs, including ATP, HMGB1, and CRT. These DAMPs play a pivotal role in activating antigen-presenting cells, thereby stimulating adaptive antitumor responses [73]. By synergistically combining the cytotoxic effects of RT with IMM’s capacity to modulate the TME, this approach not only improves localized tumor control but also promotes the activation of systemic antitumor immunity. These findings underscore the dual therapeutic advantages of RT + IMM, providing a foundation for its application in advanced checkpoint inhibitor therapy.

### IMM-Augmented RT Against Tumor Progression with Robust Antitumor Responses

**Fig. 5:**
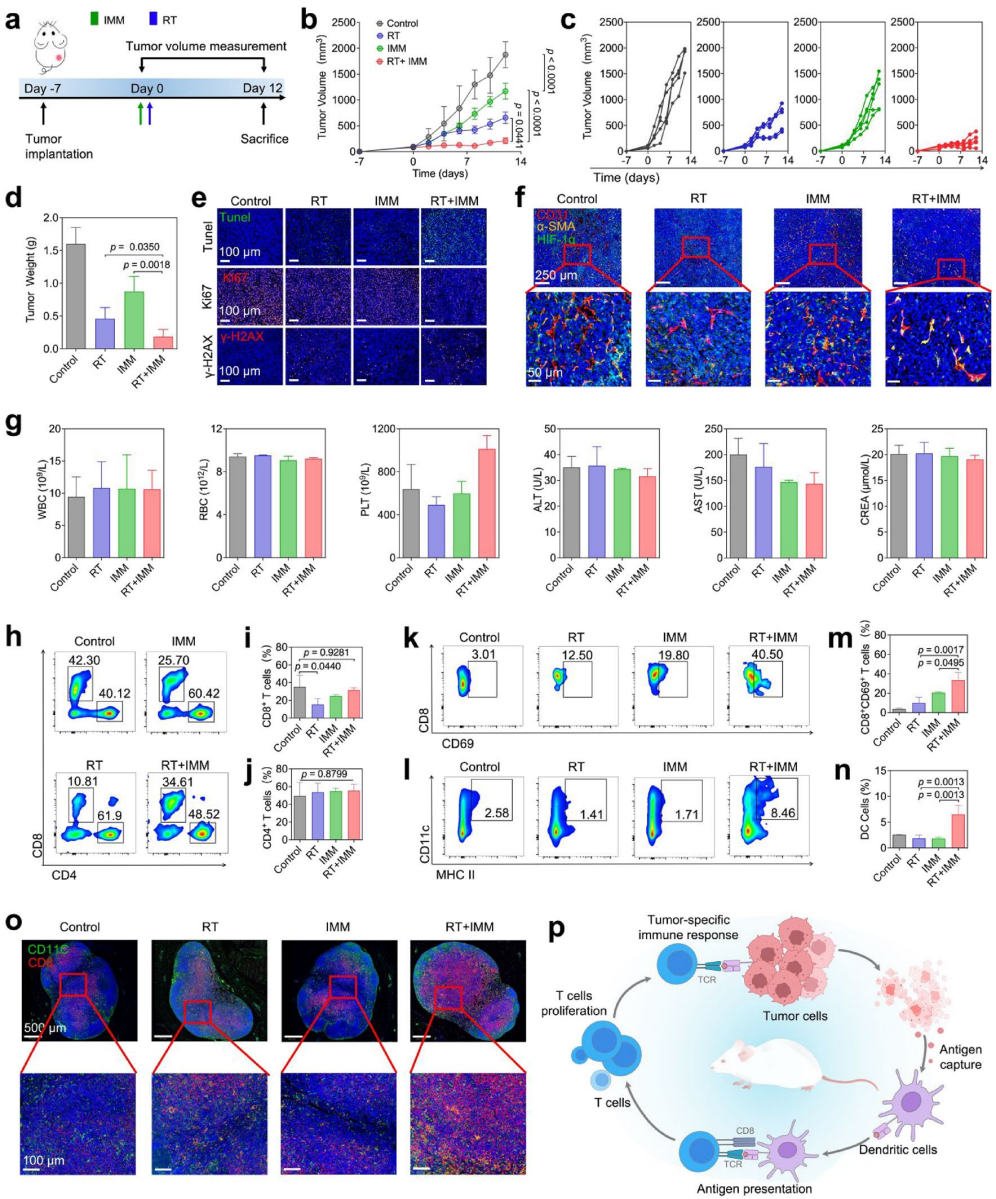
IMM-augmented RT against tumor progression. **a** Schematic illustration of IMM-augmented RT treatment (n = 5 biologically independent mice per group). **b** Average tumor growth curves, **c** individual tumor growth kinetics, and **d** tumor weight of CT26 tumor-bearing mice after different treatments (n = 5 independent replicates). **e** Representative immunofluorescence images (TUNEL assay, γ-H2AX and Ki67) from tumor tissue sections slices (scale bar = 100 μm). Immunofluorescence experiments were independently repeated three times. **f** Representative immunofluorescence images of tumor sections stained with DAPI (blue), CD31 (red), α-SMA (yellow), and HIF-1α (green) antibodies (scale bar = 200 μm, and scale bar = 50 μm in magnified images). Immunofluorescence experiments were independently repeated three times. **g** Blood chemistry parameters in mice after different treatments (n = 3 independent replicates), including white blood cell (WBC), red blood cell (RBC), platelet (PLT), alanine aminotransferase (ALT), aspartate aminotransferase (AST), and creatinine (CREA). **h–n** Representative flow cytometric analysis and relative quantification of CD4^+^ T cells and CD8^+^ T cells, CD8^+^CD69^+^T cells, and DCs in the tumor. **o** Representative immunofluorescence images of lymph node sections stained with DAPI (blue), CD8 (red), and CD11c (green) antibodies (scale bar = 500 μm, and scale bar = 100 μm in magnified images). Immunofluorescence experiments were independently repeated three times. **p** Schematic illustration of the interaction between DCs and CD8^+^T cells. Results are presented as mean ± S.D. Statistical significance was determined using one-way ANOVA for multiple-group comparisons, followed by Tukey’s two-tailed post hoc test for pairwise analysis. (Color figure online)

Encouraged by the superior performance of IMM-augmented RT in vitro, we investigated its antitumor efficacy in a colon carcinoma mouse model of CT26 tumor-bearing BABL/c mice (Fig. 5a). When the tumors reached 80–100 mm^3^ in volume on day 7 post-tumor inoculation, MM or IMM was injected intratumorally, followed by a single fraction of X-ray irradiation at 6 Gy. At 12 days post-treatment, all mice were sacrificed, and tumors were excised for imaging and weighing (Fig. S23). Although IMM or RT alone partially delays tumor growth compared with the control group, the combination of RT and IMM shows stronger tumor growth inhibition, highlighting IMM’s radiosensitization effect (Figs. 5b–d and S22). Meanwhile, stable body weight and histological analysis of major organs indicate the favorable biosafety profile of IMM (Fig. S23).

Furthermore, H&E histological staining reveals severe necrosis in tumor slices following IMM-augmented RT treatment (Figs. 5e and S24). The TdT-mediated dUTP nick-end labeling (TUNEL) assay, γ-H2AX detection, and Ki67 detection were employed to assess the antitumor efficacy of IMM-augmented RT. Compared with the control, RT, and IMM groups, fluorescence images of the IMM-augmented RT treatment group demonstrate stronger green fluorescence in the TUNEL assay, reduced Ki67 expression, and increased γ-H2AX expression (Fig. 5e). These results robustly support the heightened antitumor efficacy achieved through IMM-augmented RT treatment. Specifically, the synergistic effect of IMM not only effectively intensifies DNA damage in tumor cells but also inhibits their proliferation and promotes apoptosis. Moreover, we examined the expression of three markers (CD31, α-SMA, and HIF-1α) in tumor slices to assess the influence of IMM-augmented RT on tumor tissue hypoxia and intratumoral angiogenesis (Fig. 5f). In the IMM-augmented RT treatment group, results reveal a decrease in CD31-marked blood vessels, accompanied by a heightened expression of α-SMA in the vascular region. This finding suggests that IMM-augmented RT treatment promotes the normalization of tumor blood vessels. This normalization reduces the risk of tumor metastasis and improves the delivery of O_2_ and therapeutic drugs to the tumor [6]. Notably, the expression of HIF-1α in the IMM-augmented RT group exhibits a marked decrease, underscoring the alleviation of tissue hypoxia after IMM + RT treatment. Blood chemistry analysis further confirms that all evaluated parameters remain within normal reference ranges, indicating a favorable biosafety profile of IMM therapy (Figs. 5g and S25).

Flow cytometry further confirms the robust antitumor immune response induced by IMM-augmented RT (Fig. 5h–n). Flow cytometry analysis revealed no statistically significant differences in the proportions of CD4^+^ or CD8^+^ T cells among the four treatment groups. Nonetheless, the observed trend in CD8^+^ T cell dynamics across groups may hold potential biological relevance. Previous studies have shown that local therapies such as RT, while inducing ICD of tumor cells and activating immune responses, may also cause nonspecific damage to preexisting immune cells within the TME. When such damage exceeds the immune activation effect, local immune responses may be attenuated [74–76]. This may explain the relatively lower proportion of CD8^+^ T cells observed in the RT group. Importantly, the proportion of CD8⁺ T cells was higher in the RT + IMM group compared to RT alone, suggesting that IMM may enhance systemic immune activation, thereby promoting the recruitment and re-infiltration of effector immune cells, including CD8^+^ T cells, partially compensating for the local immune cell depletion caused by RT. Further analysis revealed a significant increase in the CD8^+^CD69^+^-activated T cell subset following RT + IMM treatment. This subset likely possesses stronger cytotoxic activity, contributing to the synergistic enhancement of antitumor immune responses [77]. Additionally, the increased abundance of dendritic cells (DCs) in the RT + IMM group suggests enhanced tumor antigen presentation, thereby facilitating the initiation of tumor-specific immune responses [78, 79].

Immunofluorescence analysis was conducted on tissue slices from tumor-draining lymph nodes (Fig. 5o). The results show that IMM-augmented RT treatment increased the proliferation of DCs (green fluorescence) and CD8^+^ T cells (red fluorescence) in lymph node tissues. This suggests that the immunomodulatory effects of IMM in combination with RT extend beyond the tumor site, propagating through lymph nodes and triggering systemic immune activation. Flow cytometric analysis further reveals a pronounced increase in the proportion of CD8⁺PD-1⁺ T cells following IMM-augmented RT. Compared with RT alone, RT + IMM treatment significantly elevates PD-1 expression on CD8⁺ T cells (*p* = 0.0006), whereas no significant difference is observed between the IMM and RT + IMM groups, indicating that IMM is the primary driver of PD-1 induction. This PD-1 upregulation, occurring in the context of enhanced immune activation, suggests the emergence of an immune checkpoint-exposed state and provides a strong rationale for subsequent combination with anti-PD-1 therapy (Fig. S26).

**Fig. 6:**
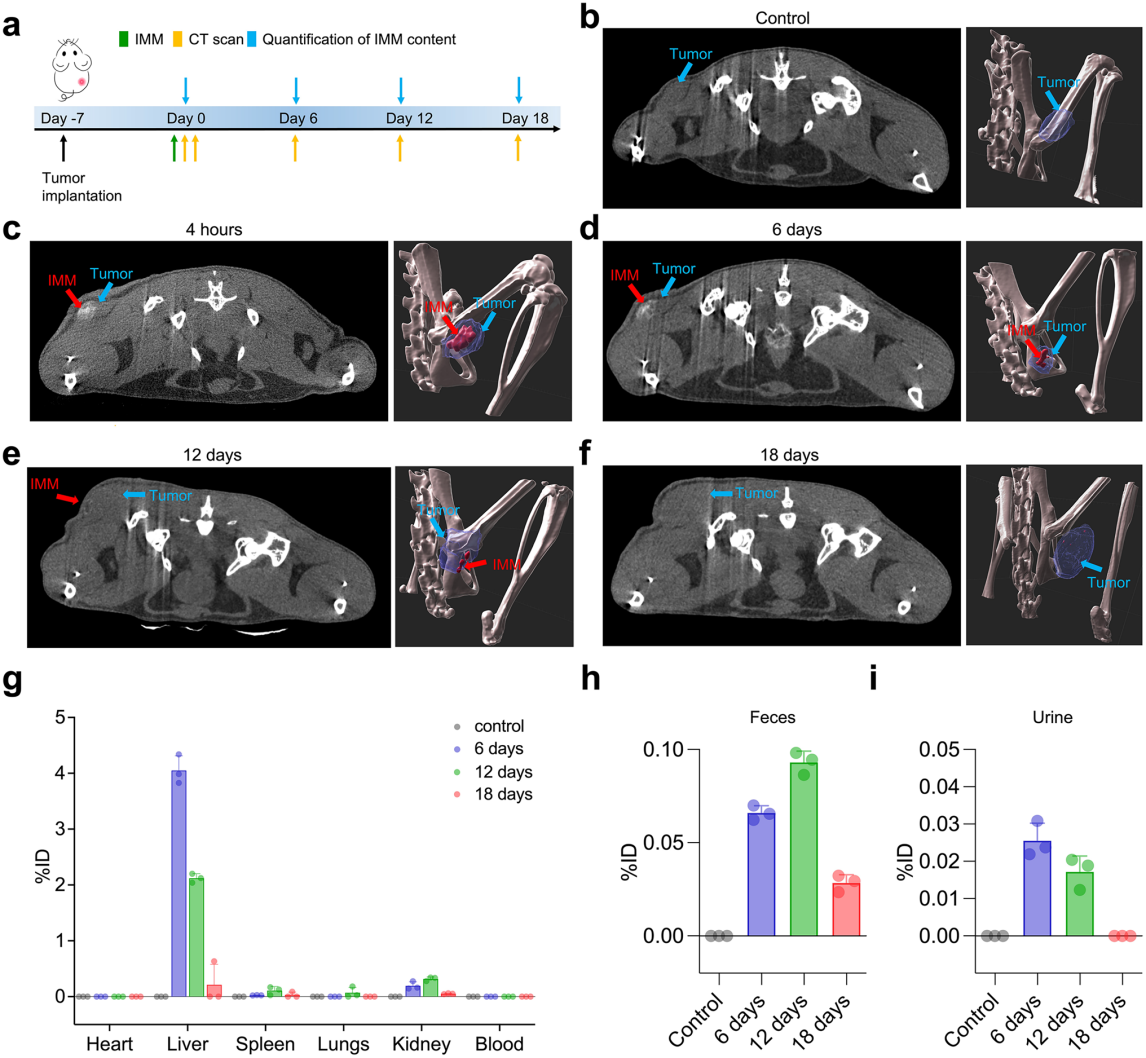
In vivo fate of IMM. **a** Schematic illustration of dynamic monitoring of IMM in vivo distribution via CT scans and ICP-MS analysis. **b–f** Representative reconstruction images from multi-time-point CT scans (including control, 4 h, 6 days, 12 days, and 18 days after injection) showing skeletal structure, IMM (red), and tumor (blue). Experiments were independently repeated three times. **g–i** Biodistribution of IMM in major organs, blood, and metabolic excreta of mice at different time points following intratumoral injection (n = 3 independent replicates). In **b** and **g–i**, the control group indicates saline. Results are presented as mean ± S.D. (Color figure online)

Furthermore, the in vivo biodistribution profile and metabolic fate of IMM were systematically investigated (Fig. 6a). CT-based three-dimensional reconstruction at five time points reveals the distribution and retention dynamics of IMM within tumor tissue (Fig. 6b–f). The results show that IMM remains localized at the tumor site after injection, maintaining a stable distribution that enhances the effectiveness of subsequent RT. Meanwhile, the results demonstrate a gradual, time-dependent decline in intratumoral IMM levels. This clearance ensures that the drug remains at therapeutic concentrations during the treatment window while minimizing long-term accumulation and reducing the risk of potential toxicity.

To elucidate the clearance pathway of IMM, we quantitatively analyzed iridium levels in major organs (heart, liver, spleen, lungs, and kidneys), blood, and excretions using inductively coupled plasma mass spectrometry (ICP-MS). Results reveal that at all observed time points, the percentage of injected dose (%ID) of iridium in the liver is higher than that in other organs (Fig. 6g). Additionally, fecal and urinary excretion profiles indicate that IMM is eliminated through the hepatobiliary system (predominant route) and renal pathways (Fig. 6h, i). Notably, iridium accumulation remains minimal in other major organs (heart, spleen, and lungs) and in peripheral blood throughout the study, providing evidence for the systemic safety profile of IMM.

### IMM-Augmented RT Reprograms Tumor TME

**Fig. 7:**
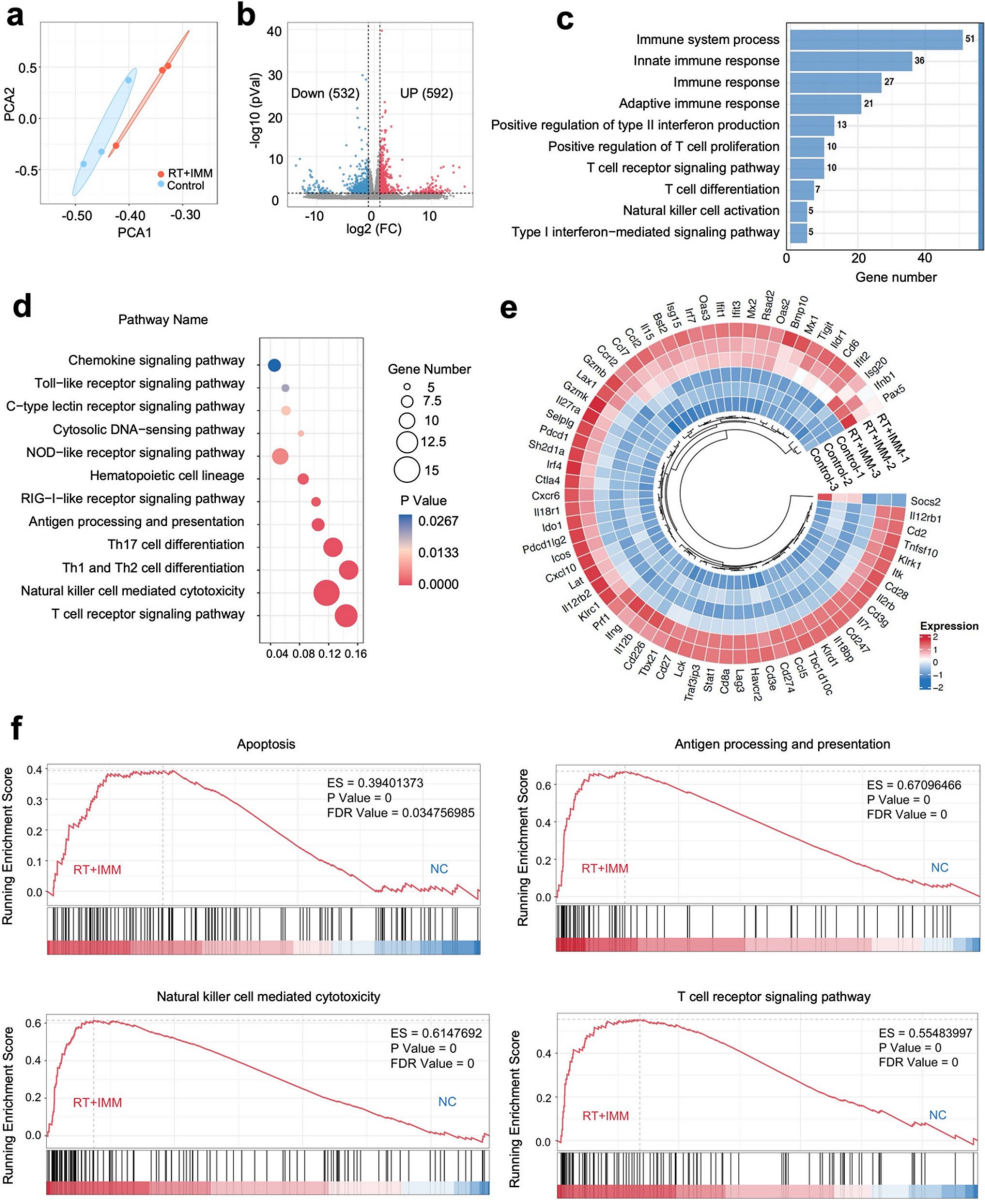
IMM-augmented RT reprograms TME. **a** PCA and **b** Volcano plot of differential expression genes in RNAseq between control and RT + IMM-treated tumors 12 days post-treatment (n = 3 independent replicates). **c** GO and **d** KEGG enrichment analyses of differentially expressed genes (n = 3 independent replicates). **e** Expression of immune-related genes in the control and RT + IMM groups. **f** GSEA of altered gene sets in the RT + IMM group compared with the control group

Transcriptomic profiling of control and RT + IMM tumors was used to define the molecular basis of tumor suppression mediated by IMM-augmented RT. The principal component analysis (PCA) demonstrates the differences between samples from the control and RT + IMM groups, indicating an alteration in the overall gene expression within tumor tissue following RT + IMM treatment (Fig. 7a). Transcriptomic analysis identifies a total of 1,124 differentially expressed genes, including 592 upregulated and 532 downregulated genes (Fig. 7b). Both Gene Ontology (GO) and Kyoto Encyclopedia of Genes and Genomes (KEGG) analysis reveal that the differentially expressed genes are significantly enriched in the immune-related pathways (Fig. 7c, d). Figure 7e presents the expression of immune-related genes in the control and RT + IMM groups [80]. In the RT + IMM group, immune activation-related genes are significantly upregulated, except for the suppressor of cytokine signaling 2 (SOCS2), an immune suppression-associated gene, which shows a downregulation trend. The SOCS protein family is a crucial regulator of cytokine signaling, playing a vital role in maintaining the anti-inflammatory phenotype of iTreg by inhibiting the secretion of proinflammatory cytokines [81, 82]. Consistent with the observed decrease in SOCS2 expression, serum cytokine analysis reveals significantly elevated levels of TNF-α, IL-6, IFN-γ, and IFN-β after RT + IMM treatment, indicating a shift toward a proinflammatory and immunostimulatory milieu (Fig. S27). Together, these findings suggest that RT + IMM treatment not only enhances immune activation at the transcriptional level but also increases secretion of key proinflammatory cytokines, supporting the induction of a more robust antitumor immune response. Moreover, gene set enrichment analysis (GSEA) comparing the RT + IMM group with the control group reveals that gene sets in apoptosis, antigen processing and presentation, natural killer cell-mediated cytotoxicity, and T cell receptor signaling are significantly upregulated (Fig. 7f).

### Abscopal Effect of IMM-Augmented RT + anti-PD-1 Synergy Therapy

The abscopal effect was first proposed by Mole in 1953 and has subsequently been confirmed to exist in various types of tumors [83, 84]. The abscopal effect is regarded as a key indicator of how RT’s local effect can stimulate a broader, systemic antitumor immune response [85, 86]. The current understanding indicates that the primary mechanisms of the abscopal effect include the release of tumor-associated antigens (TAAs), DAMPs, and cytokines, which activate CD8^+^ T cells, thereby promoting the elimination of tumor cells [87, 88]. The aforementioned results demonstrate that IMM-augmented RT achieves significant antitumor effects in vivo and upregulates PD-1 expression. This observation provides strong evidence for the combined use of IMM-augmented RT and anti-PD-1 therapy to synergistically enhance therapeutic efficacy, suggesting that the addition of anti-PD-1 treatment can effectively alleviate tumor immune evasion by reactivating exhausted T cells and strengthening antitumor immune responses. This combinatorial strategy is expected to promote immune cell infiltration and further activation within the TME, thereby facilitating systemic tumor eradication [89].

**Fig. 8:**
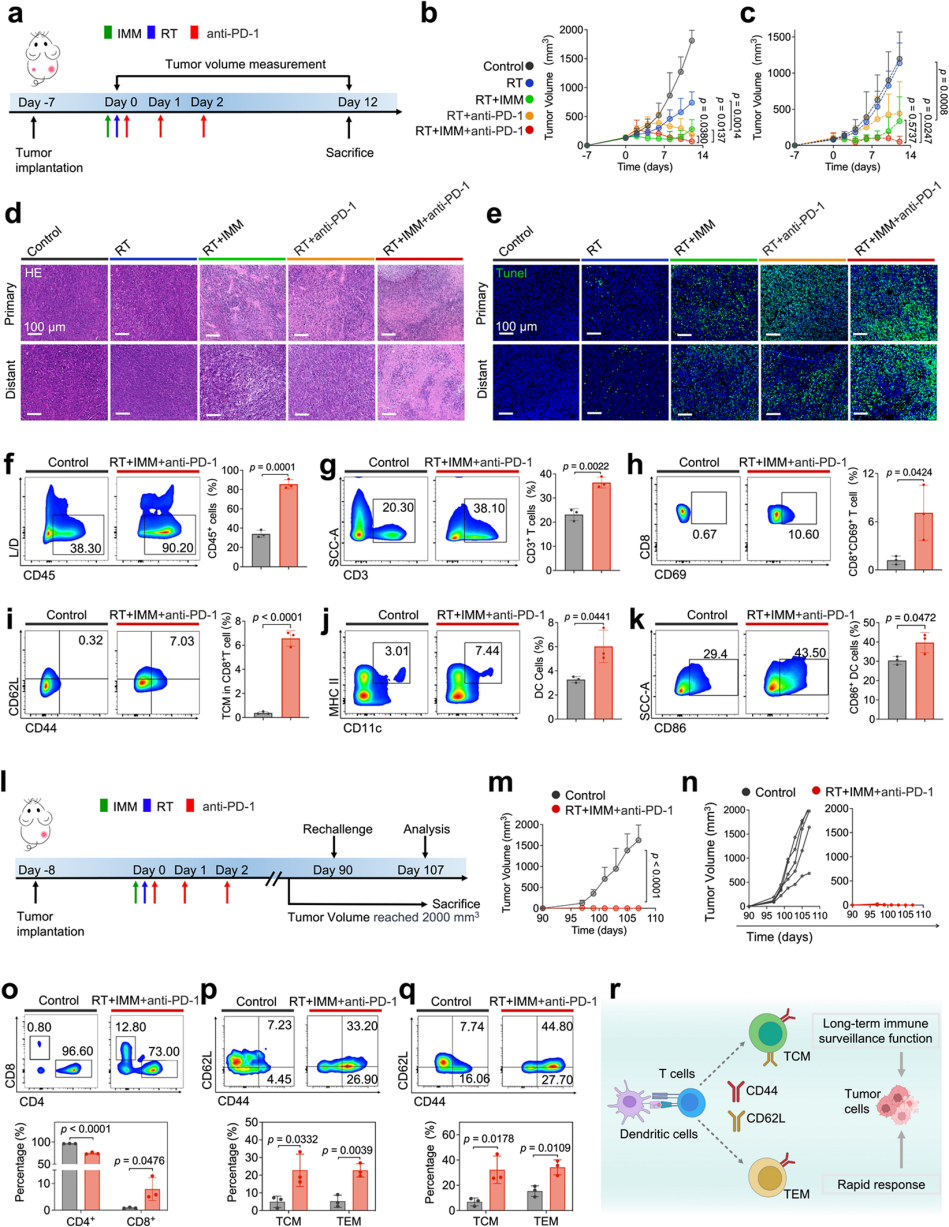
IMM enhances the abscopal effect of RT combined with anti-PD-1 treatment, leading to the formation of a strong antitumor memory. **a** Schematic illustration of IMM-augmented RT combined with anti-PD-1 treatment (n = 5 biologically independent mice per group). **b** Average tumor growth curves of primary tumor. **c** Average tumor growth curves of distant tumor. **d** Representative H&E and **e** immunofluorescence images of TUNEL assay from tumor tissue sections (scale bar = 100 μm). Experiments were independently repeated three times. **f**–**k** Representative flow cytometric analysis and quantification of CD45^+^ T cells and CD3^+^ T cells, CD8^+^CD69^+^T cells, TCM, and DCs in the spleen. **l** Schematic illustration of the experimental design to evaluate the antitumor memory response induced by IMM-augmented RT combined with anti-PD-1 therapy. (n = 5 biologically independent mice per group). **m** Average tumor growth curves and **n** individual tumor growth curves of the treated mice. **o**–**q** Representative flow cytometric analysis of control and IMM-augmented RT plus anti-PD-1 group, with corresponding quantification of CD4^+^, CD8^+^ from CD3^+^ T cells, and subsets central memory T cells (Tcms, CD62L^+^CD44^+^) and Tems (CD62L^−^CD44^+^) from CD8^+^ and CD4^+^ T cells in the spleen. **r** Schematic illustration of the formation of Tcms and Tems. Results are presented as mean ± S.D. Statistical significance was determined using the Student’s t test for two-group comparisons, and one-way ANOVA for multiple-group comparisons, followed by Tukey’s two-tailed post hoc test for pairwise analysis

Intentionally, a model of mice bearing bilateral CT26 tumors was employed (Fig. 8a). Primary tumors were established on the right flank (5 × 10^5^ CT26 cells), while secondary tumors were implanted on the left flank (5 × 10^4^ CT26 cells) to mimic distant tumor lesions. The treatment for the primary tumors remained the same as Fig. 5a, with the additional intraperitoneal injection of anti-PD-1. The initial dose of anti-PD-1 was 20 mg kg^−1^, followed by two subsequent treatments, each with a dose of 10 mg kg^−1^. Significantly, the results reveal that IMM-augmented RT combined with anti-PD-1 not only effectively suppresses the primary tumor but also markedly inhibits the growth of distant tumors. While the RT group, RT + IMM, and RT + anti-PD-1 group all exhibit partial inhibitory effects on the primary tumors, no discernible impact on distant tumor growth is observed (Figs. 8b, c and S28). H&E staining and fluorescence images further confirm the above results (Fig. 8d, e). In the RT + IMM combined with the anti-PD-1 group, H&E histological staining reveals significant necrosis in both primary and distant tumors.

Additionally, TUNEL fluorescence images show increased apoptosis in tumor cells. The weight of mice across all groups remained stable, and histological examination of major organs collected from each group revealed no notable abnormalities, suggesting the biosafety of the RT + IMM combined with anti-PD-1 treatment (Fig. S29). Subsequently, we analyzed the cellular component in the spleens of mice from both the control group and the RT + IMM combined with anti-PD-1 treatment group (Fig. 8f–k). The results demonstrate that RT + IMM combined with treatment significantly activates the immune system, leading to a notable proliferation of CD45^+^ cells, CD3^+^ T cells, and DCs. Additionally, there is a significant increase in CD8^+^CD69^+^ T cells, Tcms, and activated DCs (CD86^+^ DCs). These findings suggest that RT + IMM, combined with anti-PD-1 treatment, effectively promotes the activation and proliferation of immune cells, thereby achieving systemic tumor elimination.

Antitumor memory, a key element of the adaptive immune system, manifests a swifter and more efficient response upon repeated exposure to antigens, ensuring long-term protection [90]. To assess whether RT + IMM combined with anti-PD-1 therapy could elicit functional antitumor immune memory, a tumor rechallenge experiment was performed using mice that met the predefined criteria for immune memory evaluation following the initial treatment regimen (Fig. 8l). Briefly, CT26 cells (5 × 10^5^) were inoculated into the right flank of BALB/c mice, followed by RT + IMM in combination with anti-PD-1 treatment as described in Fig. 8a. Age- and sex-matched naïve mice were included as controls. On day 90, a tumor rechallenge was performed by inoculating 5 × 10^5^ CT26 cells into the left flank of both tumor-free mice (with complete tumor regression) and control mice. Notably, no noticeable tumor growth is observed in the mice of RT + IMM combined with the anti-PD-1 treatment group after the rechallenge, demonstrating a significant antitumor memory effect generated by the RT + IMM combined with anti-PD-1 treatment. In contrast, rapid tumor progression is observed in all naive mice in the control group after the rechallenge (Fig. 8m, n).

To further validate the underlying mechanism, spleens from mice 10 days after tumor rechallenge were collected to assess changes in memory T cells (Fig. 8o–q). The populations of Tcms and Tems show clear differences in both their homing capacity and effector functions [91]. Tcms (CD62L^+^CD44^+^) provide long-term protection and antitumor memory, while Tems (CD62L^−^CD44^+^) can rapidly mount effector responses upon re-exposure to antigens (Fig. 8r). This difference enables them to collaborate and collectively maintain the integrity of the defense system. It is noteworthy that in the RT + IMM combined with anti-PD-1 group, flow cytometry analysis showed a significant increase in CD8^+^ T cells compared with the control group. This implies that more CD8^+^ T cells are activated, exerting a systemic antitumor effect. Additionally, it is encouraging that a substantial increase in both Tems and Tcms is observed in CD4^+^ and CD8^+^ cells in the RT + IMM group combined with anti-PD-1 treatment, providing crucial evidence of an established antitumor response. These findings indicate that RT + IMM combined with anti-PD-1 treatment can activate a sustained proinflammatory response, thus potentially preventing tumor recurrence.

### Exploring the Efficacy of IMM-Augmented RT in Humanized PDX Models and Spontaneous Lung Metastasis Breast Cancer Animal Model

**Fig. 9:**
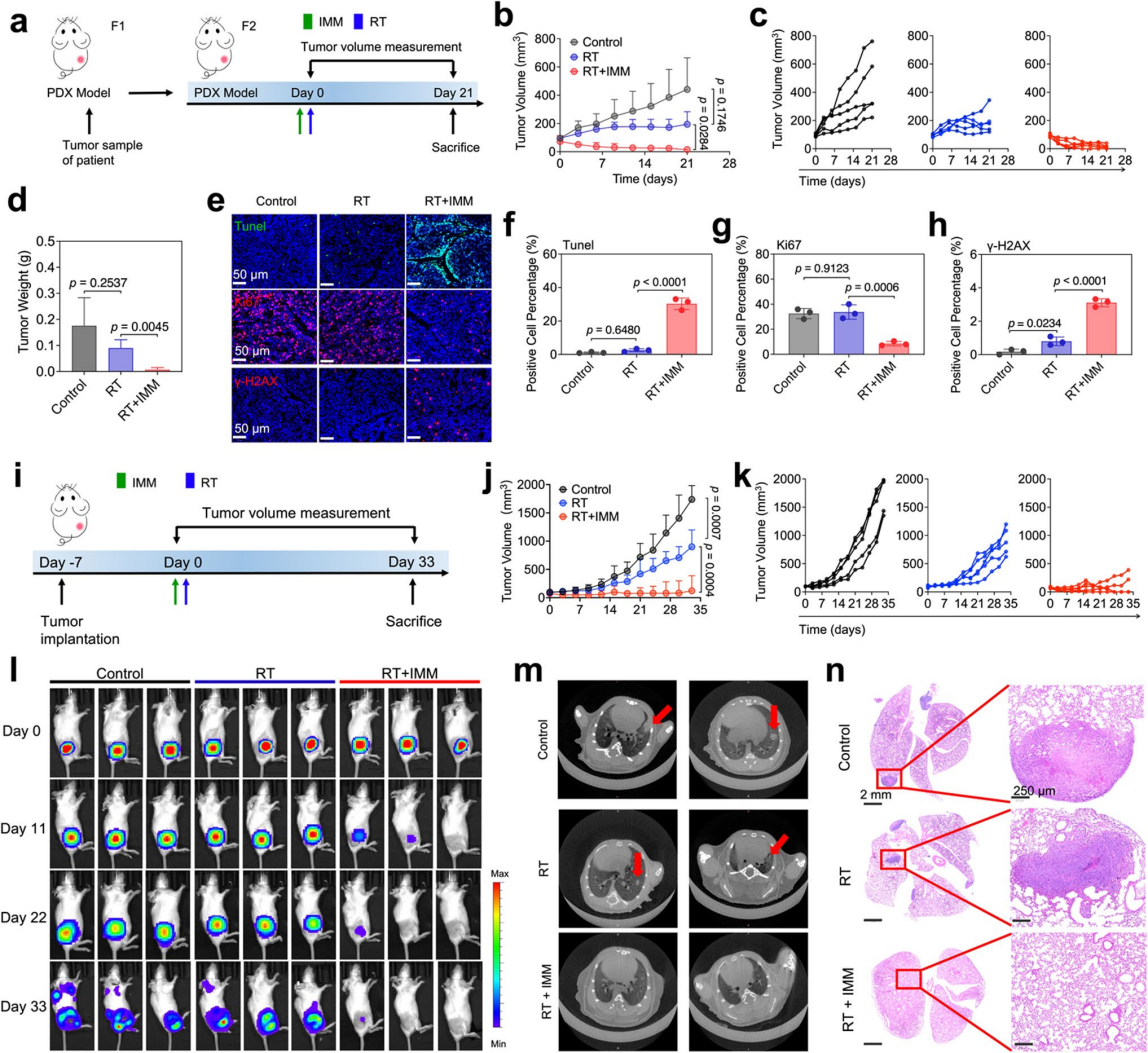
Exploring the efficacy of IMM-augmented RT in humanized PDX models and spontaneous lung metastasis breast cancer animal model (4T1). **a** Schematic illustration of IMM-augmented RT for humanized PDX models. **b** The average tumor growth curves of tumor, **c** individual tumor growth curves of tumor, and **d** tumor weight of excised tumors (n = 5 biologically independent mice per group). **e**–**h** Representative immunofluorescence images of tumor sections stained for TUNEL, γ-H2AX, and Ki67 (scale bar = 50 μm), together with bar graphs showing the percentage of positive cells. Experiments were independently repeated three times. **i** Schematic illustration of IMM-augmented RT in spontaneous lung metastasis breast cancer animal model (n = 5 biologically independent mice per group). **j** The average tumor growth curves of the tumor and **k** individual tumor growth curves of the tumor. **l** Representative bioluminescence images of luciferase-expressing 4T1 tumor from each group after varied treatments. Experiments were independently repeated three times. **m** Representative CT scans of mouse lungs from various treatment groups, with red arrows indicating lung metastases. Experiments were independently repeated three times. **n** Representative H&E images from lung tissue sections slices (scale bar = 2 mm, and scale bar = 250 μm in magnified images). Experiments were independently repeated three times. Results are presented as mean ± S.D. Statistical significance was determined using the one-way ANOVA for multiple-group comparisons, followed by Tukey’s two-tailed post hoc test for pairwise analysis. (Color figure online)

To further validate the clinical potential of IMM-augmented RT and its broad applicability to other tumor types, we subsequently evaluated the therapeutic effects of IMM-augmented RT in humanized PDX models from radioresistant human tumors and in a subcutaneous breast cancer mouse model with spontaneous lung metastasis. Tumor samples removed from patients with recurrent head and neck tumors after RT were used to construct a radioresistant PDX model, and the IMM-augmented RT treatment as described in Fig. 9a. Compared to the control group, IMM-augmented RT significantly suppresses tumor growth, while the RT-only group shows limited therapeutic effects (Fig. 9b–d). These results demonstrate that IMM substantially enhances the radiosensitivity of radioresistant tumors, overcoming inherent resistance and markedly improving overall therapeutic outcomes. This finding highlights IMM-augmented RT as a promising strategy for addressing radioresistance in cancer treatment. In addition, H&E histological staining shows that after IMM-augmented RT, the necrotic areas in tumor sections increased (Fig. S30). Compared with the control group and the RT-alone group, the IMM-augmented RT group shows stronger green fluorescence in TUNEL assay images, indicating an increase in apoptotic cells (Fig. 9e, f). Meanwhile, tumor sections from the IMM-augmented RT group also show decreased expression of Ki67 (Fig. 9e, g) and increased expression of γ-H2AX (Fig. 9e, h). Overall, the encouraging results of IMM-augmented RT in PDX models demonstrate the potential of this treatment strategy in reversing RT resistance and highlight its promising clinical applicability. These findings further strengthen our confidence in the broad prospects of IMM-augmented RT for cancer treatment.

Furthermore, in the mouse breast cancer model, the IMM-augmented RT strategy exhibits marked tumor suppression and effectively inhibits metastasis (Fig. 9i). The IMM-augmented RT triggers a strong antitumor response that is maintained for 30 days post-treatment (Fig. 9j, k). H&E staining and fluorescence analysis of tumor tissues show elevated rates of tumor cell apoptosis, decreased 4T1 cell proliferation, and enhanced DNA damage following IMM-augmented RT treatment (Fig. S31). The consistent antitumor activity of IMM-augmented RT was further confirmed by bioluminescence imaging, which shows that mice in the IMM-augmented RT group achieve complete tumor regression (Fig. 9l). Moreover, CT scans and H&E-stained lung tissue sections reveal that mice treated with IMM-augmented RT show no evidence of lung metastasis, whereas metastatic lesions are present in both the control and RT groups, underscoring the superior efficacy of the combined treatment in preventing metastatic spread (Fig. 9m, n).

## Conclusion

In summary, we report the de novo design of the biocatalytic and radiosensitizing artificial metalloenzymes with an IrMn-cluster-based redox center to eradicate primary and metastatic tumors. Our studies demonstrate that the IMM exhibits efficient electron transfer and enriched Ir catalytic centers, enabling it to produce massive ROS, inhibit DNA repair, and trigger robust apoptosis. Meanwhile, O_2_ generation alleviates tumor hypoxia, thereby enhancing the sensitivity of cancer cells to X-ray irradiation. Accordingly, this biocompatible and biodegradable metal–organic complex improves radiosensitivity and remodels the TME, resulting in a potent antitumor effect. RT followed by IMM provokes robust ICD in tumor cells, leading to the release of DAMPs that recruit and activate effector immune cells at the tumor site. Remarkably, in the presence of anti-PD-1 therapy, IMM amplifies systemic antitumor response, enabling the suppression of both local and distal tumor lesions while mitigating the risk of recurrence. Furthermore, the therapeutic advantages of IMM have been validated in humanized PDX models derived from radioresistant patients, as well as in a spontaneous lung metastasis breast cancer animal model, thereby reinforcing the broad clinical applicability of this strategy. Such fundamental insights will catalyze the evolution of these therapeutics from bench to bedside, ultimately enabling a paradigm shift in precision oncology through seamless integration of physicochemical and biological principles.

## Supplementary Information

Below is the link to the electronic supplementary material.

Supplementary file1 (DOCX 36187 kb)
