# Supplementary material for: IrMn-Cluster-Based Artificial Metalloenzymes with Radiosensitized Systemic Antitumor Responses to Prevent Malignant Tumor Metastasis and Recurrence
Source: Nanomicro Lett. 2026 Jul 27;19:4. doi: 10.1007/s40820-026-02309-2 (PMC13407826; doi:10.1007/s40820-026-02309-2)
Supplement: Supplementary file 1 — Supplementary file1 (DOCX 36187 kb) [file 40820_2026_2309_MOESM1_ESM.docx]

Supporting Information for

**IrMn-Cluster-Based Artificial Metalloenzymes with Radiosensitized Systemic Antitumor Responses to Prevent Malignant Tumor Metastasis and Recurrence**

Ruidan Li^1,2,#^, Qinlong Wen^1,#^, Zhenyu Xing^3^, Ting Wang^3^, Jing Yang^1^, Yunfeng Tao^1^, Shengdong Mu^4^, Shuang Li^3^, Zhigong Wei^1,6^, Chong Cheng^3,5,^*, and Xingchen Peng^1,6,^*

^1^ Department of Biotherapy, Cancer Center, West China Hospital, Sichuan University, Chengdu 610041, P. R. China

^2^ West China School of Public Health and Department of Oncology, West China Fourth Hospital, Sichuan University, Chengdu 610041, P. R. China

^3^ College of Polymer Science and Engineering, State Key Laboratory of Advanced Polymer Materials, Sichuan University, Chengdu 610065, P. R. China

^4^ College of Biological Engineering, Sichuan University of Science and Engineering, 188 University Town, Yibin, P. R. China

^5^ Department of Endodontics, State Key Laboratory of Oral Diseases, National Clinical Research Center for Oral Diseases, West China Hospital of Stomatology, Sichuan University, Chengdu 610041, P. R. China

^6^ Nuclear Physics and Medical Research Key Laboratory of Sichuan Province, Sichuan University, Chengdu 610041, P.R. China

*^#^* Ruidan Li and Qinlong Wen contributed equally to this work.

* Corresponding authors. E-mail: [chong.cheng@scu.edu.cn](mailto:chong.cheng@scu.edu.cn) (Chong Cheng); [pxx2014@scu.edu.cn](mailto:pxx2014@scu.edu.cn) (Xingchen Peng)

**S1 Supplementary Text**

***S1.1 Characterization.***

Scanning electron microscope (SEM) was conducted with Apreo S HiVac-ThermoFisherScientific (FEI). A Nicolet-560 spectrophotometer (Nicol, US) was used for Fourier transform infrared (FTIR) spectroscopy analysis of the samples in the range of 4000-500 cm^-1^. The surface elemental composition and all binding energies were measured by X-ray photoelectron spectroscopy (XPS, Thermo ESCALAB 250Xi) with Al Kα monochromatic X-ray sources, and data were analyzed via the Thermo Scientific Avantage software to confirm the surface chemical structures and metal contents. Raman spectra were acquired on XploRA PLUS (HORIBA). X-ray absorption spectra (XAS) were collected on the beamline BL07A1 in NSRRC (National Center for Synchrotron Radiation Research). The radiation was monochromatized by scanning a Si (111) double-crystal monochromator. Catalysts were pressed into thin slices and positioned at 90° to the incident beam in the sample holder. X-ray absorption near-edge structure (XANES) and extended X-ray absorption fine structure spectra (EXAFS) data reduction and analysis were processed by the Athena software. The X-ray diffraction (XRD) patterns were used to analyze the crystal structures of the catalysts via a Bruker D8 Focus X-ray diffractometer with Cu Kα radiation for a 2θ range of 10-80°. The aberration-corrected high-angle annular dark-field scanning TEM (AC HAADF-STEM) was performed on a JEOL JEM-ARM 200F scanning transmission electron microscope equipped with a 5 cold field emission electron source and a DCOR probe corrector (CEOS GmbH), a 100 mm^2^ JEOL Centurio EDX detector. Electron paramagnetic resonance (EPR) measurements were performed *via* the Bruker EPR EMX Plus.

***S1.2 Peroxidase (POD)-mimetic activity measured by 3,3',5,5'-tetramethylbenzidine (TMB) assay.***

The material solution (10 mg/mL, 5 μL) was added to a NaOAc-HOAc buffer (100 mM, pH 4.5). Then, 24 μL of TMB (10 mg/mL) and 25 μL of H_2_O_2_ (0.1 M) were added, respectively. The final volume of the mixture was 2 mL. Then, a portion of the mixture was used for UV-visible spectroscopy measurements at an absorbance of 652 nm.

***S1.3 Steady-state enzyme kinetic studies.***

The Michaelis-Menten constant was calculated based on the Michaelis-Menten saturation curve. For each H_2_O_2_, the initial reaction rates (*V*_0_) were calculated from the absorbance variation using the Beer-Lambert Law (Equation (S1) (with an ε of 39,000 M^−1^ cm^−1^ for oxTMB, where c indicates the oxTMB concentration and l cm for the length of the solution in the light path). The reaction rates were then plotted against their corresponding H_2_O_2_ and then fitted with the Michaelis–Menten curves. Furthermore, a linear double-reciprocal plot (Lineweaver–Burk plot, Equation (S2)) was used to determine the maximum reaction velocity (*V*_max_) and Michaelis constant (*K*_m_). Furthermore, the turnover number (TON) was calculated according to Equation (S3).

$V_{0}=\frac{V_{\max}\cdot\left[ S \right]}{K_{m}+\left[ S \right]}$ (S1)

$\frac{1}{V_{0}}=\frac{K_{m}}{V_{\max}}\cdot\frac{1}{\left[ S \right]}+\frac{1}{V_{\max}}$ (S2)

$TON=\frac{V_{\max}}{\left[ E_{0} \right]}$ (S3)

[S] is the concentration of H_2_O_2_, and [E_0_] is the molar concentration of metal in nanozymes.

***S1.4 Detection of superoxide anions (•O- 2) by dihydroethidium (HE) probe.***

HE was a specific probe that could react with 🞄O_2_^-^ to produce fluorescent ethidium. First, 1.5 mL of 100 μg·mL^-1^ of materials-buffer solution (1.0 M acetate buffer (pH 4.5)) was mixed with 1.5 μL of 0.1 M H_2_O_2_ at 37 ℃ for 40 min. Then, 1.5 mL of HE-ethanol solution (1 mg/mL) was added to the system. Afterward, the solution was vortexed and left undisturbed for 40 min before fluorescence measurement.

***S1.5 Detection of singlet oxygen (^1^O_2_) by 9,10-diphenanthraquinone (DPA).***

The 50 μg/mL material buffer solution (1.0 M acetate buffer, pH 4.5) was mixed with 1 μg/mL DPA at a volume ratio of 1: 40, followed by the analysis using a UV-vis spectrophotometer. The relative absorbance changes of DPA at 378 nm were used to quantify the decomposition rate.

***S1.6 Catalase-like (CAT-like) Activity Assay.***

O_2_ generation assay. A total of 100 mM of H_2_O_2_ and 10 μg/mL of materials were mixed in PBS (pH = 6.5) to 20 mL, followed by measuring the O_2_ concentration using a dissolved oxygen meter (INESA, JPSJ-605F) every 5 s until 300 s.

***S1.7 EPR measurement.***

The generation of ^1^O_2_ was evaluated by an EPR spectrometer using TEMP spin-trapping adduct in NaOAc-HOAc buffer (100 mM, pH 4.5). 10 μL of biocatalysts (10 mg/mL, NaOAc-HOAc buffer) and 10 μL of H_2_O_2_ (10 M) were added into a 0.5 mL buffer, and then 10 μL TEMP was added.

The generation of •O_2_^-^ was evaluated by an EPR spectrometer using DMPO spin-trapping adduct in DMSO solvent. 10 μL of biocatalysts (10 mg/mL, DMSO) and 10 μL of H_2_O_2_ (10 M) were added to a 0.5 mL DMSO, and then 10 μL DMPO was added.

***S1.8 X-ray-activated enzyme-like activity.***

A stock solution of o-phenylenediamine (OPD) was prepared by dissolving 10 mg of OPD in 1 mL of DMF. For the catalytic reaction, 10 μL of H_2_O_2_ (0.1 M), 24 μL of the OPD stock solution, and 20 μL of each material suspension (4 mg/mL) were sequentially introduced into 1925 μL of NaOAc/HOAc buffer (100 mM, pH 5.5). An aliquot of 200 μL from the mixture was dispensed into a 96-well plate. The samples were then exposed to 6 Gy of X-ray irradiation, after which the maximum absorption wavelength at 448 nm was recorded.

***S1.9 In-situ FTIR measurement.***

In-situ FTIR measurements were performed on an infrared spectrometer (Nicolet iS50, Thermo Fisher Scientific Inc., USA) equipped with a liquid nitrogen-cooled MCT detector. The catalyst was filled into an in situ infrared holder in the chamber. Before the measurement, the chamber was degassed under argon flow at a rate of 6 L⋅min^-1^ for 10 min. Then, NaOAc-HOAc buffer and H_2_O_2_ were added to the reactor with the sample, and the reaction was recorded in 10 min.

***S1.10 Density functional theory (DFT) calculation.***

All theoretical calculations were performed using the DFT method, as implemented in the Vienna ab initio simulation package (VASP) [S1–S3]. The core electrons were described using the spin-polarized projector augmented wave (PAW) method [S4], and the electron exchange and correlation energy was treated within the generalized gradient approximation in the Perdew-Burke-Ernzerhof functional (GGA-PBE) [S5]. The valence states of all atoms were expanded in a plane-wave basis set with a cutoff energy of 500 eV. The convergence criteria for the electronic self-consistent iteration and force were set to 10^-5^ eV and 0.02 eV/Å. Van der Waals (vdW) corrections with zero damping DFT-D3 method of Grimme were used in all structures [S6, S7]. The charge density differences were evaluated using the formula $\text{∆ρ = }\text{ρ}_{\text{A+B}} \text{- }\text{ρ}_{\text{A}} \text{- }\text{ρ}_{\text{B}}$, where $\text{ρ}_{\text{X}}$ is the electron density of X. Atomic charges were computed using the atom-in-molecule (AIM) scheme proposed by Bader [S8, S9].

To explore the catalytic effect, the change of Gibbs free energy (ΔG) was calculated, which is defined as:

$$\text{∆G }\text{= ∆E + ∆ZPE +}\text{ ∆H}_{\text{0→298K}} \text{- T∆S}$$

where ΔE is the energy change obtained from DFT calculations; ΔZPE, ΔH, and ΔS denote the difference in zero-point energy, enthalpy, and entropy due to the reaction, respectively. The enthalpy and entropy of the ideal gas molecule were taken from the standard thermodynamic tables, and some of the calculation results were analyzed by the VASPKIT package [S10].

***S1.11 Cell lines and animals***

The CT26 and 4T1 cell line was originally obtained from the American Type Culture Collection (ATCC), and the SCC7 cells were kindly provided by Dr. Lei Dai (West China Hospital, Sichuan University). Cells were cultured in RPMI 1640 medium (Gibco) containing 1% penicillin (Boster), 1% streptomycin (Boster), and 10% fetal bovine serum (FBS, VivaCell) at 37 °C in 5% CO_2_-humidified air. For subsequent experiments, cells were cultured in hypoxic conditions after seeding in an anaerobic chamber (Thermo Scientific, USA; 94.5% N_2_, 5% CO_2_, and 3% O_2_). Female BALB/c mice (6-8 weeks old) were obtained from Jiangsu Gempharmatech Co., Ltd. and housed in a specific pathogen-free (SPF) facility. The animal experiments were approved by the Institutional Animal Care and Use Committee of West China Hospital, Sichuan University (Approval No. 20241017001).

***S1.12 Apoptosis/necrosis assays.***

CT26 cells were seeded in 6-well plates and incubated overnight. The cells were then treated with PBS, MM, or IMM for 4 hours, followed by irradiation with X-rays at a dose of 6 Gy. After a 48-hour incubation, cell apoptosis was assessed using the Annexin V-FITC/PI apoptosis kit (4A BIOTECH, China) and analyzed by flow cytometry.

***S1.13 Live/Dead assay.***

CT26 cells were seeded in 24-well plates and cultured overnight. The cells were then treated with PBS, MM, or IMM for 4 hours, followed by X-ray irradiation at a dose of 6 Gy. After a 48-hour incubation, cell viability was assessed using the Calcein/PI Live/Dead Viability Assay Kit (Beyotime, China), and images were captured using the Olympus IX83 Live Microscope (Olympus Life Science, Japan).

***S1.14 Clonogenic assay.***

CT26 cells were cultured in 6-well plates overnight and incubated with PBS, MM, or IMM for 4 h, followed by irradiation with 0, 4, 6, and 8 Gy X-ray. Cells were trypsinized and counted immediately. 400–3000 cells were seeded in a 6-well plate and cultured for 8 days. Fix and stain colonies, then manually count the number of colonies.

***S1.15 Cell migration assays.***

Cell migration was assessed using 6.5-mm Transwell chambers with 8.0 μm pores (Labselect, China) made of polyethylene terephthalate. A total of 8 × 10^4^ CT26 cells were pre-incubated with PBS, MM, or IMM, followed by irradiation. After 24 hours of incubation, the non-migratory cells on the upper side of the membrane were gently removed. The migratory cells were then fixed with 4% formaldehyde, stained with 0.1% crystal violet, and counted.

***S1.16 Intracellular reactive oxygen species (ROS) detection.***

ROS production was assessed using the DCFH-DA probe (Beyotime, China). After treatment, CT26 cells were incubated with DCFH-DA (10 μM) for 30 minutes, then imaged with the Olympus IX83 Live Microscope (Olympus Life Science, Japan). For quantitative ROS analysis, cells from different treatment groups were collected, stained with DCFH-DA (10 μM), and analyzed by flow cytometry to measure ROS levels.

***S1.17 The mitochondrial membrane-permeable dye JC-1 staining.***

CT26 cells were cultured overnight in six-well plates and incubated with PBS, MM, or IMM for 4 h, followed by irradiation with X-rays at 6 Gy. After 24 h, the cells were stained with the mitochondrial membrane potential assay kit (JC-1; Beyotime, China) and quantified by flow cytometry in three independent runs for statistical analysis.

***S1.18 γ-H2AX immunofluorescence assay.***

CT26 cells were pre-seeded in a 96-well plate and incubated overnight. The cells were then treated with PBS, MM, or IMM for 4 hours, followed by 6 Gy of X-ray irradiation. After irradiation, γ-H2AX immunofluorescence staining was performed using a DNA damage assay kit (Beyotime, China). The stained cells were visualized and imaged using an Olympus IX83 Live Microscope (Olympus Life Science, Japan).

***S1.19 Detection of immunogenic cell death biomarkers.***

The expression of high mobility group protein box 1 (HMGB1) and calreticulin (CRT) was examined using immunofluorescence assays. CT26 cells, subjected to various treatments, were subsequently cultured under hypoxic conditions for 48 hours. Following fixation with 4% formaldehyde, samples intended for HMGB1 detection underwent an additional 3-minute permeabilization step with 0.5% TritonX-100 (Beyotime, P0096). Subsequently, cells were blocked with 1% bovine serum albumin (BSA) and then incubated overnight at 4 °C with either CRT antibody (ab92516, abcam, 1/500) or HMGB-1 antibody (ab79823, abcam, 1/250), followed by incubation with a secondary antibody for 1 hour. Prior to imaging, cell nuclei were stained with DAPI for ten minutes. Additionally, supernatants were collected from CT26 cell cultures across different experimental groups, and the released ATP content was quantified using an enhanced ATP assay kit (Beyotime, China).

***S1.20 Detection of oxygen generation.***

The oxygen probe [Ru(bpy)_3_]Cl_2_ was used to detect the generated oxygen. CT26 cells subjected to different treatments were incubated with [Ru(bpy)_3_]Cl_2_ for 1 hour and imaged using an Olympus IX83 live-cell microscope (Olympus Life Science, Japan).

***S1.21 Detection of HIF-1α expression.***

Immunofluorescence staining was performed to assess HIF-1α expression. After various treatments, cells were fixed with 4% formaldehyde, permeabilized with Triton X-100, and blocked with 1% BSA. Afterward, cells were incubated overnight at 4 °C with HIF-1α antibody (Ab179483, Abcam, 1/500), followed by a 1-hour incubation with the secondary antibody. Cell nuclei were stained with DAPI for 10 minutes prior to imaging.

***S1.22 Tumor models and treatment experiments.***

To establish a unilateral tumor model, we subcutaneously injected 5×10^5^ CT26 cells or 1×10^6^ 4T1 cells into the right flank of female BALB/c mice (6-8 weeks). Treatment began when the tumor size reached 80-100 mm^3^. The mice were randomly divided into six groups: control, MM, IMM, RT, RT+MM, and RT+IMM. Four hours before RT, mice in each group received intratumoral injections of PBS/MM/IMM. For groups undergoing RT, mice were anesthetized and placed in individual lead boxes to expose the tumor area to irradiation while shielding the rest of the body. Radiotherapy was administered using an X-ray irradiator (Rad Source RS2000) with parameters set at 160 KV and 25mA. Following treatment, tumor size and body weight were measured every other day. Tumor volume (mm^3^) was calculated using the formula (length × width^2^) × 0.5. Euthanasia was performed if the tumor volume exceeded 2000 mm^3^ or if signs of distress were observed.

To establish a bilateral tumor model, we subcutaneously injected 5×10^5^ CT26 cells into the right flank of female BALB/c mice as the primary tumor and 5×10^4^ cells into the left flank as the distant tumor. Treatment began when the tumor size reached 100-150 mm^3^. The mice were randomly divided into five groups: control, RT, RT+IMM, RT+anti-PD-1, and RT+MM+anti-PD-1. Four hours before RT, mice in each group received intratumoral injections of PBS/ IMM in the primary tumor. For mice in groups receiving anti-PD-1 treatment, anti-PD-1 was administered via intraperitoneal injection (20 mg/kg on Day 0, 10 mg/kg on Day 1 and Day 2). The RT of the primary tumor, the subsequent monitoring of bilateral tumors, and the survival duration were the same as those of the aforementioned procedures.

To specifically evaluate long-term antitumor immune memory, an immune memory model was established using mice bearing unilateral CT26 tumors. Following treatment with RT+IMM in combination with anti-PD-1, animals that achieved sustained complete regression of the primary tumor, which was defined a priori as the absence of detectable tumor throughout the entire observation period, were designated as immune memory–competent and enrolled for subsequent tumor rechallenge experiments. Mice exhibiting residual or progressive tumors were not included in the rechallenge phase because they did not fulfill the predefined criteria for immune memory evaluation. Age- and sex-matched naive mice were set up as a control group. On day 90, a tumor rechallenge was conducted by inoculating 5×10^5^ CT26 tumor cells into the left flank of both the effective treatment model and naive mice. The subsequent monitoring of bilateral tumors and survival duration was the same as the aforementioned procedures.

To establish a humanized patient-derived xenograft (PDX) model, tumor tissue was obtained from a 32-year-old Han Chinese male with recurrent laryngeal squamous cell carcinoma following 33 sessions of RT at West China Hospital, Sichuan University. Demographic and clinical data were retrieved from the electronic medical records. Immediately after surgical resection, the fresh tumor specimen was placed on ice, immersed in MACS® Tissue Storage Solution (Miltenyi Biotec, Cat. No. 130-100-008), and delivered to the laboratory within two hours. Under sterile conditions, necrotic areas were removed, and viable tissue was cut into 3×3×3 mm^3^ fragments. For short-term use, samples were stored at 4°C; for long-term preservation, they were cryopreserved in liquid nitrogen using a solution of 10% DMSO and fetal bovine serum. The tumor fragments were then subcutaneously implanted into the right flank of NOD-SCID mice to establish F1-generation PDX models. After the tumors grew to a volume of 1000 mm^3^, they were propagated in a second generation of BALB/c nude mice (F2) and subsequently transferred to a third generation (F3) of mice for experimental purposes. Treatment began when the tumor size reached 80-110 mm^3^. The mice were randomly divided into three groups: control, IMM, RT, and RT+IMM. Four hours before RT, mice in each group received intratumoral injections of PBS/ IMM. For groups undergoing RT, mice were anesthetized with 1% pentobarbital sodium (100μL per 18g body weight) and placed in individual lead boxes to expose the tumor area to irradiation while shielding the rest of the body. RT was administered using an X-ray irradiator (Rad Source RS2000) with parameters set at 160 KV and 25mA. Following treatment, tumor size and body weight were measured every three days. All procedures were conducted in accordance with institutional guidelines and approved by the Ethics Committee on Biomedical Research, West China Hospital of Sichuan University (Ethics Approval No. 20211673), and comply with the WMA Declaration of Helsinki. Written informed consent was obtained from all patients involved.

***S1.23 Flow cytometry analysis.***

The isolated tumor tissues or spleens were digested into single-cell suspension and pre-incubated (30 min) with FVS440UV (BD, Catalog No. 566332, 1:1000). Subsequently, the cell suspension was incubated with purified Rat Anti-Mouse CD16/CD32 (BD, Catalog No. 553141, 0.5 μg per test) to block nonspecific binding, and then stained with anti-CD45-APC-CY7 (Catalog No. 557659), anti-CD3-BUV395 (Catalog No. 563565), anti-CD4-BUV661 (Catalog No. 741461), anti-CD8-BV771 (Catalog No. 563046), anti-CD69-PE-CY7 (Catalog No. 552879), anti-CD279-BV650 (Catalog No. 744546), anti-CD44-PerCP-Cy5.5 (Catalog No. 560570), anti-CD62L-FITC (Catalog No. 561917), anti-CD11c-BUV563 (Catalog No. 749091), anti-MHC II-R718 (Catalog No. 752163). All antibodies were diluted to the working concentration of 0.2 μg per test. Finally, the stained cells were filtered and detected by FACS FCM (BD, FACSymphony A5) and analyzed by Flowjo software (10.8.1).

***S1.24 In vivo histopathological analysis.***

Tumor tissues and organs **(**heart, liver, spleen, lung, and kidney) collected from mice were fixed in 4% formalin for 48 hours, then embedded in paraffin, and sectioned into 4 μm slices. The slides were subjected to hematoxylin and eosin (H&E) staining and the TUNEL assay following established protocols from previous studies. For immunofluorescence, tissue sections were first treated with γ-H2AX (GB111841, Servicebio, 1/100), followed by labeling with Cy3-conjugated goat anti-rabbit IgG (GB21303, Servicebio,1/300). Another set of tissue sections was treated with Ki67 (GB111141, Servicebio, 1/500) and labeled with Cy3-conjugated goat anti-rabbit IgG (GB21303, Servicebio, 1/300). For multiplex immunofluorescence (mIF), tissue sections were treated with mIF antibodies against CD8 (GB15068, Servicebio, 1/1000), CD11c (GB11059, Servicebio, 1/1000), α-SMA (19245, CST, 1/200), CD31 (ab182981, abcam, 1/500), and HIF-1α (NB100-105, Novus, 1/100). After incubation with peroxidase (HRP)-conjugated secondary antibodies, signals were developed using tyramide signal amplification (TSA) with appropriate fluorophores, followed by nuclear counterstaining with DAPI.

***S1.25 In vivo biocompatibility study.***

Blood samples were obtained from mice on the 12th day after treatment and analyzed for biochemical parameters by Lilai Biotechnology Co., Ltd. (Chengdu, China). Simultaneously, key organs—including the heart, liver, spleen, lungs, and kidneys—were harvested, fixed, embedded in paraffin, sectioned, and subjected to H&E staining following standard procedures. Histological images were captured using the Olympus VS200 imaging system (Olympus Life Science, Japan).

***S1.26 In vivo biodistribution study.***

Computed Tomography (CT) imaging (NEMO micro-CT, Pingseng Scientific, China) was performed on BALB/c mice with CT26 tumors that had received intratumoral injections of IMM. The scans were conducted at five time points: before injection and at 4 hours, 6 days, 12 days, and 18 days post-injection. Additionally, DICOM images from the CT scans were processed and reconstructed into three-dimensional models using Imaris software (version 10.2). Furthermore, organ samples (heart, liver, spleen, lungs, and kidneys) and metabolic excreta (urine and feces) were collected 6, 12, and 18 days post-injection. Each sample was weighed, treated with aqua regia, and incubated at 80 °C until completely dissolved. The supernatant was then extracted and analyzed using inductively coupled plasma mass spectrometry (ICP-MS, Agilent 7850) to determine iridium levels.

***S1.27 RNA-seq and bioinformatics analysis.***

Total RNA was extracted from tumor tissues using TROzol Reagent (Thermo Fisher, 15596018) according to the manufacturer's protocols. RNA sequencing analysis was conducted by LC-Bio Technology Co., Ltd., Hangzhou, China. Statistical analyses, including principal component analysis (PCA), differential gene expression, KEGG pathway analysis, and GO enrichment analysis, were performed using the DESeq2 and clusterProfiler packages.

***S1.28 Statistical analysis.***

The quantitative data were expressed as mean ± S.D. Statistical differences were calculated by GraphPad Prism software (version 10) using unpaired student’s t-test or one-way analysis of variance (ANOVA) when comparing two or multiple groups, respectively.

**S2 Supplementary Figures**


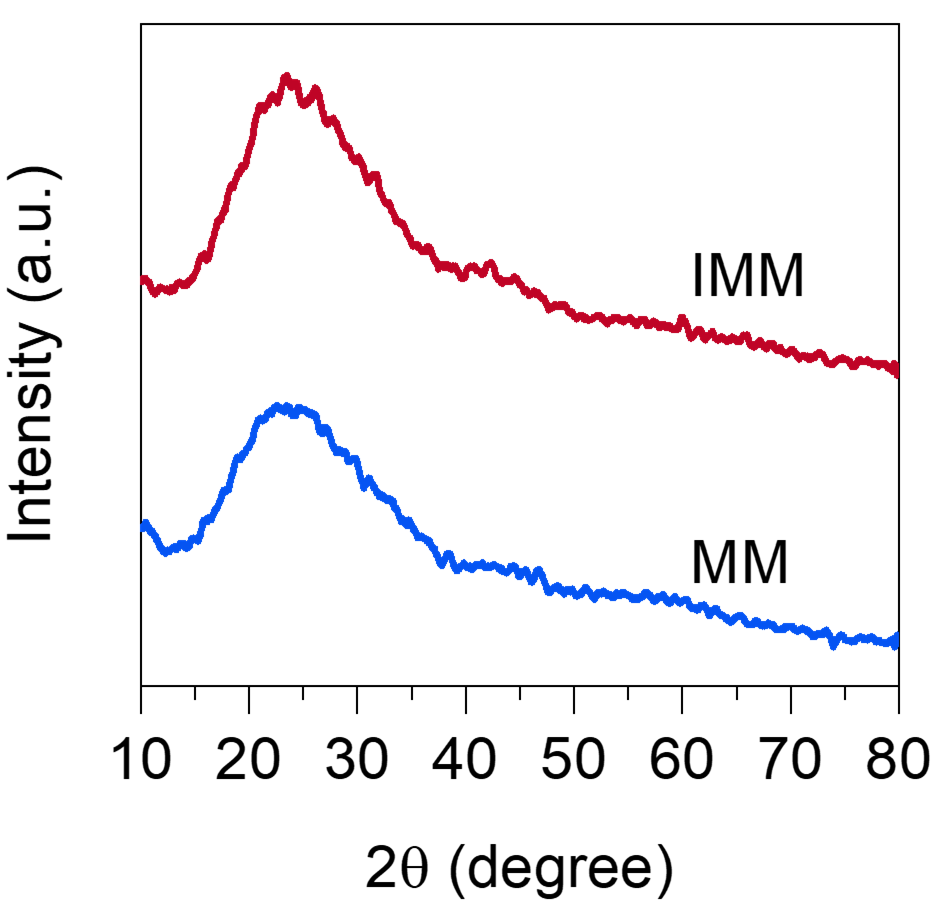


**Fig. S1** XRD spectra of IMM and MM.


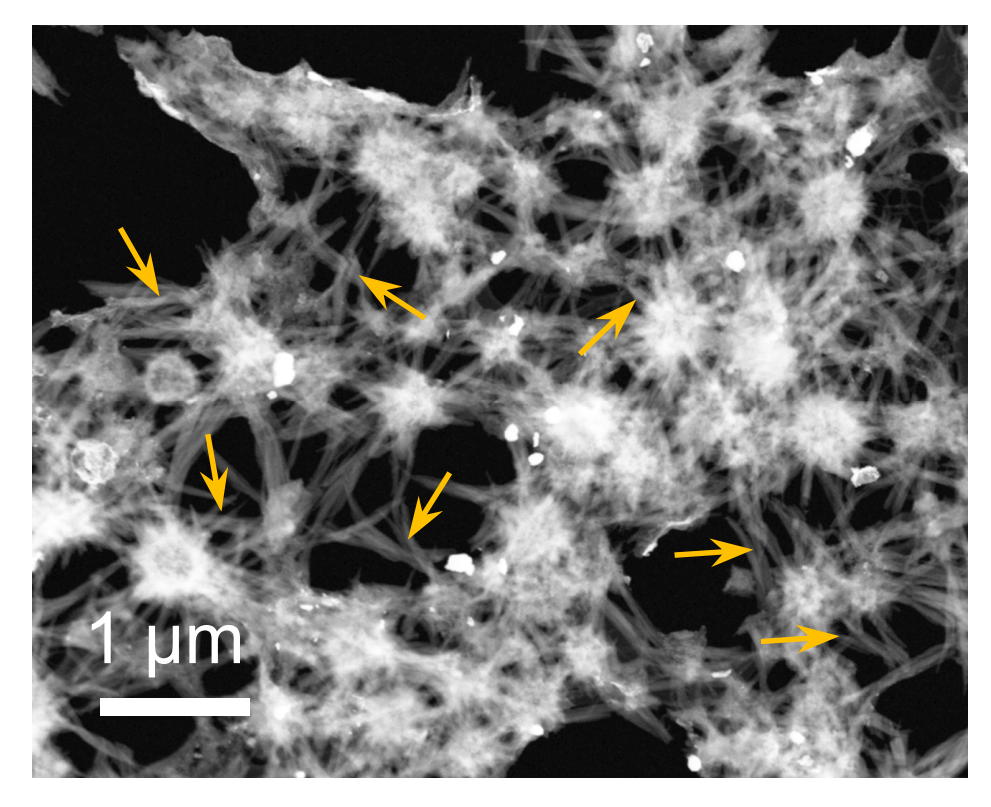


**Fig. S2** HAADF-STEM image of IMM, showing the spiky morphology indicated by yellow arrows.


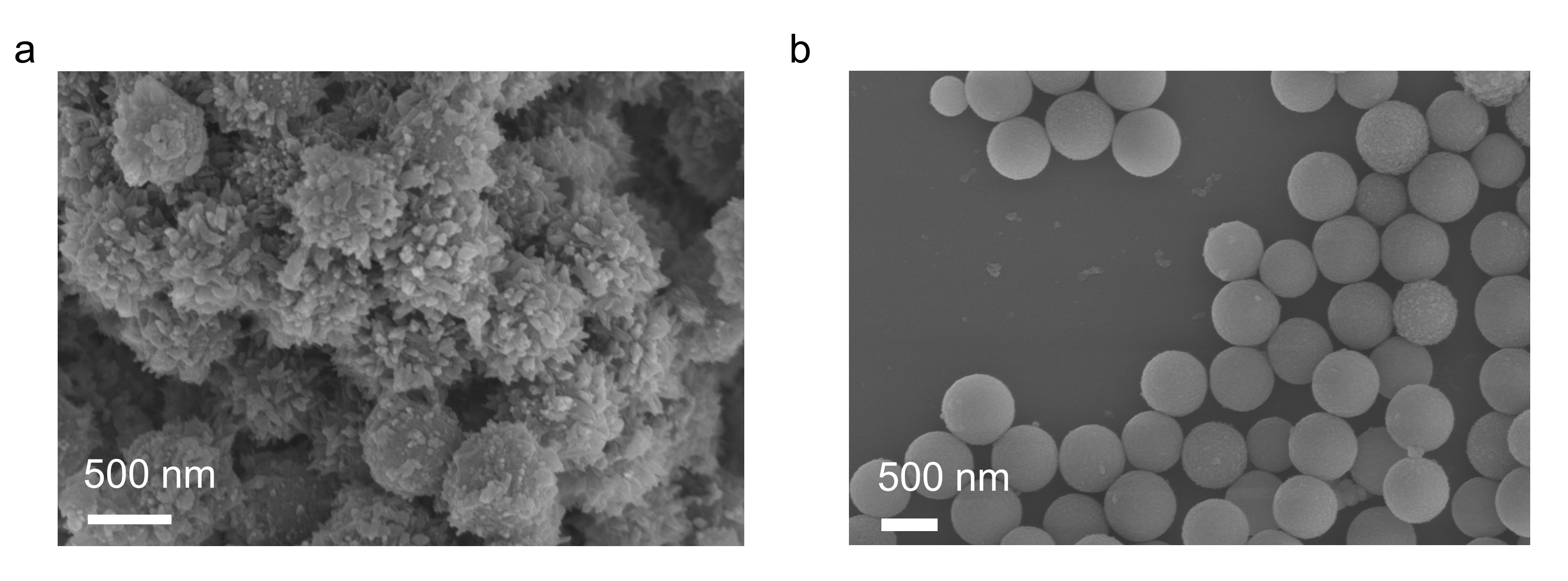


**Fig. S3** SEM images of a) IMM, b) MM.


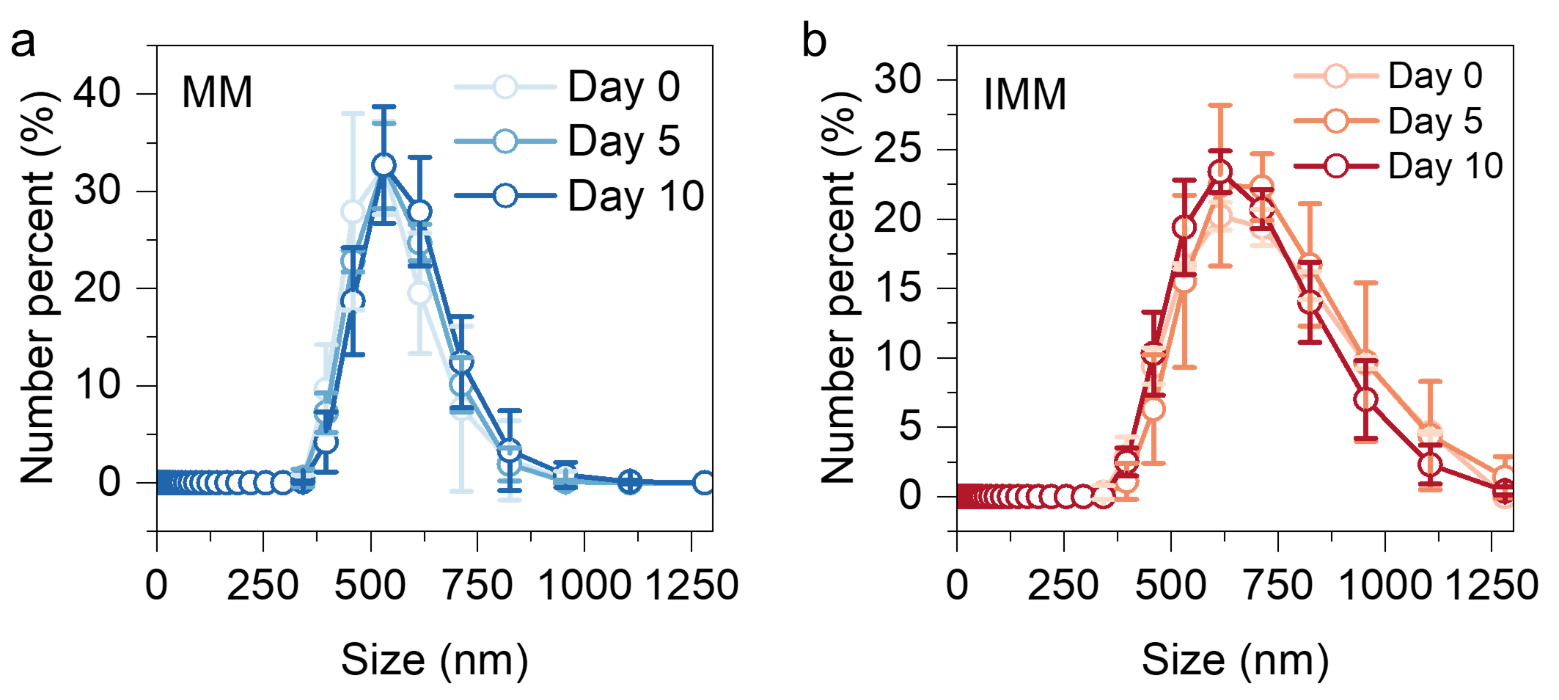


**Fig. S4** Dynamic light scattering analysis of a) MM and b) IMM was conducted in deionized water (n = 3 independent experiments, data are presented as mean ± SD).


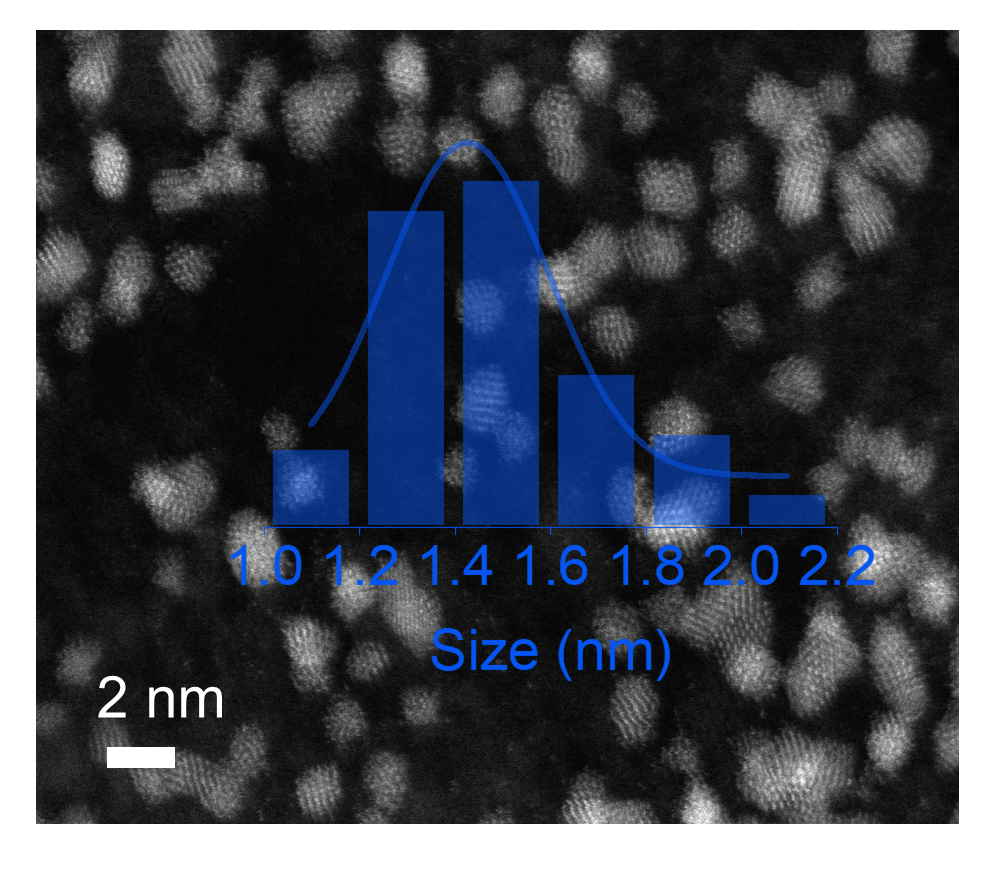


**Fig. S5** HAADF-STEM images and Ir nanoclusters’ size distribution in IMM.


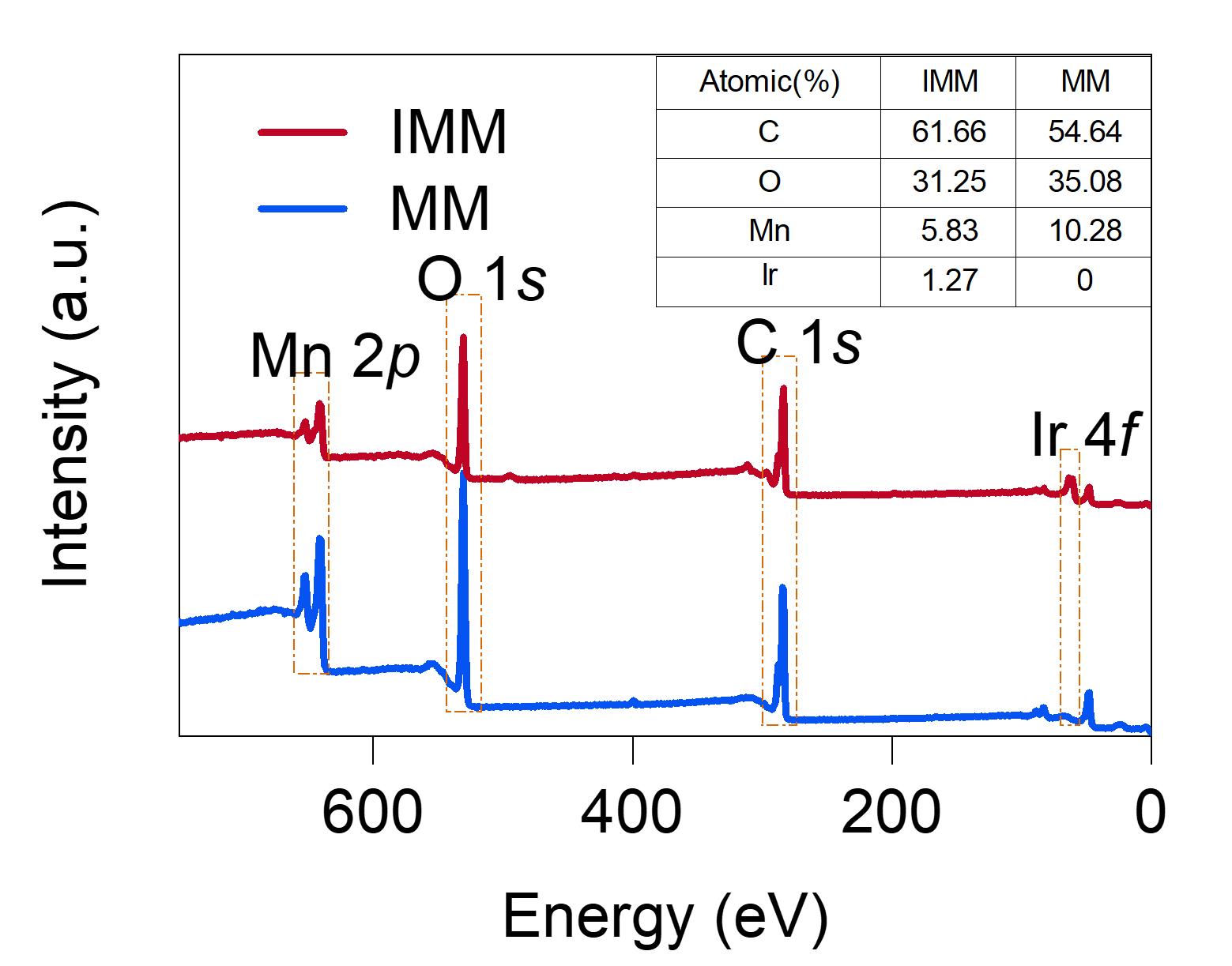


**Fig. S6** XPS survey scan spectra of IMM and MM.


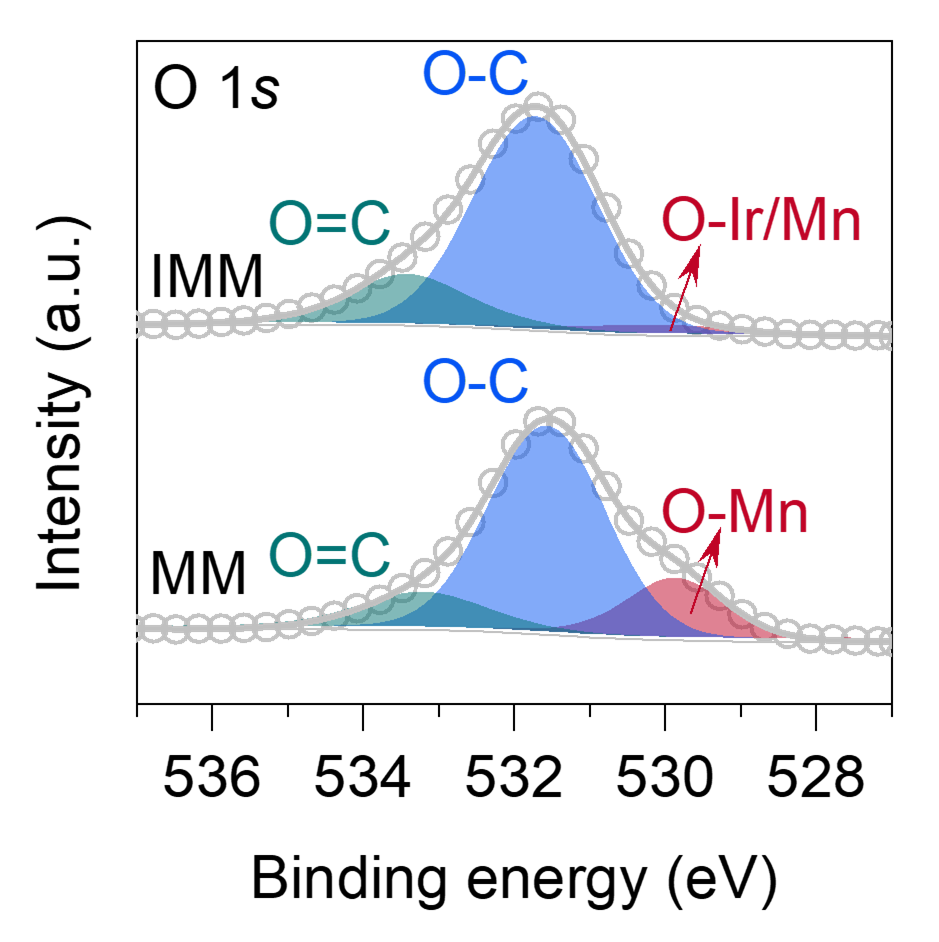


**Fig. S7** High-resolution XPS spectra of O 1*s* of IMM and MM.


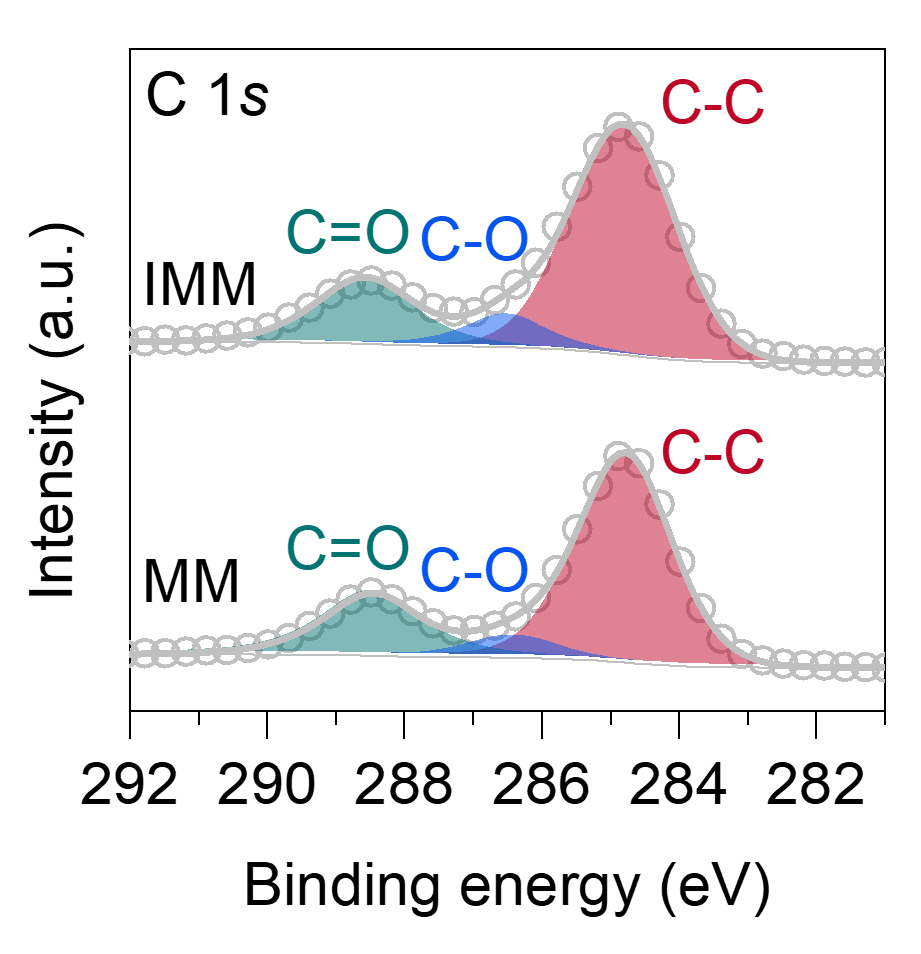


**Fig. S8** High-resolution XPS spectra of C 1*s* of IMM and MM.


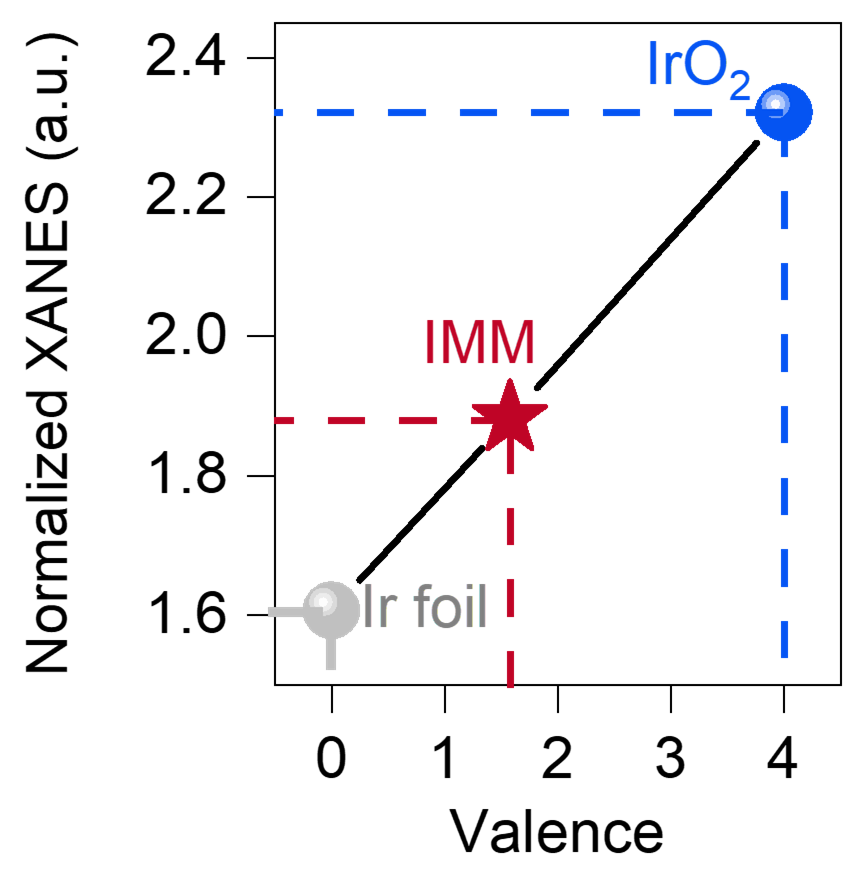


**Fig. S9** Valence analysis of Ir species in IMM.


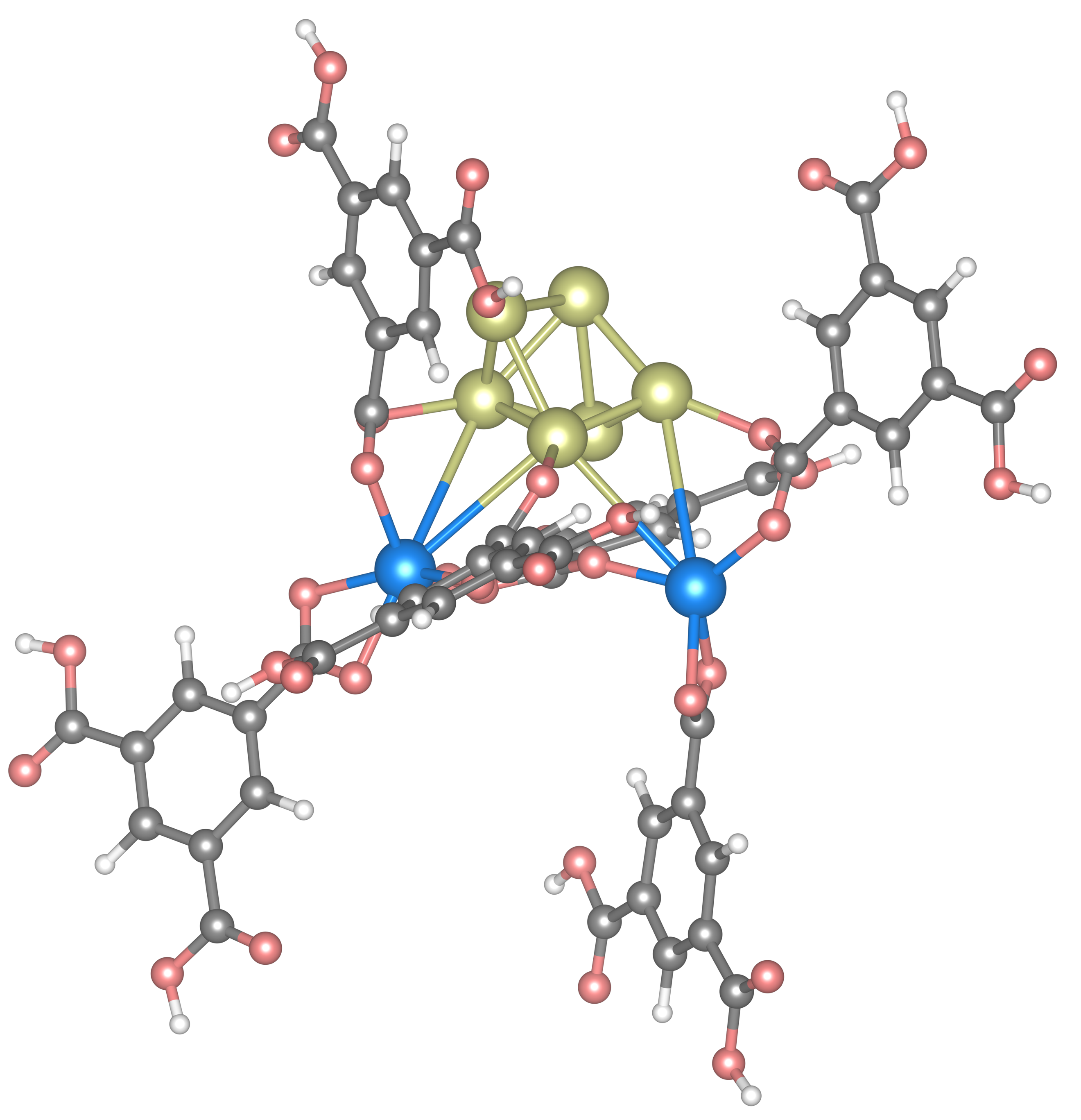


**Fig. S10** Theoretical structure model of IMM. Atomic color coding in the structure: Ir, yellow; Mn, blue; C, gray; O, red; and H, white.


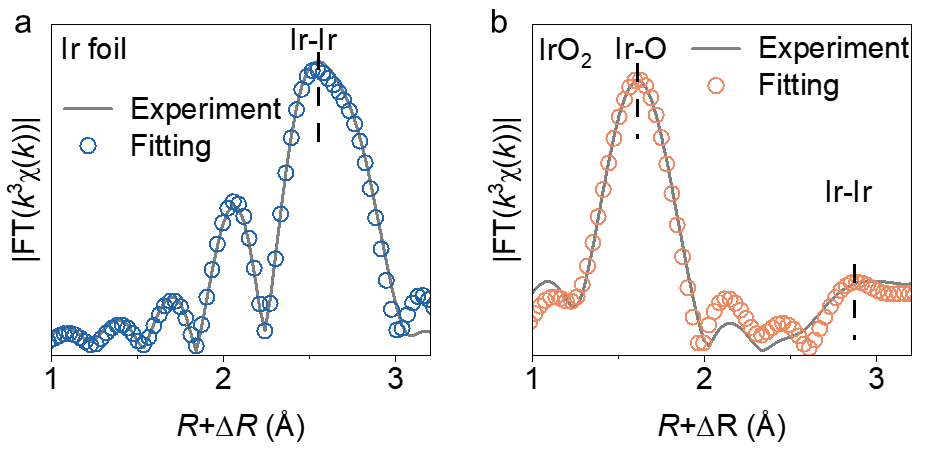


**Fig. S11** Experimental and fitted EXAFS spectra of a) Ir foil and b) IrO_2_.


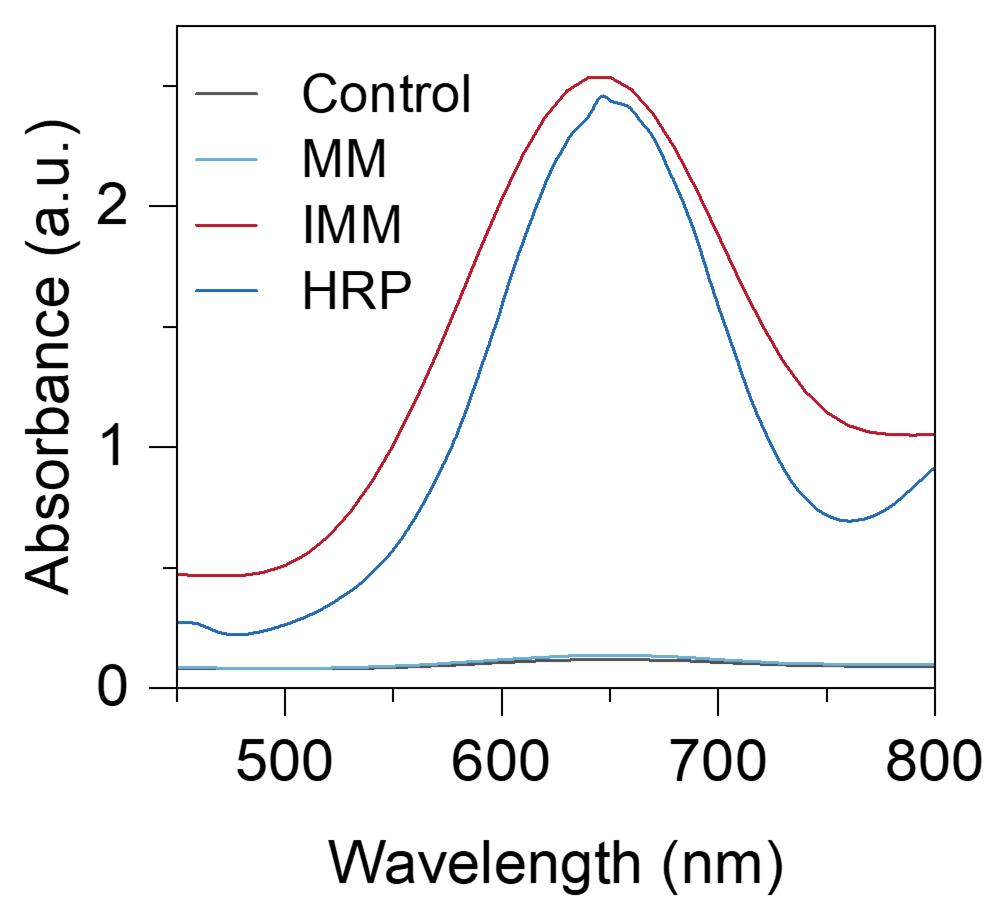


**Fig. S12** POD-like activities employing TMB-based UV–vis spectra in the presence of horseradish HRP and biocatalytic materials.


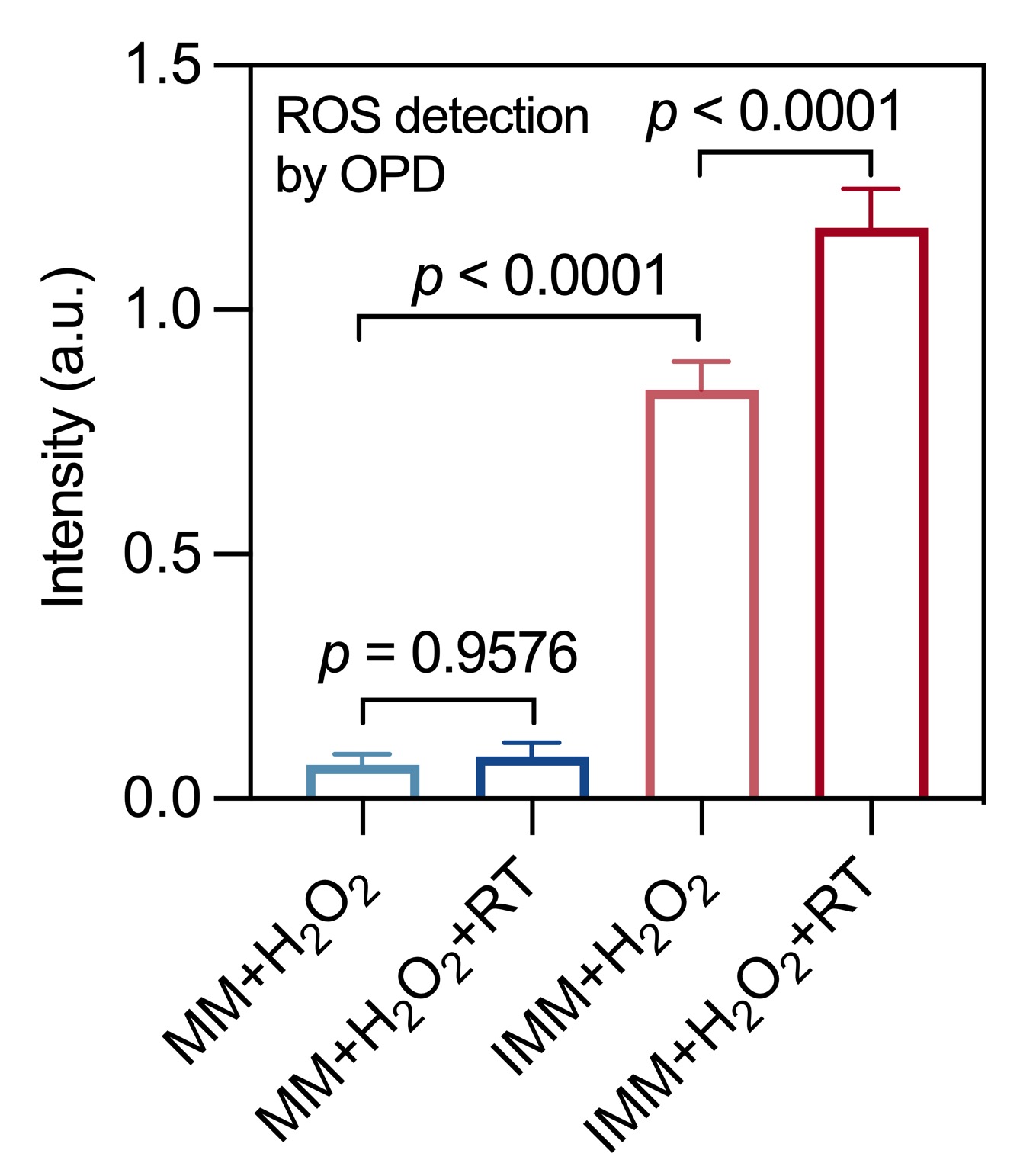


**Fig. S13** Detection of ROS generation using OPD (n = 5 independent replicates).


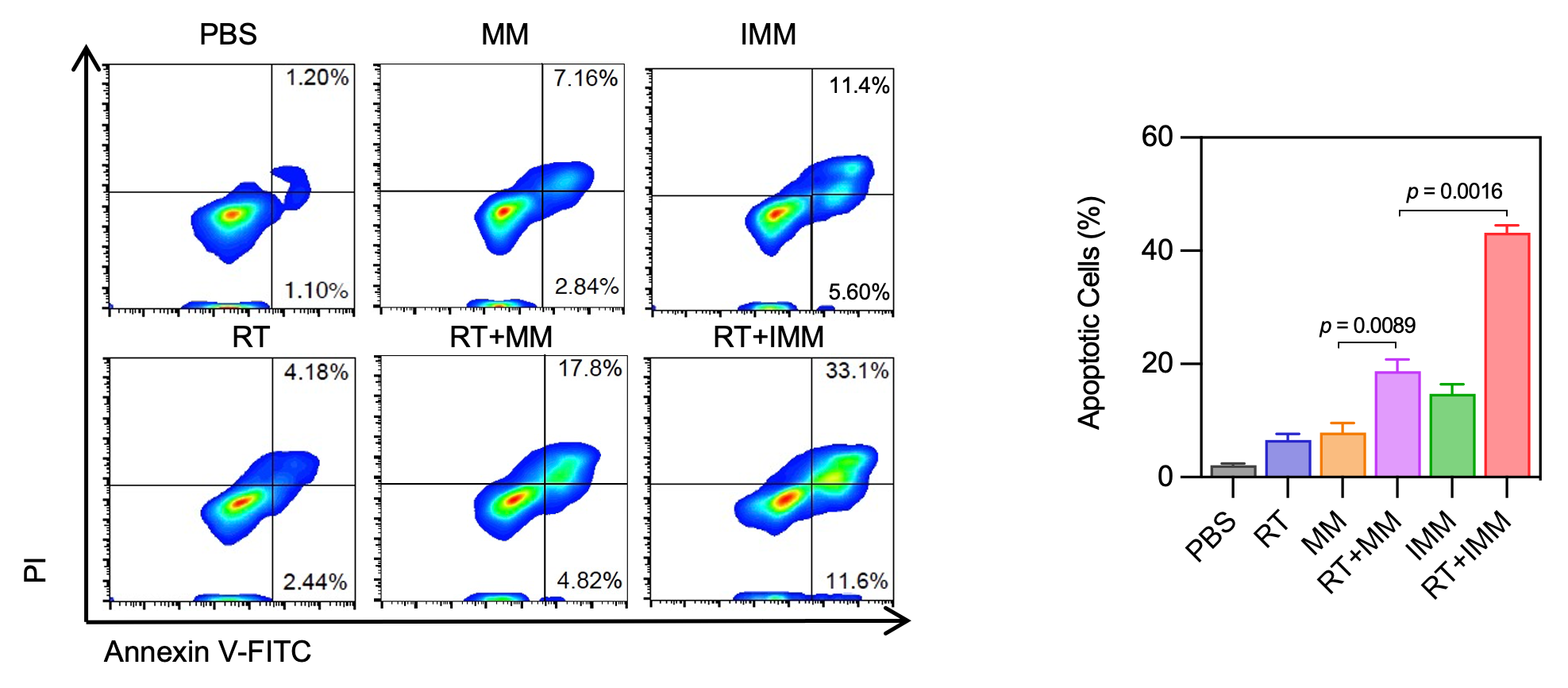


**Fig. S14** Annexin V/PI analysis of CT26 cells. Cells were incubated with PBS, MM, and IMM, with and without X-ray irradiation at a dose of 6 Gy. The graphs show the percentage of cells in each quadrant. Error bars represent standard deviation values. Experiments were repeated independently three times with similar results (n = 3 independent replicates).


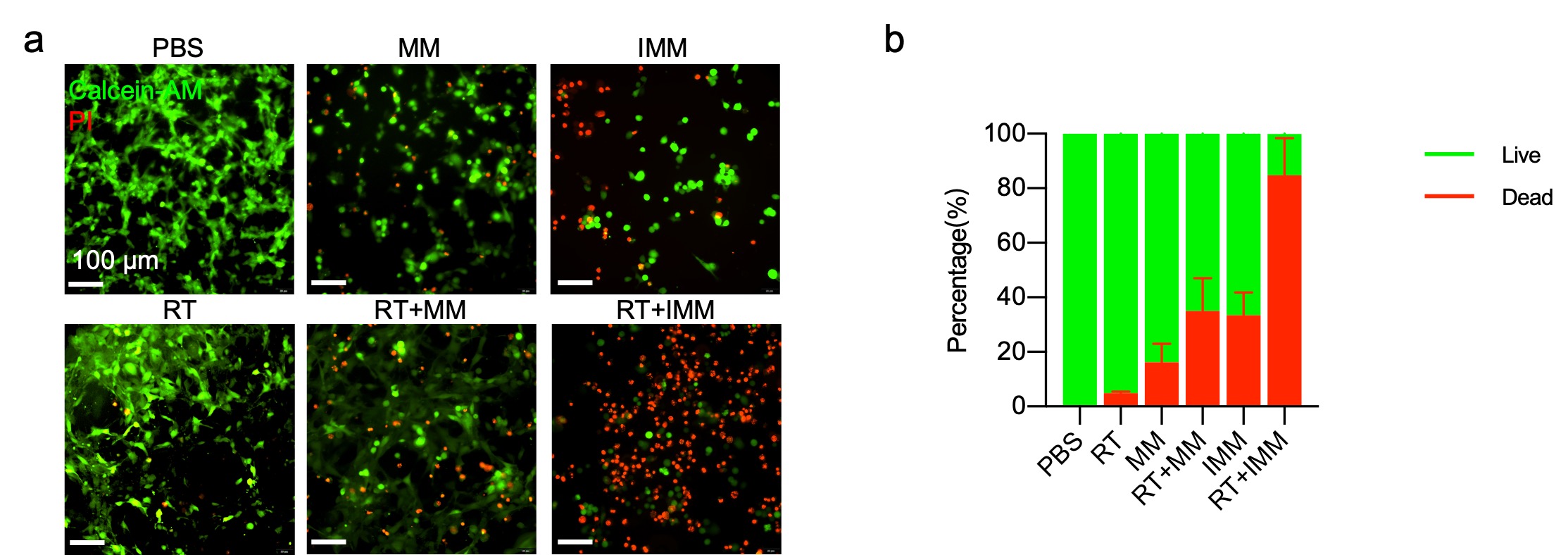


**Fig. S15** Live/dead analysis of CT26 cells after different treatments. a) Representative fluorescence images from Live/dead analysis of CT26 cells after different treatments (Green: live cells; Red: dead cells; scale bar = 100 μm). Experiments were repeated independently three times with similar results. b) The graph shows the percentage of dead cells in different groups (n = 3 independent replicates).


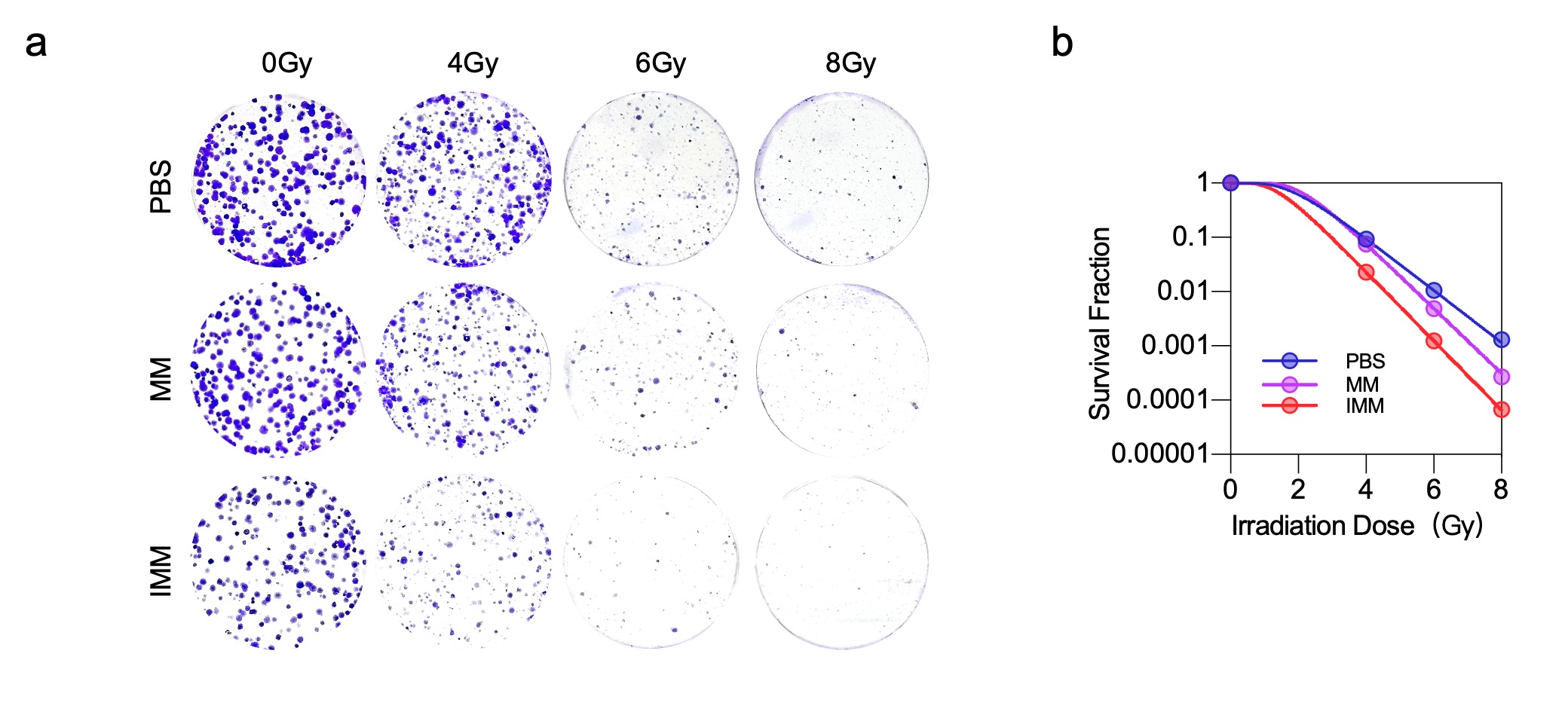


**Fig. S16** Clonogenic assay. a) Representative images from the Clonogenic assay of SCC7 cells after different treatments. Experiments were repeated independently three times with similar results. b) The graph shows the radio-enhancement effect of MM and IMM on SCC7 cells (n = 3 independent replicates).


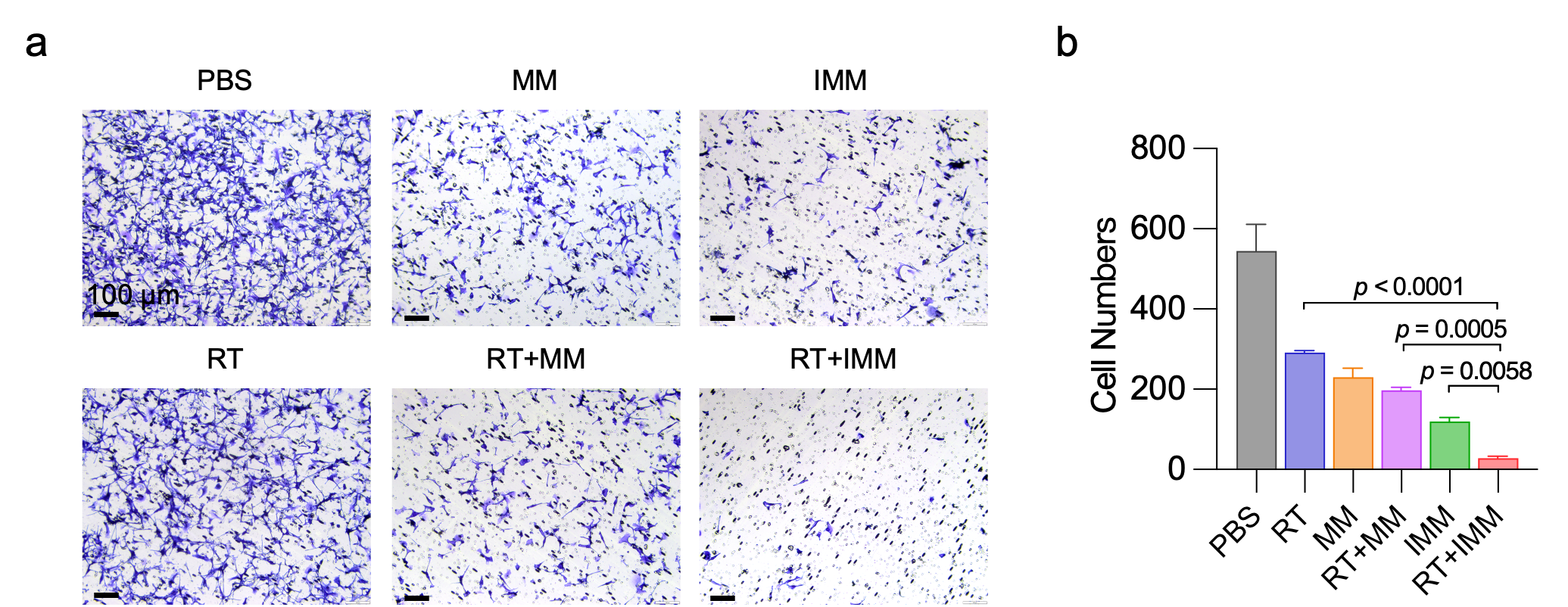


**Fig. S17** Transwell migration assay. a) Representative images display the migration of CT26 cells (scale bar = 100 μm). Experiments were repeated independently three times with similar results. b) The graph shows the number of migrated cells in different groups (n = 3 independent replicates).


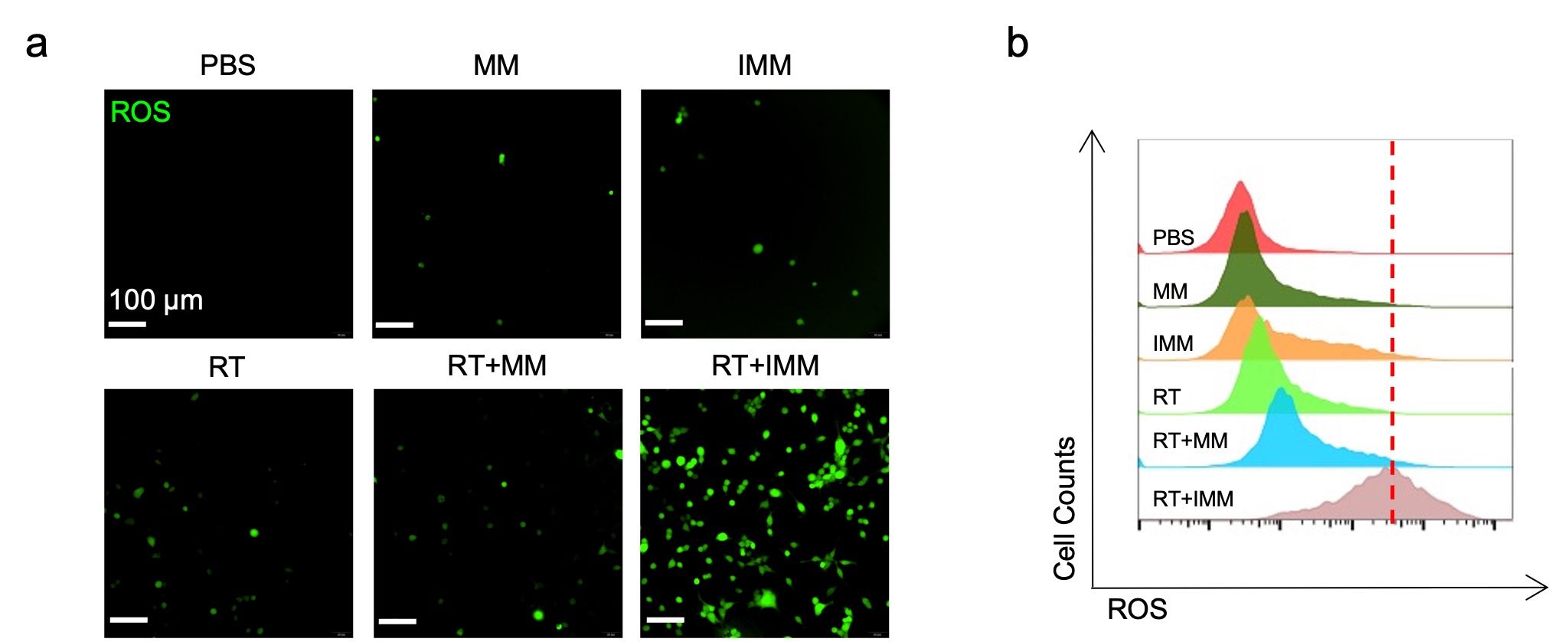


**Fig. S18** ROS production in CT26 cells after different treatments. a) Representative images show ROS generation across different groups (scale bar = 100 μm). Experiments were repeated independently three times with similar results. b) The cells were collected to evaluate ROS generation in CT26 using quantitative flow cytometric analysis.


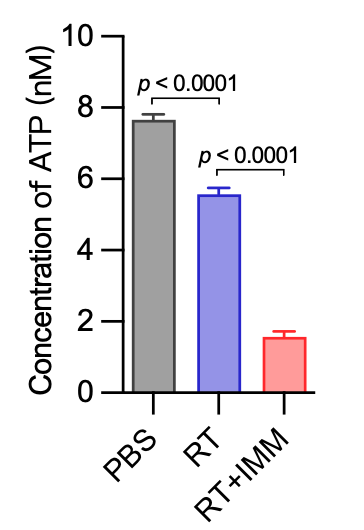


**Fig. S19** Intracellular ATP concentration (n = 3 independent replicates).


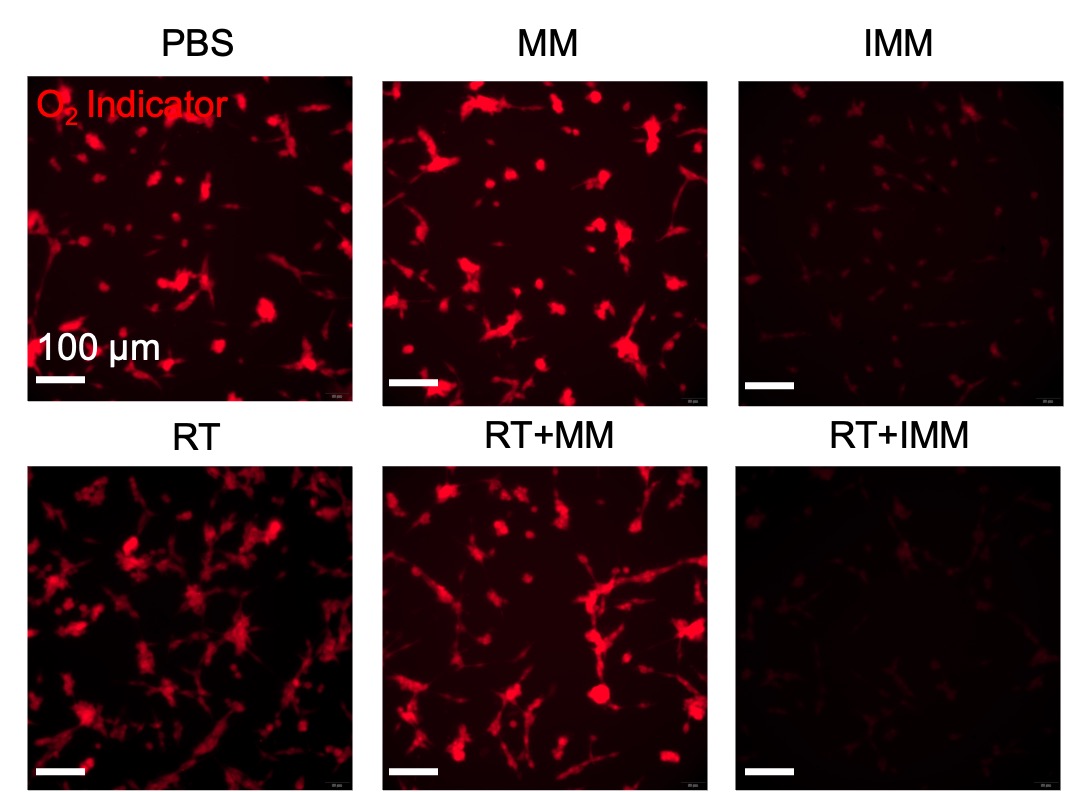


**Fig. S20** The oxygen generation of MM/IMM. The O_2_ generated by different treatments was probed with [Ru(bpy)_3_]Cl_2_. The red fluorescence is inversely dependent on the concentration of oxygen (scale bar = 100 μm). Experiments were repeated independently three times with similar results.


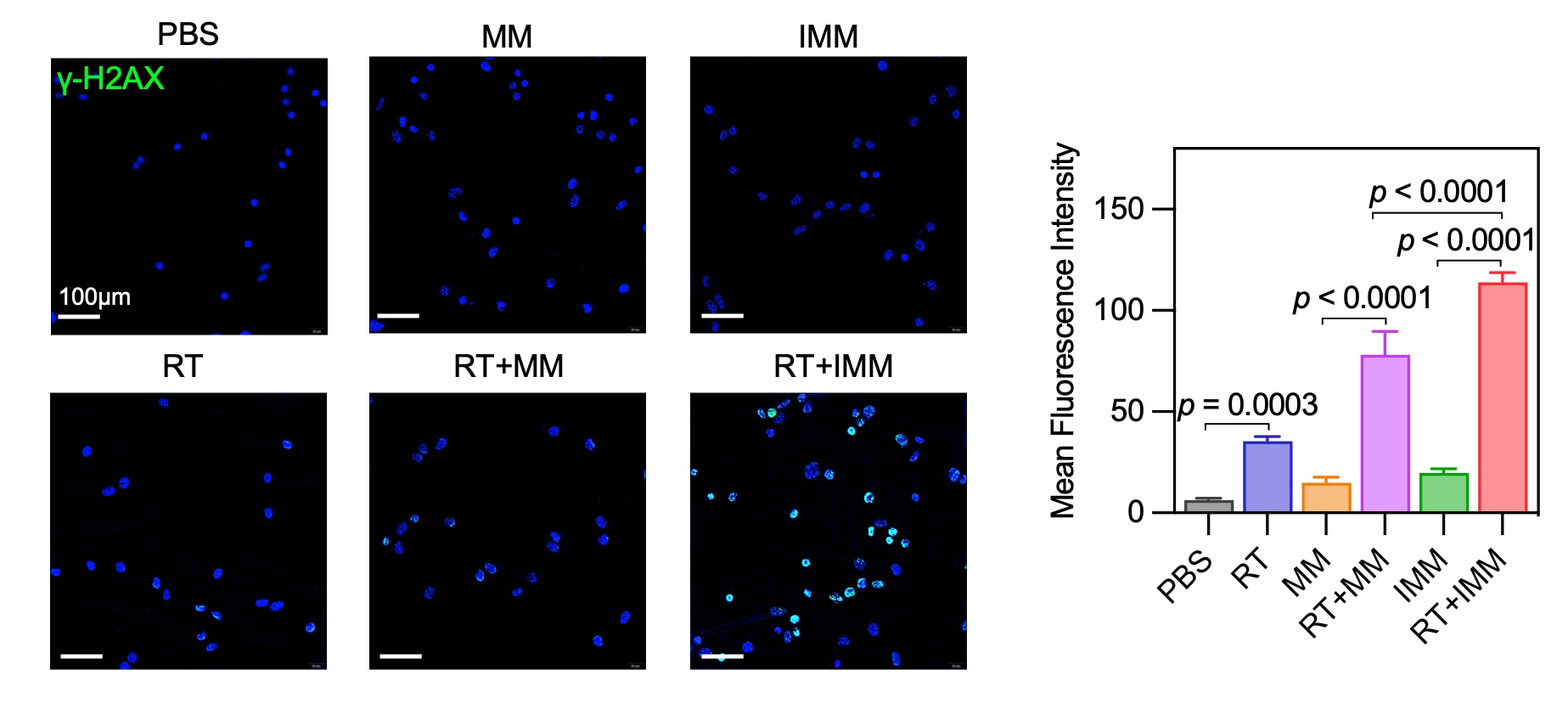


**Fig. S21** γ-H2AX immunofluorescence assay. Representative γ-H2AX immunofluorescence images of each treatment group (scale bar = 100 μm) and quantification of mean γ-H2AX fluorescence intensity across groups. Experiments were repeated independently three times with similar results.


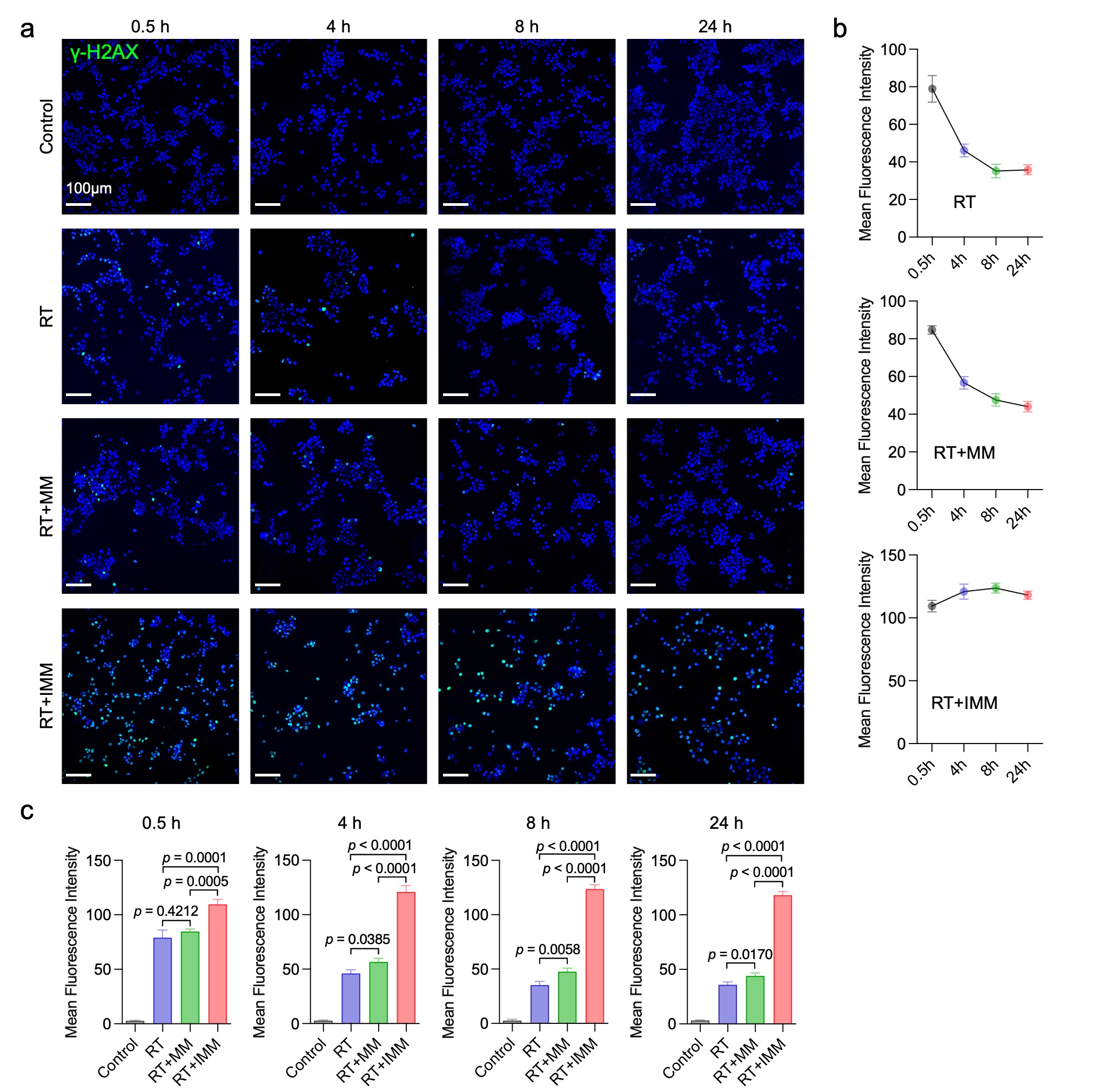


**Fig. S2****2 γ-H2AX immunofluorescence analysis at multiple post-treatment time points.** a) Representative γ-H2AX immunofluorescence images of each treatment group at the indicated time points (scale bar = 100 μm). Experiments were repeated independently three times with similar results. b) Time-course curves of mean γ-H2AX fluorescence intensity in each group (n = 3 independent replicates). c) Quantification of mean γ-H2AX fluorescence intensity across groups at the corresponding time points (n = 3 independent replicates).


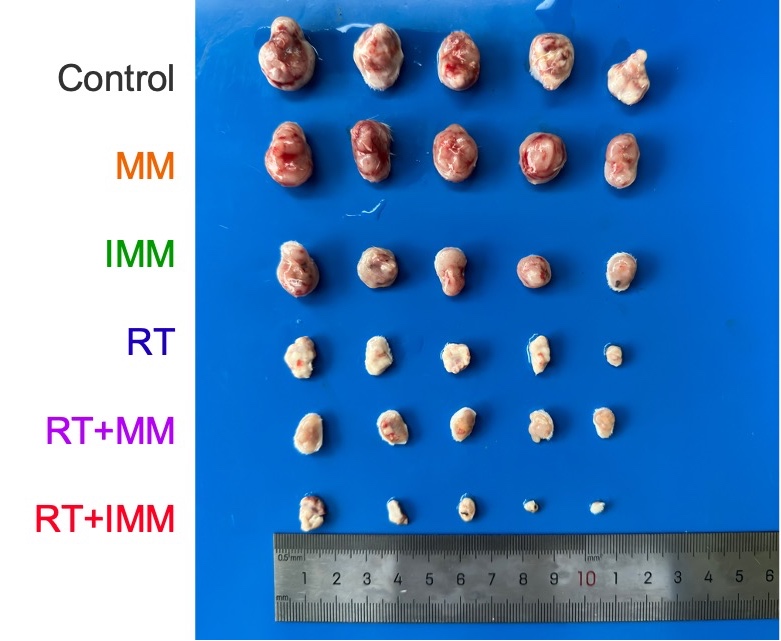


**Fig. S23** Representative images of CT26 subcutaneous tumors harvested on day 12 after treatment with saline, MM, IMM, or combined with radiotherapy (n = 5 biologically independent mice per group). Experiments were repeated independently three times with similar results.

**
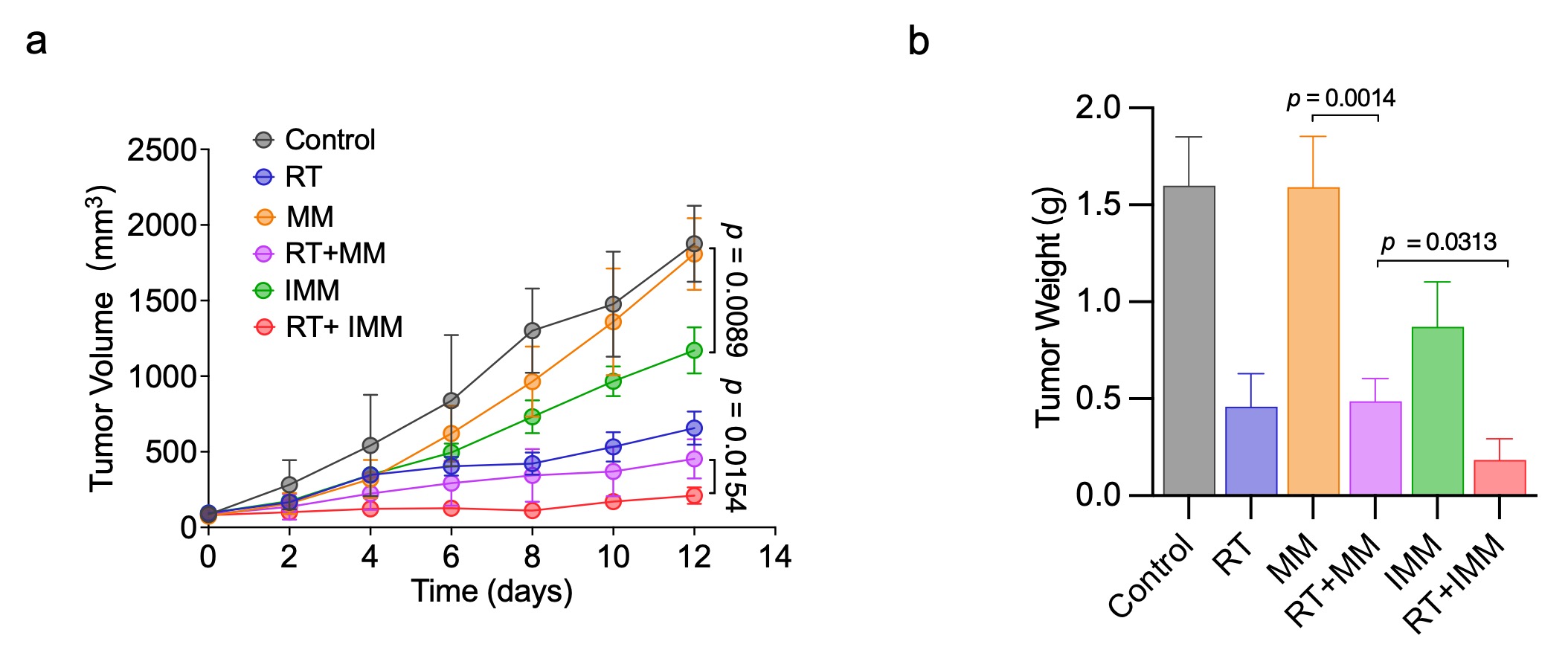
**

**Fig. S24** a) Average tumor growth curves and b) tumor weight of CT26 tumor-bearing mice after different treatments (n = 5 biologically independent mice per group).


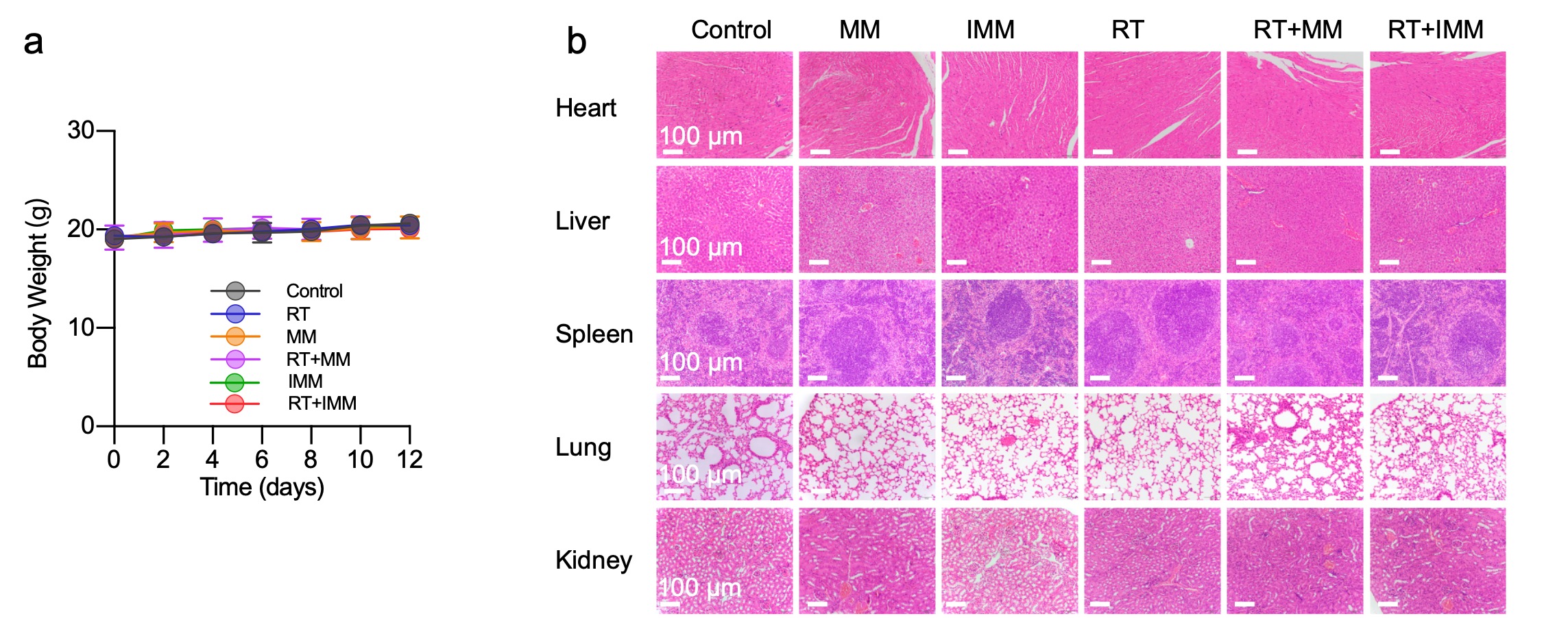


**Fig. S25** *In vivo* safety evaluation of different treatments. a) Body weight changes of BALB/c mice after treatments (n = 5 biologically independent mice per group). b) Representative H&E-stained tissue sections from each group (scale bar = 100 μm). Experiments were repeated independently three times with similar results.


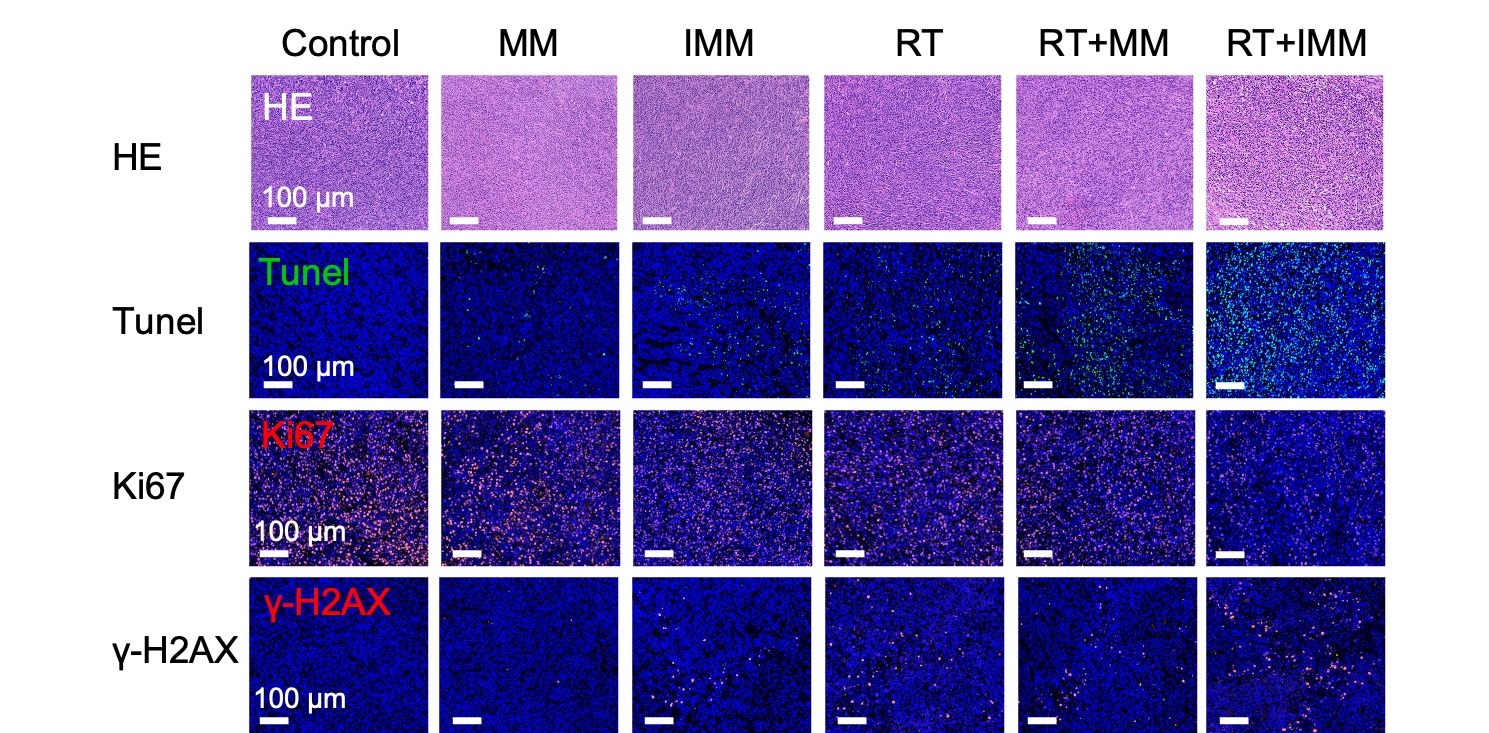


**Fig. S26** *In vivo* antitumor efficacy evaluation of different treatments. Representative H&E-stained, TUNEL assay, γ-H2AX detection, and Ki67 detection tissue sections from each group (scale bar = 100 μm). Experiments were repeated independently three times with similar results.


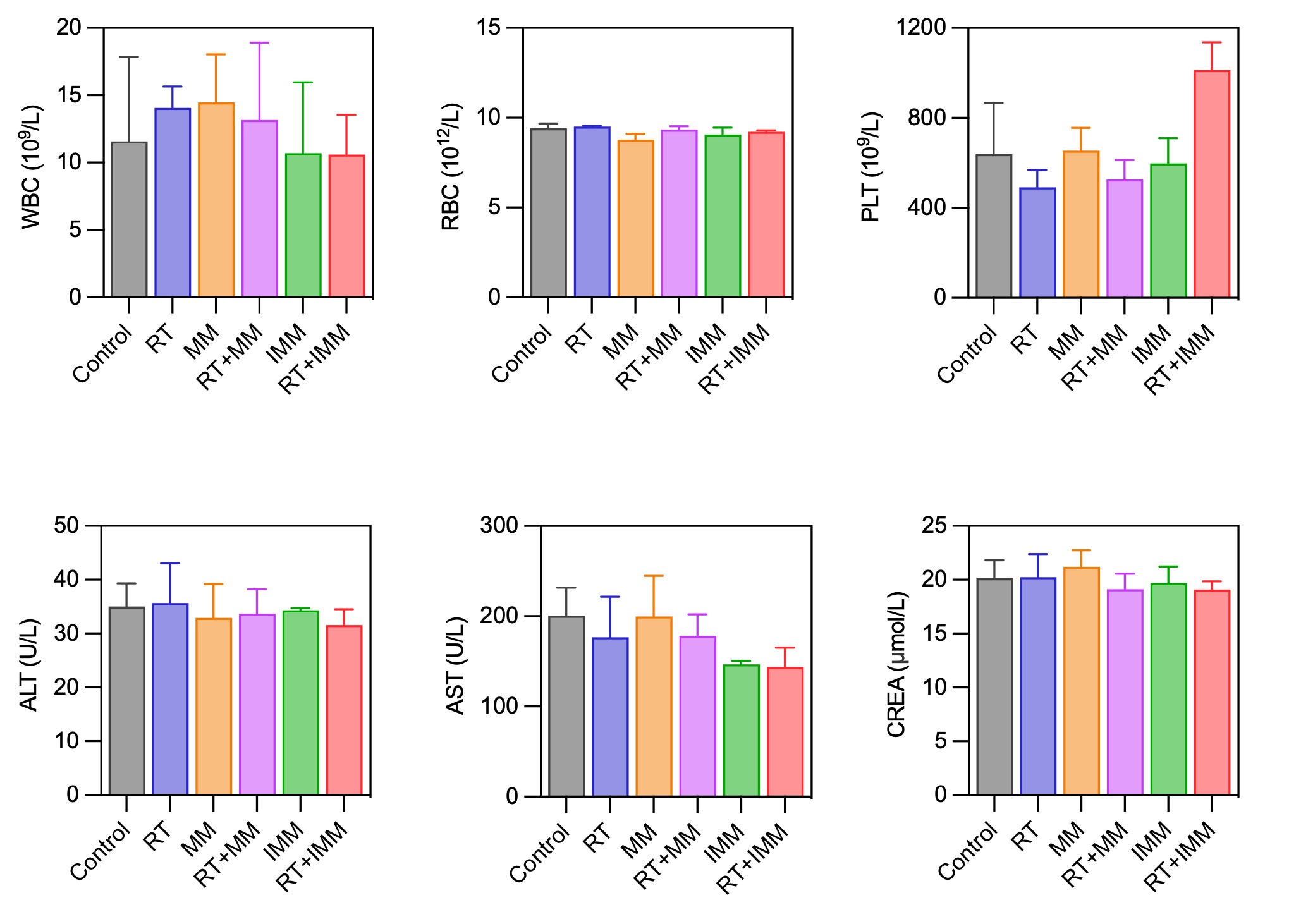


**Fig. S27** Results of blood chemistry parameters from mice after different treatments (n = 3 biologically independent mice per group). Blood chemistry parameters include white blood cell (WBC), red blood cell (RBC), platelet count (PLT), alanine aminotransferase (ALT), aspartate aminotransferase (AST), and creatinine (CREA).

**
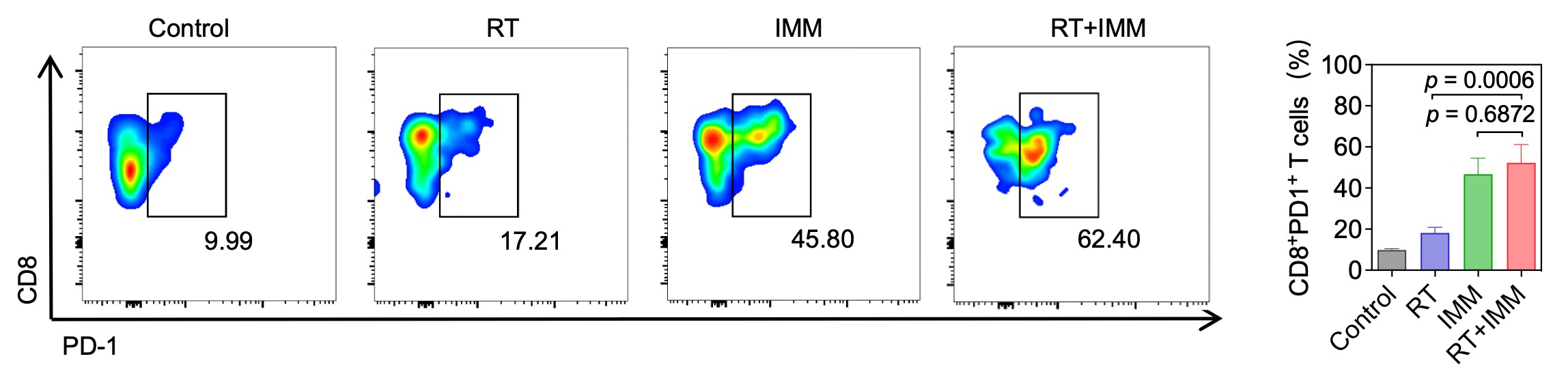
**

**Fig. S28** Representative flow cytometric analysis and relative quantification of CD8^+^PD-1^+^T cells (n = 5 biologically independent mice per group).


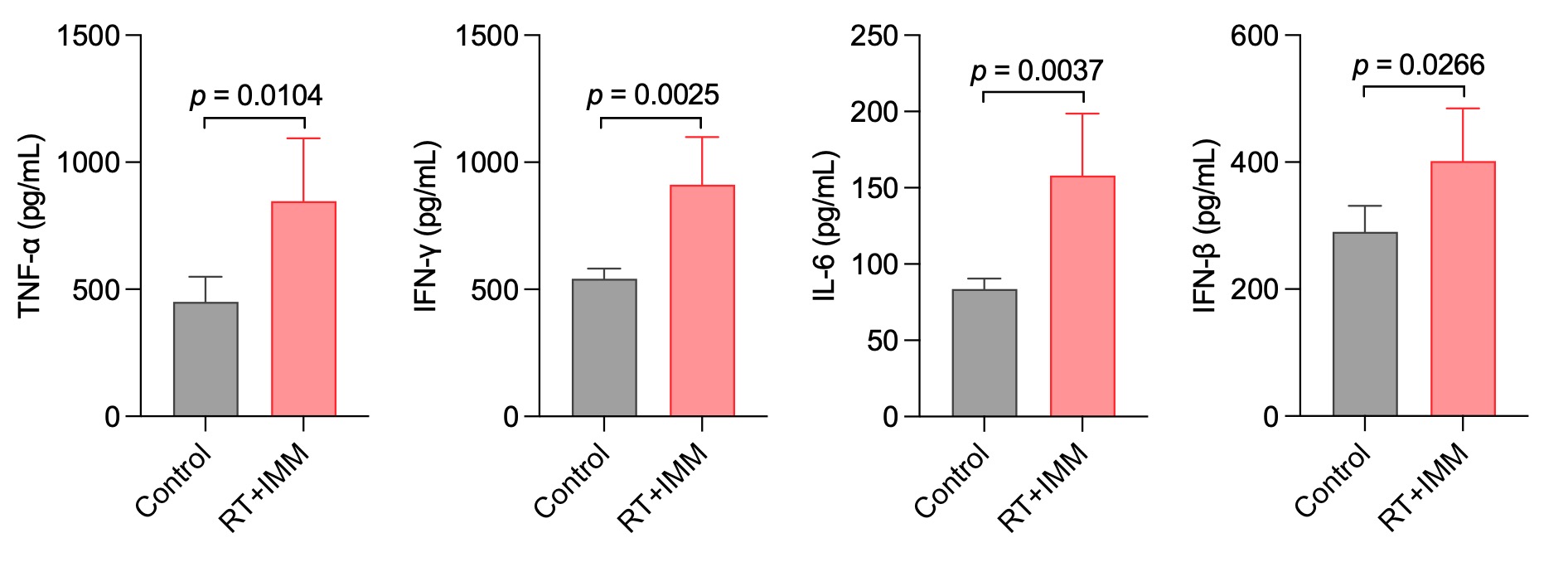


**Fig. S29** Quantification of serum cytokines (TNF-α, IL-6, IFN-β, and IFN-γ) in mice following different treatment regimens (n = 5 biologically independent mice per group).

**
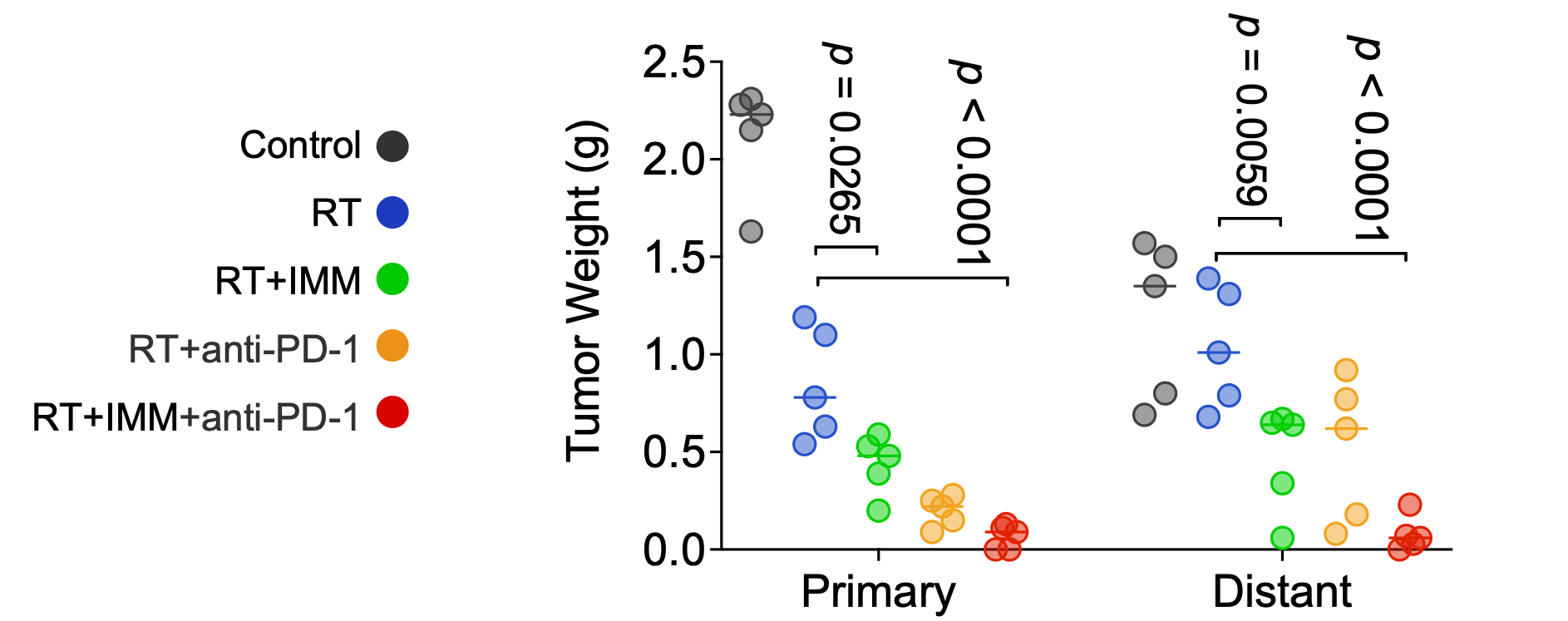
**

**Fig. S30** Tumor weight of CT26 tumor-bearing mice after different treatments.

**
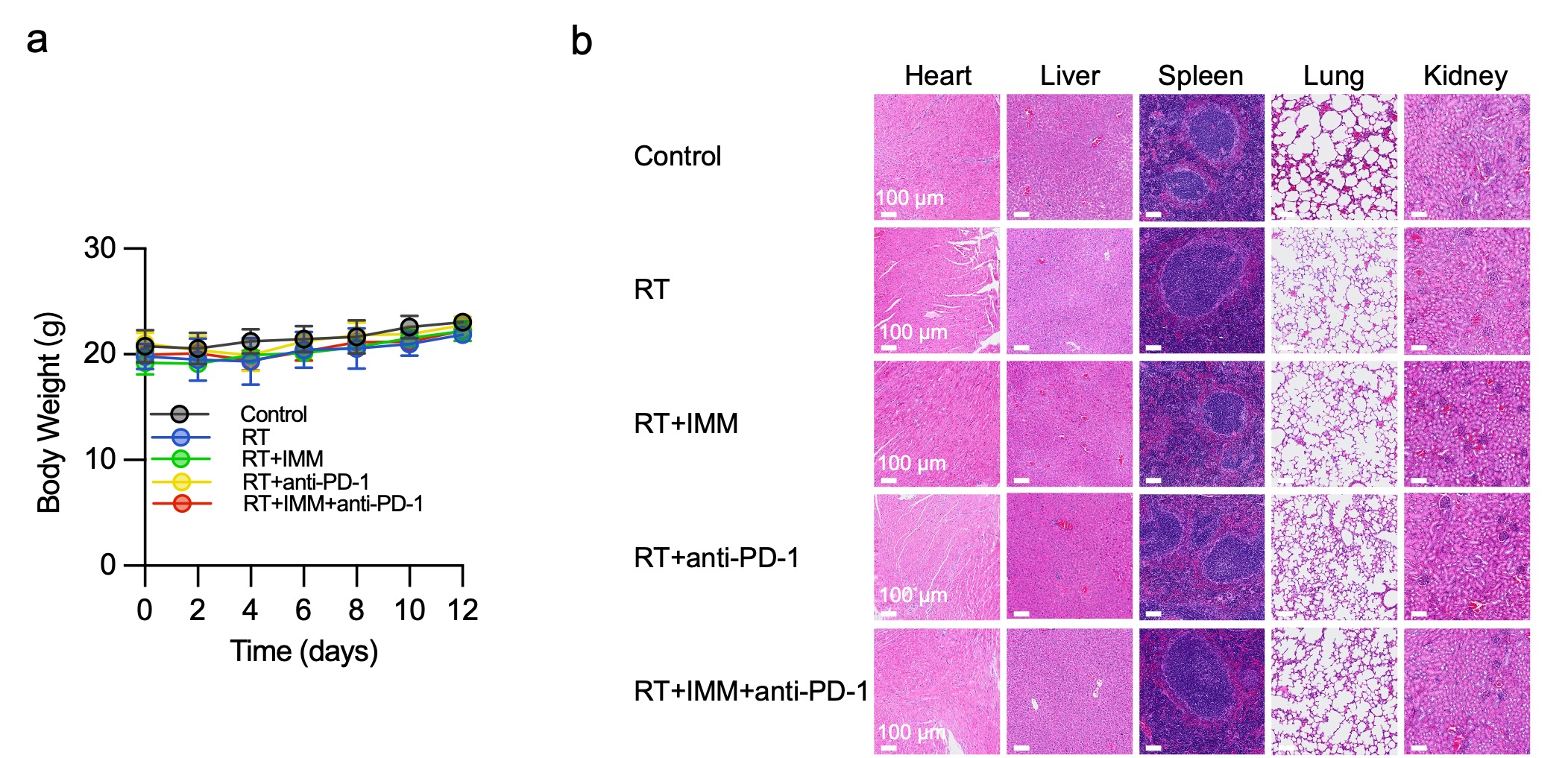
**

**Fig. S31** *In vivo* safety evaluation of different treatments. a) Body weight changes of BALB/c mice after treatments (n = 5 biologically independent mice per group). b) Representative H&E-stained tissue sections from each group (scale bar = 100 μm). Experiments were repeated independently three times with similar results.

**
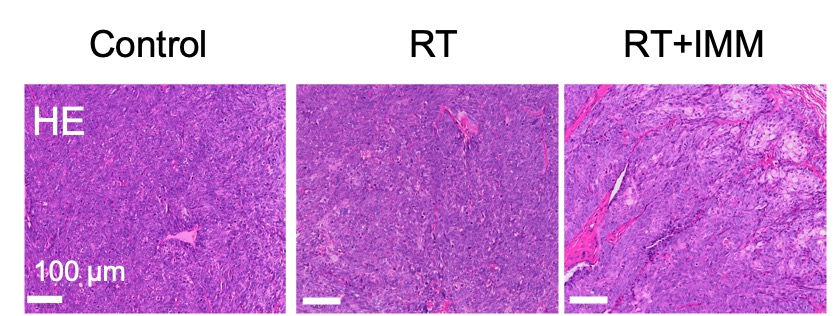
**

**Fig. S32** Representative H&E-stained tissue sections from each group in humanized PDX models (scale bar = 100 μm). Experiments were repeated independently three times with similar results.


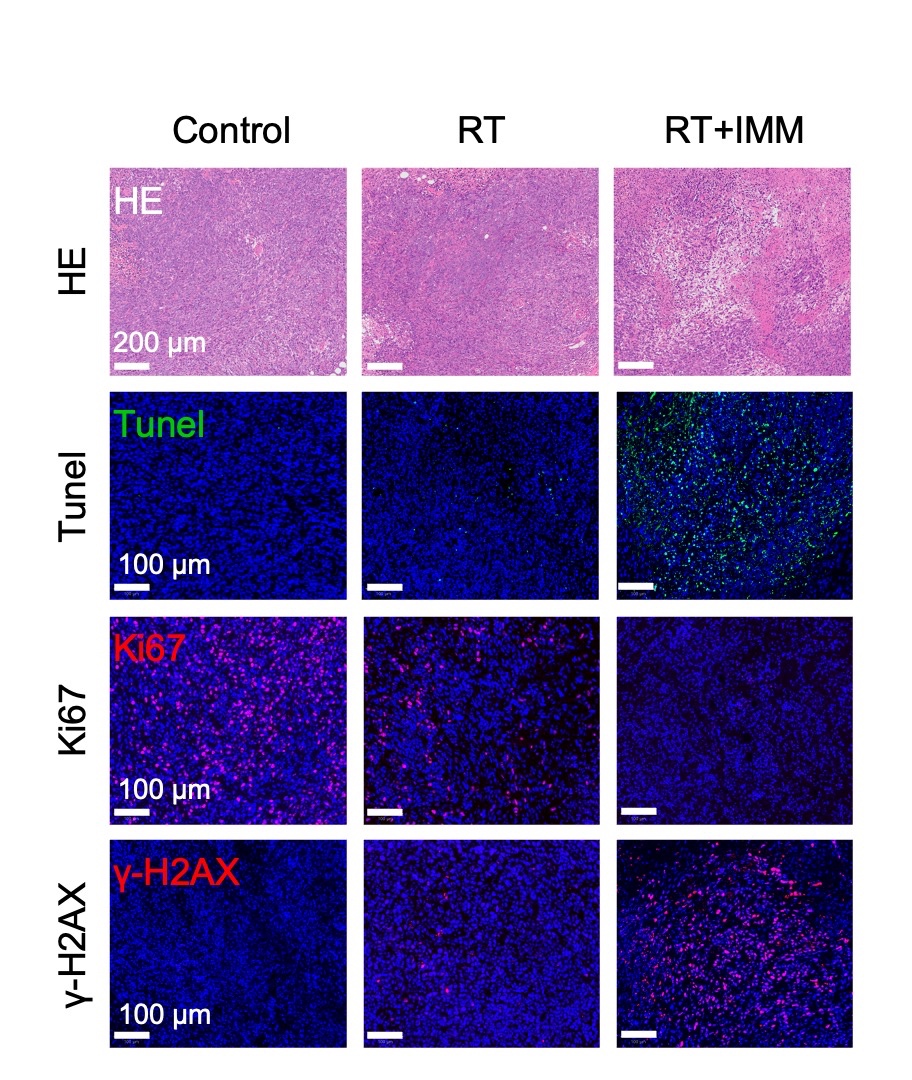


**Fig. S33** *In vivo* evaluation of antitumor efficacy for different treatments in a mouse breast cancer model. Representative images of H&E-stained (scale bar = 200 μm), TUNEL assay, γ-H2AX detection, and Ki67 detection tissue sections from each group (scale bar = 100 μm). Experiments were repeated independently three times with similar results.

**S3 Supporting Tables**

**Table S1** The content of the Mn oxidation state for different samples was determined by XPS spectra.

| Samples  Content (%) | MM | IMM |
| --- | --- | --- |
| Mn^2+^ | 48.28 | 41.11 |
| Mn^4+^ | 35.37 | 42.24 |
| Sat. | 16.35 | 16.65 |

**Table S2** Comparison of the kinetics of IMM and Ir/C.

|  | E_0_ (µM) | *V*_max_ (µM s^-1^) | *K*_m_ (mM) | TON (×10^-3^ s^-1^) |
| --- | --- | --- | --- | --- |
| IMM | 17.6 | 2.37 | 0.22 | 134.7 |
| Ir/C | 19.7 | 1.44 | 1.49 | 73.2 |

**Table S3** Extended X-ray absorption fine structure (EXAFS) fitting parameters at the Ir *L*-edge for various samples (*Ѕ*_0_^2^ = 0.73).

| Sample | Shell | *N^a^* | *R*(Å)*^b^* | *σ*^2^(Å^2^)*^c^* | Δ*E*_0_(eV)*^d^* | *R* factor |
| --- | --- | --- | --- | --- | --- | --- |
| Ir foil | Ir-Ir | 12.00 | 2.71 | 0.0029 | 8.247 | 0.017 |
| IMM | Ir-O | 2.93 | 2.00 | 0.0051 | 10.00 | 0.021 |
|  | Ir-Ir | 6.33 | 2.68 | 0.0059 |  |  |
|  | Ir-Mn | 4.00 | 3.70 | 0.0553 |  |  |
| IrO_2_ | Ir-O | 6.68 | 1.98 | 0.0030 | 11.61 | 0.021 |
|  | Ir-Ir | 2.50 | 3.13 | 0.0025 |  |  |

*^a^N*: coordination numbers; *^b^R*: bond distance; *^c^σ*^2^: Debye-Waller factors; ^d^Δ*E*_0_: the inner potential correction. *R* factor: goodness of fit. *Ѕ*_0_^2^ was set to 0.73, according to the experimental EXAFS fit of Ir foil by fixing coordination number as the known crystallographic value.

**Table S4** Comparison of the POD-mimetic catalytic kinetic constants of IMM to H_2_O_2_ and other reported artificial enzymes.

| Biocatalysts | *V*_max_ (μΜ s^-1^) | TON (10^-3^ s^-1^) | Ref. |
| --- | --- | --- | --- |
| IMM | 2.37 | 134.7 | This work |
| Cu-AME | 0.81 | 101.04 | [S11] |
| Fe-AME | 1.01 | 98.65 | [S11] |
| Fe SAEs | 0.083 | 77.10 | [S12] |
| Fe-Art-M | 0.115 | 74.00 | [S13] |
| Ir/C | 1.44 | 73.2 | This work |
| Ru_NC_/BTO | 0.319 | 63.29 | [S14] |
| Ir-AME | 0.370 | 54.18 | [S11] |
| Ir_NCs_@Ti-MOF | 1.700 | 39.64 | [S15] |
| V-Art-M | 0.051 | 27.30 | [S13] |
| VO_x_-AE | 0.260 | 26.00 | [S16] |
| V-Fe_2_O_3_ | 1.070 | 22.38 | [S17] |
| a-RuS_2_ | 2.980 | 16.30 | [S18] |
| Cu_2_O-CN_x_@CeO_2_ | 0.484 | 14.67 | [S19] |
| Pt hollow nanodentrites (Pt HNs) | 0.100 | 14.14 | [S20] |
| Pd@Pt core-frame nanodendrities  (Pd@Pt CFNs) | 0.090 | 12.857 | [S20] |
| V_2_O_5_ | 0.126 | 9.000 | [S16] |
| Ru NPs | 0.580 | 5.86 | [S21] |
| Cu(OH)_2_ | 0.340 | 3.332 | [S22] |
| Cu NPs | 0.09 | 3.30 | [S23] |
| CeO_2_ | 0.180 | 3.096 | [S22] |
| CuO | 0.280 | 2.226 | [S22] |
| Pt NCs | 0.182 | 1.366 | [S24] |
| Fe_3_O_4_ | 0.160 | 1.237 | [S22] |
| Fe_2_O_3_ | 0.068 | 0.544 | [S22] |
| ZnO | 0.07 | 0.27 | [S16] |

**Supplementary References**

1. G. Kresse, J. Hafner, *Ab initio* molecular-dynamics simulation of the liquid-metal–amorphous-semiconductor transition in germanium. Phys. Rev. B **49**(20), 14251–14269 (1994). <https://doi.org/10.1103/PhysRevB.49.14251>.
2. G. Kresse, J. Furthmüller, Efficiency of ab-initio total energy calculations for metals and semiconductors using a plane-wave basis set. Comput. Mater. Sci. **6**(1), 15–50 (1996). <https://doi.org/10.1016/0927-0256(96)00008-0>.
3. G. Kresse, J. Furthmüller, Efficient iterative schemes for *ab initio* total-energy calculations using a plane-wave basis set. Phys. Rev. B **54**(16), 11169–11186 (1996). <https://doi.org/10.1103/PhysRevB.54.11169>.
4. P.E. Blöchl, Projector augmented-wave method. Phys. Rev. B **50**(24), 17953–17979 (1994). <https://doi.org/10.1103/PhysRevB.50.17953>.
5. J.P. Perdew, J.A. Chevary, S.H. Vosko, K.A. Jackson, M.R. Pederson, et al., Atoms, molecules, solids, and surfaces: Applications of the generalized gradient approximation for exchange and correlation. Phys. Rev. B Condens. Matter **46**(11), 6671–6687 (1992). <https://doi.org/10.1103/physrevb.46.6671>.
6. J. Klimeš, D.R. Bowler, A. Michaelides, Van der Waals density functionals applied to solids. Phys. Rev. B **83**(19), 195131 (2011). <https://doi.org/10.1103/PhysRevB.83.195131>.
7. J. Klimeš, D.R. Bowler, A. Michaelides, Chemical accuracy for the van der Waals density functional. J. Phys. Condens. Matter Inst. Phys. J. **22**(2), 022201 (2010). <https://doi.org/10.1088/0953-8984/22/2/022201>.
8. R.F.W. Bader, A quantum theory of molecular structure and its applications. Chem. Rev. **91**(5), 893–928 (1991). <https://doi.org/10.1021/cr00005a013>.
9. W. Tang, E. Sanville, G. Henkelman, A grid-based Bader analysis algorithm without lattice bias. J. Phys. Condens. Matter **21**(8), 084204 (2009). <https://doi.org/10.1088/0953-8984/21/8/084204>.
10. V. Wang, N. Xu, J.-C. Liu, G. Tang, W.-T. Geng, VASPKIT: A user-friendly interface facilitating high-throughput computing and analysis using VASP code. Comput. Phys. Commun. **267**, 108033 (2021). <https://doi.org/10.1016/j.cpc.2021.108033>.
11. S. Cao, Z. Zhao, Y. Zheng, Z. Wu, T. Ma, et al., A Library of ROS-Catalytic Metalloenzyme-Mimics with Atomic Metal Centers. Adv. Mater. **34**(n/a), 2200255 (2022). <https://doi.org/https://doi.org/10.1002/adma.202200255>.
12. C. Zhao, C. Xiong, X. Liu, M. Qiao, Z. Li, et al., Unraveling the enzyme-like activity of heterogeneous single atom catalyst. Chem. Commun. **55**(16), 2285–2288 (2019). <https://doi.org/10.1039/c9cc00199a>.
13. Y. Long, L. Li, T. Xu, X. Wu, Y. Gao, et al., Hedgehog artificial macrophage with atomic-catalytic centers to combat Drug-resistant bacteria. Nat. Commun. **12**(1), 6143 (2021). <https://doi.org/10.1038/s41467-021-26456-9>.
14. H. Zhu, J. Deng, M. Yuan, X. Rong, X. Xiang, et al., Semiconducting Titanate Supported Ruthenium Clusterzymes for Ultrasound-Amplified Biocatalytic Tumor Nanotherapies. Small **19**(18), e2206911 (2023). <https://doi.org/10.1002/smll.202206911>.
15. S. Mu, Y. Deng, Z. Xing, X. Rong, C. He, et al., Ir Cluster-Anchored MOFs as Peroxidase-Mimetic Nanoreactors for Diagnosing Hydrogen Peroxide-Related Biomarkers. ACS Appl. Mater. Interfaces **14**, 56635 (2022). <https://doi.org/10.1021/acsami.2c18676>.
16. L. Li, S. Cao, Z. Wu, R. Guo, L. Xie, et al., Modulating Electron Transfer in Vanadium-Based Artificial Enzymes for Enhanced ROS-Catalysis and Disinfection. Adv. Mater. **34**(17), 2108646 (2022). <https://doi.org/https://doi.org/10.1002/adma.202108646>.
17. H. Huang, W. Geng, X. Wu, Y. Zhang, L. Xie, et al., Spiky Artificial Peroxidases with V-O-Fe Pair Sites for Combating Antibiotic-Resistant Pathogens. Angew. Chem. Int. Ed. **63**(1), e202310811 (2024). <https://doi.org/10.1002/anie.202310811>.
18. T. Li, Y. Deng, Z. Xing, S. Xiao, S. Mu, et al., Amorphization-Modulated Metal Sulfides with Boosted Active Sites and Kinetics for Efficient Enzymatic Colorimetric Biodetection. Small Methods **7**(7), e2300011 (2023). <https://doi.org/10.1002/smtd.202300011>.
19. L. Cao, Z. Feng, R. Guo, Q. Tian, W. Wang, et al., The direct catalytic synthesis of ultrasmall Cu2O-coordinated carbon nitrides on ceria for multimodal antitumor therapy. Mater. Horiz. **10**(4), 1342–1353 (2023). <https://doi.org/10.1039/d2mh01540d>.
20. C. Ge, R. Wu, Y. Chong, G. Fang, X. Jiang, et al., Synthesis of Pt Hollow Nanodendrites with Enhanced Peroxidase-Like Activity against Bacterial Infections: Implication for Wound Healing. Adv. Funct. Mater. **28**(28), 1801484 (2018). <https://doi.org/10.1002/adfm.201801484>.
21. G.-J. Cao, X. Jiang, H. Zhang, T.R. Croley, J.-J. Yin, Mimicking horseradish peroxidase and oxidase using ruthenium nanomaterials. RSC Adv. **7**(82), 52210–52217 (2017). <https://doi.org/10.1039/C7RA10370K>.
22. X. Wang, X.J. Gao, L. Qin, C. Wang, L. Song, et al., eg occupancy as an effective descriptor for the catalytic activity of perovskite oxide-based peroxidase mimics. Nat. Commun. **10**(1), 704 (2019). <https://doi.org/10.1038/s41467-019-08657-5>.
23. Y. Wu, J. Wu, L. Jiao, W. Xu, H. Wang, et al., Cascade Reaction System Integrating Single-Atom Nanozymes with Abundant Cu Sites for Enhanced Biosensing. Anal. Chem. **92**(4), 3373–3379 (2020). <https://doi.org/10.1021/acs.analchem.9b05437>.
24. L. Jin, Z. Meng, Y. Zhang, S. Cai, Z. Zhang, et al., Ultrasmall Pt Nanoclusters as Robust Peroxidase Mimics for Colorimetric Detection of Glucose in Human Serum. ACS Appl. Mater. Interfaces **9**(11), 10027–10033 (2017). <https://doi.org/10.1021/acsami.7b01616>.
